# Radiologic assessment of quality of root canal fillings and periapical status in an Austrian subpopulation – An observational study

**DOI:** 10.1371/journal.pone.0176724

**Published:** 2017-05-02

**Authors:** Andrej M. Kielbassa, Wilhelm Frank, Theresa Madaus

**Affiliations:** 1 Centre for Operative Dentistry, Periodontology, and Endodontology, University of Dental Medicine and Oral Health, Danube Private University (DPU), Krems, Austria; 2 Centre for Preclinical Education, Deptartment of Biostatistics, University of Dental Medicine and Oral Health, Danube Private University (DPU), Krems, Austria; University of Washington, UNITED STATES

## Abstract

**Background/Objective:**

Progress in endodontic techniques and methodological advances have altered root canal therapy over the last decades. These techniques and methods need periodical documentation. This observational study determined the current prevalence of endodontic treatments, and investigated the relationship of various factors with the periapical status in a Lower Austrian subpopulation.

**Methodology:**

One thousand orthopantomograms of first-time university adult patients radiographed at an outpatient clinic were evaluated. For each tooth, the presence of periradicular pathosis and/or endodontic treatment was recorded, as was the quality of (post-)endodontic treatment (homogeneity and length of root canal fillings; preparation failures; posts/screws; apicoectomies; coronal restorations). Two evaluators, blinded to each other, scored all teeth. In cases of disagreement, they joined for a consensus score.

**Results:**

In all, 22,586 teeth were counted. Of these, 2,907 teeth (12.9%) had periapical pathosis, while 2,504 teeth had undergone root canal treatment. Of the endodontically treated teeth, 52% showed no radiographic signs of apical periodontitis, while 44.9% had overt apical lesions, and 3,1% revealed widened periodontal ligament space. The majority of the root canal fillings was inhomogeneous (70.4%); 75.4% were rated too short, and 3.8% too long. The presence of apical pathosis was significantly correlated (odds ratio (OR) 2.556 [confidence interval (CI) 2.076–3.146]; *P*<0.0001) with poor root canal fillings (length and homogeneity). Posts or screws positively affected periapical status (OR 1.853 [CI 1.219–2.819]; *P* = 0.004), but endodontically treated posterior teeth were infrequently restored (posts, 7.5%; screws, 2.7%). Best results were found for teeth with both appropriate endodontic treatment and adequate coronal restoration.

**Conclusion:**

A high prevalence of periradicular radiolucencies was observed with root canal filled teeth, along with high numbers of unmet treatment needs. Periapical health was associated with adequate root canal obturation and high-grade postendodontic restorations, and quality regarding these latter aspects is considered mandatory to promote periapical health.

## Introduction

From a radiographic point of view, several criteria for success of endodontic therapy are generally accepted. These include healing (or at least regression) of recent osseous rarefication, normal (or only slightly thickened) periodontal ligament space, normal lamina dura, missing evidence of resorption, and a dense and homogeneous three-dimensional obturation of the root canal system, including a sufficient coronal restoration [1, 2]. Due to the aetiological impact of the microbial infection [3], the aspects given above are clinically achieved by a complete bacterial eradication (or at least by a significant reduction) of viable pathogenic micro-organisms from the root canal [4].

In the last decades, advances in technology, along with procedural improvements, have changed endodontic therapy, but, with regard to many studies, this obviously did not affect success rates of root canal therapy over the years [5]. Thus, a recently published systematic review of observational studies [6] reported a varying treatment quality with regard to prevalence of apical periodontitis in root canal treated teeth, with an overall weighted mean of 35.9% (± 10.1%). When compiling the major international studies (each including more than 10,000 teeth, and with a total of some 20,000 root canal filled teeth) over the last six years [7–16], mean frequencies of apical pathoses amounted to a comparable pooled average rate of some 33%, thus confirming constant failure rates of root canal filled teeth (see Table 1).

**Table 1:** Prevalence of apical pathosis in various countries (compilation of the major and most recent observational studies per region).

Region	Country	[Reference]	Total teeth (N)	Teeth with AP (n)	Teeth with AP (%)	RCF teeth (n)	RCF teeth with AP (n)	RCF teeth with AP (%)
Africa	Nigeria	[7]	21,468	3,083	14.36	2,625	1,068	40.69
South America	Brazil	[8]	25,292	1,700	6.72	1,754	293	16,70
Asia	India	[9]	30,098	1,759	5.84	1,234	462	37.44
	Iran	[10]	28,463	N/A	N/A	1,013	527	52.02
Europe	Croatia	[11]	38,440	3,251	8.46	3,279	1,772	54.04
	Cyprus	[12]	24,730	1,734	7.01	2,200	1,364	62.00
	Finland	[13]	120,635	5,335	4.42	7,986	1,222	15.30
	Turkey	[14]	28,974	647	2.23	459	193	42.05
	Turkey	[15]	11,542	189	1.64	179	68	37.99
	Turkey	[16]	23,268	287	1.23	601	95	15.81
	**Total**		352,910	17,985		21,330	7,064	
	**Pooled average**				**5.10**			**33.12**

AP (apical pathosis); RCF (root canal filled).

Several observational studies determined the frequency of (in-)adequately filled root canals in relation to healthy periapical areas, and epidemiological data have shown different treatment outcomes in various regions of the world, with prevalences of inadequate root canal fillings of up to 72.4%, and with 87.0% of these teeth showing apical periodontitis [17]. Without doubt, the quality of the root canal treatment (and, hence, a tight coronal and apical seal of the shaped and cleaned root canal) is not considered the only aspect to ensure a good outcome of endodontic treatment. In addition to appropriately performed root canal fillings, adequate quality of the respective postendodontic restorations would seem to be an essential prerequisite to prevent reinfection, and to positively affect healing of apical periodontitis [2, 18, 19], thus increasing endodontic success and tooth survival [20].

Periodic assessments of the prevalence of apical pathosis in different populations may help to define treatment needs in a specific region and relate treatment outcome to various factors. With regard to Central European (German-speaking) populations, however, only limited information on quality and quantity of endodontic treatments has been published over the last decade [21], and this in particular comes true for Austria [22]. Thus, the aim of the present study was to assess the prevalence of apical periodontitis in a Lower Austrian subpopulation. In addition, the qualities of root canal fillings as well as those regarding the respective postendodontic coronal restorations were evaluated with regard to their impact on periapical health, thus testing the null hypothesis (H_0_) of missing correlations between the mentioned factors. H_0_ was tested against the alternative hypothesis of an interdependency of the variables (H_A_).

## Materials and methods

This investigation was ethically in accordance with the Helsinki Declaration of 1964 (as revised and amended in its ninth version in 2013) [23]. Approval of the study protocol by the central Ethical Review Board of the federal state of Lower Austria (vote number: GS1-EK-4/328-2015) was obtained, and all participants of this observational study gave their written consent for participation and the use of their respective data for research purposes. Blindness of evaluators (AMK, TM) was assured by coding of radiographs, and an independent administration secretary ascertained this procedure. With the present report, we adhered to the STROBE statement on reporting cross-sectional (observational) studies [24] (S1 Checklist).

### Study population

All patients attending the Danube Private University’s (DPU) outpatient clinic for the first time (in 2013 and 2014) and undergoing panoramic radiography (OPG) for general reasons (like diagnosis and treatment planning) [25] were counted in consecutive order; age and sex of the patients were recorded. For the present study every second OPG of different subjects (starting from the first one) was chosen (resulting in 1,000 OPGs, and representing 1,000 different patients); thus, a high participation level was ensured, and selection bias was minimised. This study included dentate subjects who had one or more teeth or root remnants based on panoramic radiographs, while neither impacted teeth nor wisdom teeth were recorded. Patients under 18 years and toothless subjects were excluded. In these cases the next patient matching the selection criteria was considered eligible.

### Radiographic evaluation

Every digital orthopantomogram was made with a customary, licensed X-ray machine (Orthophos XG 3D; Sirona Dental Systems, Bensheim, Germany); all evaluations were performed under standardised conditions (darkened room, without any daylight) using a calibrated diagnostic screen (EIZO Flex Scan S2202W; EIZO, Vienna, Austria). Two examiners (AMK, TM) independently evaluated the selected OPGs (October/November 2015) after internal calibration, and any discordant radiologic findings were re-assessed and resolved by re-appraisal and discussion, thus reaching a mutual consent between the two assessors. Data sheets were used to record gender and age of the patients; moreover, after counting the presence or absence of teeth (number and location of teeth in total), the number and location of teeth without root canal fillings revealing identifiable periradicular lesions was recorded. Root canal fillings were registered (every root displaying radiopaque materials was considered endodontically treated), and number and location of endodontically treated teeth without identifiable periapical lesions, as well as number and location of endodontically treated teeth revealing periradicular radiolucencies or radiopacities were recorded. Third molars were not included in the present study.

#### Evaluation of root canal fillings

By using previously described criteria [26], we differentiated between adequate (all canals completely and homogeneously obturated, and root canal fillings ending from 0 to 2 mm short of the radiographic apex; no voids visible) and inadequate endodontic fillings (with radiopaque materials present in the pulp chamber only or (slightly) beyond, or ending more than 2 mm short of the radiographic apex, or grossly overfilled; root canal fillings considered inhomogeneous, and presenting voids, inadequate density, and/or poor condensation). The scorings for multi-rooted teeth were determined by the most incomplete or inadequate filling per single root canal.

#### Evaluation of periradicular status

In due consideration of previously published criteria with regard to the periapical status [13], the radiographic appearance of the root apices and their surrounding structures was assessed by combining several aspects disclosing periapical disease. Thus, we distinguished between healthy teeth (contour and width of the periodontal ligament space without detectable abnormalities; and surrounding bone without any radiologic findings) and diseased teeth. The latter were defined as revealing discernible periradicular radiolucencies or radiopacities in connection with the apical part of the root, and/or periodontal ligament space more than doubled in width, and/or loss or disruption in the continuity of the lamina dura [15], and these radiographic findings were separately assessed to minimise possible subjectivity and bias. Additionally, evaluation referred to periapical index (PAI) scores 3, 4, and 5 (changes in bone structure, including radiographic evidence of mineral loss up to well defined radiolucent areas of severe apical pathosis), thus focusing on conventional failure/success assessment [27]. With multi-rooted teeth, the most diseased root identified the respective score.

#### Evaluation of coronal restorations

The quality of coronal restorations was assessed on the basis of the radiologic evaluation. Again, described criteria [16] were used to differentiate between adequate restorations (permanent fillings, partial or full crowns appearing intact from a radiographic point of view) and inadequate restorations (missing or obviously temporary fillings; permanent restorations revealing shortcomings like overhangs, open margins, or radiolucencies in terms of caries).

#### Evaluation of secondary findings

Furthermore, complications due to preparation failures (perforation, *via falsa*, broken instruments, and/or straightening of canals) were recorded. Presence of screws and (fibreglass) posts were compiled, and root surgeries were captured.

### Statistical analysis

In cases of discordant gradings (n = 31 of the cases), a consensus score was agreed between both evaluators. Cohen’s Kappa (κ) was calculated to determine inter-rater reliability, and the latter was considered substantial to (almost) perfect [28], with κ values ranging from 0.696 (root surgery) to 0.889 (coronal restoration). Raw data (including missing cases due to non-assessable radiographic aspects [S1 File]) were entered into Excel sheets (Microsoft; Redmond, WA, USA), and all analyses were carried out using a statistical software package (SPSS 23.0; IBM, Armonk, NY, USA). Frequencies were calculated, and comparisons of the latter were performed using Chi-squared tests, thus allowing for analysis of any association between periradicular health and root canal filling (length and homogeneity), and the relationship between apical pathosis and technical quality of the endodontic treatment, posts/screws, or coronal restoration. These comparisons were based on the unequivocal scores of the two evaluators. Risk of apical pathosis was estimated by means of odds ratios (OR) and the corresponding 95% confidence intervals (with the root canal filled tooth representing the unit of analysis), and measures of association (Phi [*Φ*] and Cramér’s V) between the dichotomous and qualitative variables were calculated. Treatment outcome was assessed by multiple logistic regression analyses to identify predictors of apical pathosis. The significance level α was set at 5%. A post hoc power calculation analysis was conducted with respect to a detectable relationship between the root canal treatment and apical radiolucency at a power level of 0.8 (SAS 9.3; Cary, NC, USA).

## Results

The mean (± SD) age of the participating dentate subjects was 49.9 (± 16.7) years (range = 19–91 years; median = 52 years); 43.0% were men (for age and gender distribution, see Table 2), with a total of 22,586 teeth being assessed. The mean number of teeth per subject was 12.7 (± 6.4) for women, and 9.88 (± 6.05) for men; with regard to the presence of periapical health, only 40% of the patients showed no signs of apical disease.

**Table 2:** Distribution of patients and (root canal filled) teeth according to age and gender.

Age (years)	Gender	All patients (N)	Patients with AP (n)	All teeth (N)	Teeth with AP (n)	Teeth with AP (%)	RCF teeth (n)	RCF teeth with AP (n)	RCF teeth with AP (%)
**18–29**	Male	83	24	2,298	55	2.4	50	29	58.0
	Female	89	18	2,449	44	1.8	59	29	49.2
**30–39**	Male	50	25	1,317	50	3.8	81	41	50.6
	Female	57	29	1,496	60	4.0	98	45	45.9
**40–49**	Male	69	53	1,759	125	7.1	199	99	49.7
	Female	104	62	2,556	148	5.8	268	104	38.8
**50–59**	Male	83	61	1,900	160	8.4	251	113	45.0
	Female	146	107	3,076	258	8.4	490	203	41.4
**60–69**	Male	85	63	1,588	172	10.8	297	129	43.4
	Female	97	70	1,908	184	9.6	374	133	35.6
**>70**	Male	60	41	1,017	88	8.7	142	63	44.4
	Female	77	52	1,222	110	9.0	195	78	40.0
**Total**		**1,000**	**605**	**22,586**	**1,454**	**6.4**	**2,504**	**1,066**	**42.6**

AP (apical pathosis); RCF (root canal filled).

With regard to radiographic peculiarities like periradicular pathoses, 1,454 teeth were identified, thus representing 6.4% of all investigated teeth. A total of 2,504 teeth had undergone root canal treatment, while 388 teeth (1.7%) revealing apical disease unequivocally did not show any signs of endodontic treatment (Table 2). Percentage apical pathosis was increased with the higher age groups. Women’s periradicular health was considered significantly better than men’s apical status (*p* = 0.001; multivariate model); in all age groups, they revealed more teeth than men, with continuously higher numbers of endodontic treatments and proportionately lower percentages of root canal filled teeth with apical pathosis (Table 2).

None of the orthopantomograms was discarded; however, in cases of poor areal radiographic quality the respective aspects were considered non-judgeable by both assessors (numbers given in Table 3, together with a compilation of other variables of interest). Root canal fillings ending from 0 to 2 mm short of the radiographic apex were observed with 499 teeth (20.8%), while 724 (29.6%) of the fillings were considered homogeneous. In contrast, 1,809 teeth (75.4%) revealed root canal fillings ending more than 2 mm short of the radiographic apex (*i*.*e*. only the most coronal part of the root canal was filled, thus denoting former attempts at endodontic procedures), and 90 (3.8%) of the teeth were (grossly) overfilled (revealing some kind of material beyond the apex) (Table 3). With regard to radiographic density, 1,721 teeth (70.4%) revealed inhomogeneous root canal fillings (*e*.*g*., voids, inadequate density, and/or poor compaction; Table 3).

**Table 3:** Distribution of evaluated variables with root canal filled teeth (descriptive statistics including non-assessable aspects).

Variable	Condition	Number (n)	Percent (%)
**Root canal filled teeth**	Yes	2,504	86.1
	No (apical pathosis only, including 15 non-assessable cases)	403	13.9
**Length of root canal filling**	0 to 2 mm short of apex	499	20.8
(non-assessable: 106)	Under-filling	1,809	75.4
	Over-filling	90	3.8
**Homogeneity of root canal filling**	Adequate	724	29.6
(non-assessable: 59)	Inhomogeneous	1,721	70.4
**Preparation failure**	Yes	194	8.0
(non-assessable: 82)	No	2,228	92.0
**Post or screw**	Post	153	7.5
(non-assessable: 53)	Screw	55	2.7
	Too short	441	21.6
	No	1,391	68.2
**Root surgery**	Yes	126	5.1
(non-assessable: 41)	No	2,337	94.9
**Coronal restoration**	Fillings	1,052	52.7
**(premolars and molars only)**	Partial crowns	24	1.2
(non-assessable: 96)	Crowns	921	46.1
**Quality of coronal restoration**	Adequate	1,180	46.5
(non-assessable: 368)	Inadequate/missing	1,359	53.5
**Widened periodontal space ligament**	Yes	1,315	48.0
(non-assessable: 165)	No	1,427	52.0
**Apical pathosis**	Yes	1,454	52.4
(non-assessable: 131)	No	1,322	47.6

Periodontal ligament space was widened in 1,315 (48.0%) cases, while 1,427 (52.0%) teeth did not reveal any pathological findings (both contour and width of periodontal ligament space were considered normal; Table 3). Teeth with widened periodontal ligaments showed additional signs of apical radiolucencies in 717 cases, while apical periodontitis was present with 544 teeth without widened periodontal ligament space.

**Fig 1:**
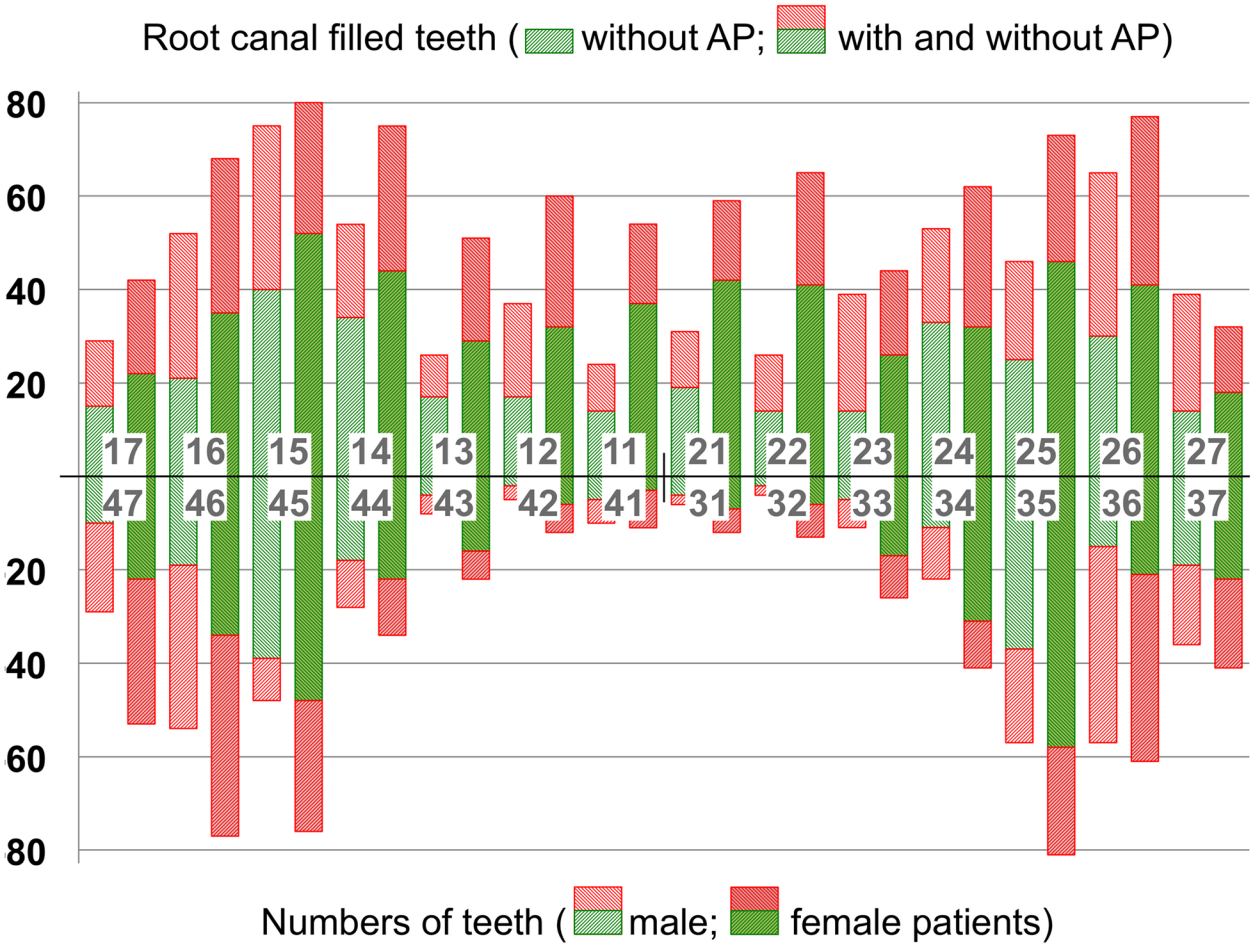
Distribution of apical pathosis (AP). Distribution is given by type of tooth, by jaw, and by gender for all root canal filled teeth (n = 2,373; note that total number of teeth represents unequivocal scores of the evaluators).

In total, 1,555 root canal treated teeth (62.1%) were present in the upper jaw (while 949 teeth (37.9%) were mandibular); no significant differences could be revealed between maxillary and mandibular teeth (*p* = 0.096; multivariate model). There were less endodontically treated anterior teeth (723 (28.9%)) if compared to molars (840 (33.5%)) and premolars (941 (37.6%)); in particular, only small numbers of root canal treated mandibular incisors could be observed. Female subjects persistently revealed more root canal filled teeth if compared to male participants, and the proportion of apical health was constantly higher with each tooth type if compared to men. The numbers of root canal filled teeth (with and without periradicular pathoses, and broken down by tooth types and upper/lower jaws) are given in Fig 1.

Regarding endodontic treatment, 1,053 (44.4%) teeth showed signs of apical radiolucencies (while 13 (0.5%) teeth revealed periradicular radiodensities); in contrast, 1,307 (55.1%) of the teeth with root canal fillings revealed no radiologic evidence of periradicular inflammation (Table 4). With an odds ratio (OR) of 0.032, the odds of apical periodontitis with root canal filled teeth was 32 times less than those for missing endodontic treatments (*P* < 0.0001), meaning that root canal treatment revealed a protective effect. The contingency coefficient of correlation *Φ* was 0.36, indicating a strong relationship between apical pathosis and endodontic treatment, with more periradicular health in root canal filled teeth (Table 4). When focusing on the quality of root canal fillings, the odds ratios of apical disease with inadequate lengths (OR = 1.639) or non-homogeneous obturations (OR = 1.599) indicated increased chances (64% and 60%, respectively) to reveal apical pathosis. The respective Phi coefficients revealed weak associations between length (*Φ* = 0.10) or homogeneity (*Φ* = 0.11) of the root canal fillings and periapical health (Table 4).

**Table 4:** Periradicular status in relation to various factors.

Covariate	N (%)	No apical pathosis n (%)	Apical pathosis n (%)	*P* value*	OR (CI 95%)	*Φ*
**Endodontically treated teeth**				<0.0001	0.032 (0.019–0.053)	0.36
No	403 (14.5)	15 (3.7)	388 (96.3)			
Yes	2,373 (85.5)	1,307 (55.1)	1,066 (44.9)			
**Length of root canal filling (length)**				<0.0001	1.639 (1.331–2.019)	0.10
Adequate (0 to 2 mm)	482 (20.9)	313 (64.9)	169 (35.1)			
Inadequate (short of apex/overfilled)	1,821 (79.1)	966 (53.0)	855 (47.0)			
**Homogeneity of root canal filling**				<0.0001	1.599 (1.334–1.918)	0.11
Adequate	702 (30.0)	444 (63.2)	258 (36.8)			
Inadequate	1,640 (70.0)	850 (51.8)	790 (48.2)			
**Preparation failure**				<0.0001	2.261 (1.666–3.069)	0.11
No	2,129 (91.7)	1,214 (57.0)	915 (43.0)			
Yes	192 (8.3)	71 (37.0)	121 (63.0)			
**Post or screw**				<0.0001	0.529 (0.437–0.641)	0.15
No	1,346 (68.1)	559 (41.5)	787 (58.5)			
Yes (including short lengths)	630 (31.9)	361 (57.3)	269 (42.7)			
**Root surgery**				0.115	1.391 (0.922–2.098)	0.03
No	2,263 (96.0)	1,258 (55.6)	1,005 (44.4)			
Yes	95 (4.0)	45 (47.4)	50 (52.6)			
**Coronal restoration**				0.002	0.752 (0.628–0.899)	0.07
Fillings	1,018 (52.7)	445 (43.7)	573 (56.3)			
(Partial) crowns	915 (47.3)	465 (50.8)	450 (49.2)			
**Quality of coronal restoration**				<0.0001	1.800 (1.532–2.115)	0.15
Adequate	1,123 (46.0)	618 (55.0)	505 (45.0)			
Inadequate or missing	1,317 (54.0)	533 (40.5)	784 (59.5)			

* χ^2^ test; P values < 0.05 are considered statistically significant;

OR (odds ratio); CI (confidence interval); note that total number of teeth represents unequivocal scores of the evaluators;

Interpretation of Φ values: 0.00 to 0.09 = no/negligible; 0.10 to 0.19 = (very) weak; 0.20 to 0.29 = moderate; 0.30 to 0.39 = strong correlation.

Preparation faults (recorded as perforation, *via falsa*, fractured instruments, and/or straightening of root canals) were found in 194 (8.0%) cases, while 2,228 (92.0%) teeth revealed no signs of instrumentation failures (Table 3). Overall, 121 (63.0%) teeth with preparation failures were associated with periapical pathosis. Teeth with preparation failures diseased 2.26 times more often than teeth without failures, while statistical analysis indicated a weak interdependency between endodontic incidents and apical pathosis (*Φ* = 0.11; Table 4).

Of the 153 (7.5%) teeth restored with a post (with adequate length), 90 teeth (58.8%) revealed no signs of apical radiolucencies, while periradicular pathosis was present in 23 (41.8%) of the teeth treated with a screw showing adequate length (n = 55; 2.5%). No tooth was restored adhesively with a fibreglass post. Posts and screws considered too short were associated with periapical pathosis in 190 (43.9%) of the posterior teeth, while 243 (56.1%) teeth did not reveal any apical radiolucency. When combining post and screws to one group (including the anchors considered short), an odds ratio of 0.529 was calculated; here, the odds for periapical health was 1.89 times less without the presence of posts or screws (if compared to teeth with post and core restorations). The corresponding Phi coefficient revealed a weak association between the binary variables (*Φ* = 0.15; Table 4).

Root surgery was present in 126 (5.1%) of the evaluated teeth (Table 3). Of these teeth, 50 (52.6%) revealed apical radiolucencies, while 45 (47.4%) apicoectomized teeth showed no signs of periradicular pathosis. Odds ratio was 1.391, indicating that root surgery increased the chance of apical pathosis to 39% (if compared to teeth without root surgery); however, this was not statistically different (*p* = 0.116), and the Phi coefficient did not denote any systematic pattern across the contingency table (*Φ* = 0.03; Table 4).

As for the coronal restorations of the posterior dentition (n = 2,093), 1,052 (52.7%) teeth showed no reinforcing restorations (such as partial crowns or full crowns); instead, conventional fillings (composite resin, amalgam, or glass ionomer cements) were recorded (Table 3). Of these teeth, 573 (56.3%) teeth showed apical periodontitis (Table 4). Only the small number of 24 (1.2%) teeth was treated with a partial crown, with apical radiolucencies in 14 (58.3%) of the cases. Crowns as postendodontic restorations were found in 921 (46.1%) of the evaluated teeth, and 450 (49.2%) of these revealed periradicular pathoses. With an odds ratio of 0.752, the odds of apical periodontitis with postendodontic restorations (like partial and full crowns) were 1.3 times less than those with conventional fillings (*p* = 0,002); however, calculation of Phi revealed a negligible relationship (*Φ* = 0.07; Table 4). In contrast, the quality of the postendodontic restoration was weakly associated with periapical health (OR = 1.800; *Φ* = 0.15).

Only 226 (9.9%) of the evaluated teeth revealed root canal fillings considered as adequate with regard to both length and homogeneity. Of these, 147 (65.0%) were scored as healthy (Table 5). In contrast, 674 (50.3%) of the teeth rated as inadequately filled were associated with periradicular pathosis. With an odds ratio of 2.556, the odds of periapical health with homogeneous and adequate length of the root canal fillings was more than doubled if compared inadequate quality of root canal fillings (*P* < 0.0001). The corresponding measure of association (Cramér’s V = 0.18) revealed a weak relationship between endodontic quality and periapical health (Table 5).

**Table 5:** Periradicular status in relation to various combined factors ().

Covariate	N (%)	No apical pathosis n (%)	Apical pathosis n (%)	*P* value*	OR (CI 95%)	Cramér’s V
**Quality of root canal filling (length and homogeneity)**				<0.0001	2.556 (2.076–3.146)	0.18
Adequate length/homogeneous filling	226 (9.9)	147 (65.0)	79 (35.0)			
Inadequate length/homogeneous filling	465 (20.3)	291 (62.6)	174 (37.4)			χ^2^ = 81.25
Adequate length/inhomogeneous filling	255 (11.2)	165 (64.7)	90 (35.3)			df 1
Inadequate length/inhomogeneous filling	1,341 (58.6)	667 (49.7)	674 (50.3)			
**Quality of endodontic treatment combined with post/screw**				0.004	1.853 (1.219–2.819)	0.07
Adequate treatment/Post or screw	31 (1.9)	19 (61.3)	12 (38.7)			
Adequate treatment/no post or screw	93 (5.6)	58 (62.4)	35 (37.6)			χ^2^ = 8.55
Inadequate treatment/Post or screw	590 (35.4)	340 (57.6)	250 (42.4)			df 1
Inadequate treatment/no post or screw	955 (57.2)	489 (51.2)	466 (48.8)			
**Quality of endodontic treatment and coronal restoration**				<0.0001	1.789 (1.329–2.408)	0.09
Adequate treatment/adequate restoration	128 (6.2)	90 (70.3)	38 (29.7)			
Inadequate treatment/adequate restoration	892 (43.4)	519 (58.2)	373 (41.8)			χ^2^ = 15.05
Adequate treatment/inadequate restoration	77 (3.7)	43 (55.8)	34 (44.2)			df 1
Inadequate treatment/inadequate restoration	960 (46.7)	480 (50.0)	480 (50.0)			

* χ^2^ test; P values < 0.05 are considered statistically significant;

OR (odds ratio); CI (confidence interval); note that total number of teeth represents unequivocal scores of the evaluators;

Interpretation of Cramér’s V: 0.00 to 0.09 = no/negligible; 0.10 to 0.19 = (very) weak; 0.20 to 0.29 = moderate; 0.30 to 0.39 = strong correlation.

Metallic posts or screws in combination with adequate root canal fillings were associated with radiographic inconspicuousness; the odds of missing periapical radiolucencies in cases with adequate root canal fillings combined with posts and screws was 1.85 times that of the other combinations (*p* = 0.004; Cramér’s V = 0.07; Table 5).

When combining qualities of root canal treatments and postendodontic restorations (and comparing these merged data with periradicular status), we observed that 90 (70.3%) of the 128 cases were rated as periapically sound for both adequate endodontic treatments and adequate restorations. Accordingly, when pooling inadequate root canal fillings and inadequate restorations, 480 (50.0%) of the evaluated teeth (n = 960) revealed the highest prevalence of diseased teeth (Table 5). With an odds ratio of 1.789, the odds of apical health with adequate treatment (both adequate root canal treatment and adequate coronal restoration) were increased by 79% if compared to inadequate treatment modalities. The strength of the relationship between periradicular health and fully adequate treatments was negligible (Cramér’s V = 0.09; Table 5).

**Fig 2:**
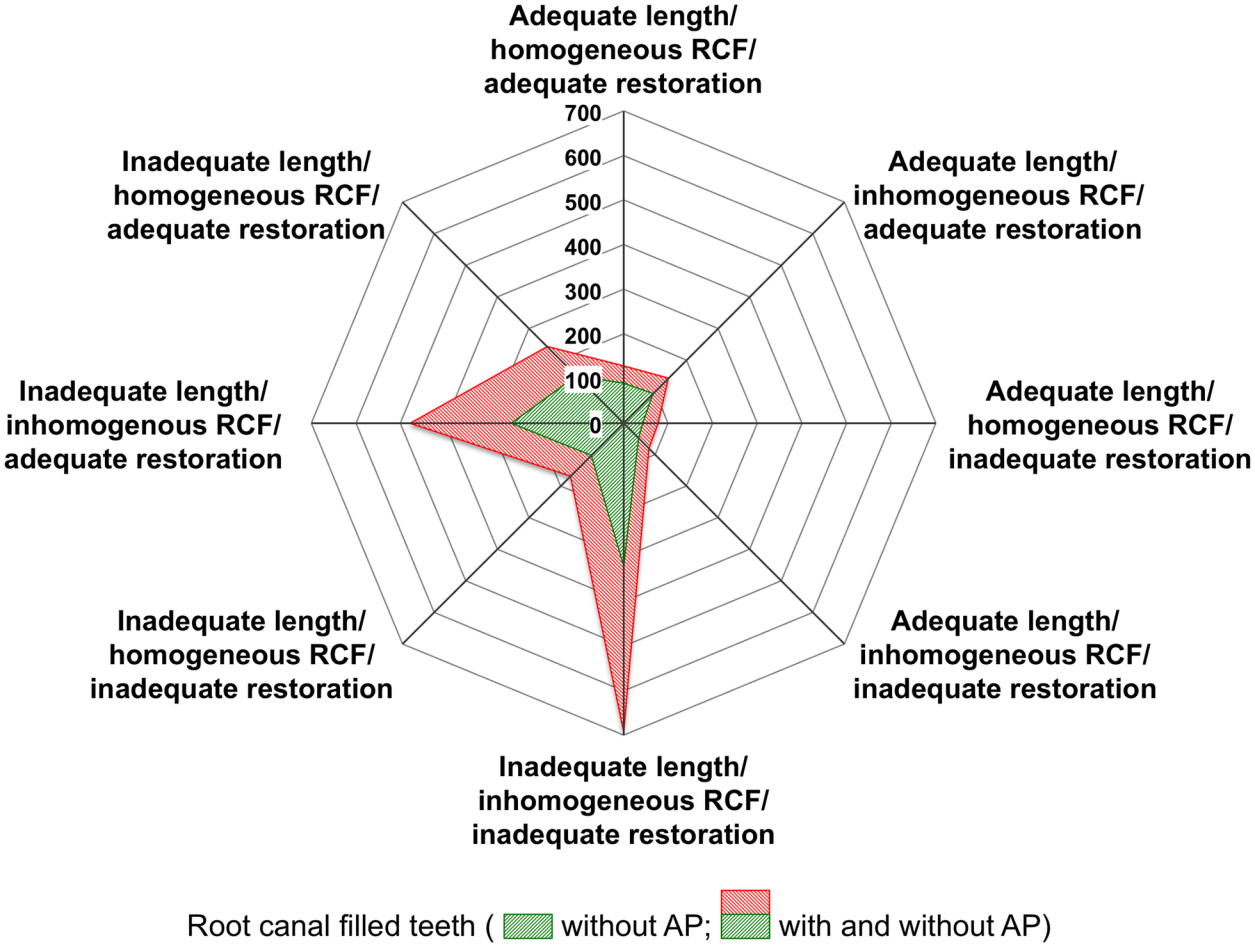
Kiviat diagram visualising the multivariate observations. Total amount of cases per combination is indicated by numbers (0–700). Inadequate length and inhomogeneous quality of root canal filling (RCF) strongly increased the proportion of apical pathosis (AP), and this was again tremendously affected by poor quality of coronal restoration (n = 2,007; note that total number of teeth represents unequivocal scores of the evaluators).

Fig 2 displays the outcome of the multivariate treatment modalities in the form of a two-dimensional chart of the eight possible therapeutic pairwise combinations (length/homogeneity of root canal filling; quality of coronal restoration) with respect to apical health and periradicular pathosis. Inadequate lengths and inhomogeneities of root canal fillings as well as poor restorations increased the portion of periradicular pathoses. However, when comparing different subgroups (adequate endodontic treatments; adequate coronal restorations) with their inadequate counterparts, the quality of the coronal restoration did not reveal any significant effects (*p* = 0.306; univariate model).

## Discussion

In Austria, compulsory health insurance applies to paid employees, most self-employed persons, patients claiming unemployment benefits, pensioners, and to the respective dependants (spouses, registered partners, and children and adolescents up to the age of 18 years or serving an apprenticeship). With registered 734 dentists (as of May 2016), Lower Austria has a dentist-population ratio of approximately 1:2,200. Most practitioners are general dentists, and specialisation to subdisciplines is not widely spread. Thus, endodontic services are available in both the public and the private sectors, and the respective fees are covered free of charge if the patients consult a dentist having a contract with the health insurance fund. The district of Krems and its suburban parishes, along with the surrounding countryside regions (Cider/Must Quarter, Wine Quarter, Wachau Cultural Landscape, Forest Quarter/Bohemian Plateau), has a population of approximately 100,000 inhabitants; the region’s economic focus lies on wine and fruit growing, along with tourism. This is not representative of metro areas, but the region is well comparable to other climes. The Danube Private University’s outpatient clinic opened in 2013; thus, the present study included exclusively patients coming from community settings, or those having formerly been treated in doctors’ surgeries.

The current outcome was based on systematic random sampling (starting with the first patient attending the outpatient clinic, and selecting every second subject, pursuant to the chronological order). This procedure assured uniform sampling, and different patterns of trait present in the population could be excluded. With some 50% of all adult and dentate patients registered and radiographed at the outpatient clinic, an adequate proportion took part in the current evaluation (which is considered to represent a 1-in-2 sample, with a good approximation to simple random sampling).

Undoubtedly, the cross-sectional nature (measuring the cumulative condition of a sub-population, but not the incidence of disease) of the present investigation has some limitations. With its static “snapshot” character in mind, each orthopantomogram evaluated in the current study did solely allow for a statement on a certain point of time, and did not permit any predications on future developments of the observed periradicular pathoses. Therefore, individual prognoses on exacerbations, though conceivable, are not possible, nor would it be appropriate to make binding statements on persistence or healing status of the radiographic lesions. This also applies to the onset of apical periodontitis; whether the therapeutic intervention was performed before or after onset of the disease, will remain unknown, and to infer any causalities from the present study would seem speculative. However, it should be kept in mind that the number of healed periapical periodontitis approximately equals to the number of newly developed lesions [29, 30]. Hence, more than half of all evaluated periapical pathoses should rank among chronic or new lesions, confirming that observational evaluations of large numbers of orthopantomograms indicate a realistic scenario and do permit an estimation of the overall oral health and the prevalence of both disease and treatment in a given (sub-)population [31].

The quality of timely digital panoramic radiographs has been described to be sufficient for detecting apical pathoses and endodontically treated teeth if compared to full-mouth radiographic series even in the anterior region [13] (while higher sensitivities and accuracies have been ascribed to cone-beam computed tomography [32], thus considered suitable to disclose even higher prevalences of apical periodontitis, if compared to conventional (digital) 2D radiographs [33]). However, contrary to other recent investigations [34], teeth scored as PAI 1 and 2 were not included in the present study [14], and we pooled scores 3 to 5 [35]. Therefore, we focussed on unambiguous radiographic signs, and tried to compensate for the negative predictive value (0.67) of radiographs for apical periodontitis [36]. Notwithstanding, the current results (even though consented after observational ratings with high concordance, and with an accordingly decreased risk of failure caused by subjective interpretations of single examiners) presumably represent underdiagnosed pathoses and underestimated prevalence of periapical disease, in particular with regard to frequencies and dimensions of mandibular incisors and canines [37]. This might explain the results given in Fig 1.

Due to radiographic faults, some teeth were considered non-assessable, and the respective proportions ranged from 0.25% to 2.2%, depending on the respective subgroups of the current investigation. This was in good agreement with previous reports [35, 38], and lower if compared to investigations using conventional radiography [39]. Regarding the categories length and homogeneity of endodontic treatment, coronal restoration, and periapical status the inter-rater reliability as assessed by Cohen’s κ was satisfying, and this was in accordance with previous studies [7, 10, 40].

Evaluating treatment qualities in a selected population (and, thus, mapping the present situation of a given disease) may constitute a basis for establishment of educational intervention strategies and consecutively ameliorated healthcare services. With this in mind, and when reflecting on the results of the present study, evaluation of radiographs is but recording quality of outcome, even if the assessment of root canal fillings might be used as (surrogate) indicator of the overall technical standards to some extent. It should be emphasized, however, that this information does not shed any light on the actual procedural qualities of the particular endodontic treatment. These intra-operative prerequisites (*e*.*g*., use of rubber dam, instrumentation, chemo-mechanical preparation using disinfecting irrigants, and use of local medicaments as inter-appointment dressings) do have enormous impact on the success rates of endodontic therapy, even if the influence of the single factors on the overall outcome has not been well studied up to now [1].

The ultimate objective of endodontic treatment is to prevent or heal periradicular pathosis. Conversely, endodontic failure would include all root canal treated teeth being clinically symptomatic, and/or radiographically revealing periapical lesions (either new ones or those increasing in size or having failed to heal). However, terms like ‘success’ and ‘failure’ (as well as the adjectives ‘effective’ and ‘ineffective’), would seem to refer to treatment (and its various modalities) only, while the perception of ‘healed’, ‘healing’, and ‘non-healed’ lesions does acknowledge patient-centred factors [41], and this is considered a timely approach to individualized medicine. Interestingly, it has been argued that ‘survival’ would be a better measure of treatment outcome [42], and this reflects to treatments providing patients without functional impairment or pain of any single tooth. Indeed, some patients seem to favour this point of view, and cases of asymptomatic lesions and uncertain or incomplete healing might be classified as ‘survivals’ compatible with health (or ‘survivals with need to intervene’) instead of true failures [43]. Undoubtedly, controlled and continued monitoring might be justified in some individual cases [41], but it seems worth emphasizing that generalized differentiation between disease and illness is not unequivocally indicated, and must cause an ethical dilemma.

Notwithstanding, this approach would seem feasible with homogeneous root canal fillings of adequate extensions only [1], even if density *per se* has not been standardised, and is difficult to score on radiographs [13, 14, 44]. In the present study, homogeneity of the fillings was recorded in 29.6% of the cases; more favourable results with radiographically homogeneous root canal fillings of up to 91.9% have been reported in the literature [11, 45, 46]. However, the effect of the homogeneity of the root canal filling on the periapical status has been controversially discussed; from Fig 2 and Table 4 it seems clear that poor compaction was of minor importance if compared to previous studies [14, 45]. Moreover, with only 20.8% of the root canal fillings reaching the length within 0 to 2 mm short of the radiographic apex, the current outcome clearly trailed the expectations set by previous studies [44, 45].

Asymptomatic persistent apical periodontitis associated with shortcomings regarding the root canal filling are highly suspicious of deterioration, and might require intervention [42], depending on the accompanying risk factors [47]. In addition, care should be taken when focusing on remained function or high numbers of tooth retention only, since apical pathoses may result from both inflammatory and non-inflammatory origin [48] without any subjective symptoms for the patient, not always being resolved by conventional root canal treatment, and often calling for histopathologic clarification. Moreover, pulpal inflammation and persistence of chronic apical periodontitis are associated with a highly complex bacterial community [49], and have recently been reported to be independently associated with development of coronary heart disease [50, 51], thus potentially increasing the patient’s total vascular risk [52–54]; this undoubtedly would call for clinical vigilance.

As has been mentioned above, 2,504 teeth were recognized as root canal treated in the present study; when focusing on the quality of the endodontic treatment, there were 79 teeth (corresponding to 35.0% of the group with both adequate radiographic length and adequate homogeneity of the root canal filling) showing periradicular pathoses. At the same time, 667 teeth (49.7% of those revealing inhomogeneously filled root canals with inadequate lengths) were considered healthy. This would seem contradictory, and might enlighten the fact that the radiographic appearance of root canal fillings obviously is only an aspect of reserved importance; other facets of standard operative care refer to an extensive elimination of any root canal infection, and to the prevention of (re-)contamination during (and after) treatment [3, 4]. The present study showed that the odds ratio to develop apical periodontitis with inadequate root canal treatment was increased by 155% if compared to those with adequate root canal treatment (Table 5). However, as has already been emphasized above, this result does not reveal any signs on the procedural quality during the treatments.

A high prevalence of periradicular pathosis and poor technical quality of root canal treatment was found in the sub-population studied, and apical pathoses were more than doubled in root canal filled (n = 1,066) compared with non-treated teeth (n = 388). Out of the total, the burden of disease was 4.7% in root-filled teeth and 1.7% in teeth without root canal fillings, thus being comparable to previous studies [6, 9, 16, 44]. At a first glance, this would suggest that root canal treatment does not control the disease [10, 46]. However, adequately root canal filled teeth had a significantly lower prevalence of apical pathosis than inadequately treated teeth (35.0% vs. 50.3%, respectively; see Table 5), and this relation seems to corroborate other observations [55, 56].

Overall, 1,066 (44.9%) of the endodontically treated teeth were considered diseased in the present study, and this prevalence clearly outmatched the weighted averages (36.0%) calculated from a recent systematic review compiling 33 cross-sectional studies [6]; likewise, this frequency clearly exceeded the values known from reports published more recently (compare Table 1). The highest portion of apical pathoses was observed in association with faults during preparation of root canals; the latter could be assessed in 121 out of 194 teeth. In addition, with a high portion of periradicular disease in apicoectomized teeth, root surgery was frequently associated with apical pathosis. On the contrary, it should be borne in mind that when starting with exclusively diseased teeth, surgery improved periapical health, at least to some extent.

The present study showed that 11.1% of all evaluated teeth had undergone endodontic treatment. This is in accordance with previous investigations having reported similar results (ranging from 8.8% to 13.4%) [45, 57]. Altogether, 44.9% of the endodontically treated teeth were associated with apical periodontitis, and this again corresponded well with results from other studies [7, 9, 14, 15, 58, 59]. Accordingly, of the 2,504 evaluated teeth, only 226 (9.9%) showed adequate endodontic treatments (regarding both homogeneity and length of root canal filling; see Table 5). These data disclosed a substandard quality of endodontic treatments, and this was clearly below the outcomes of previous investigations revealing adequate root canal treatments with up to 40.2% of the cases [31, 60], even if the parameters used to score endodontic quality were not the same for all studies. Thus, although a conclusive differentiation between healing lesions and those having failed is not possible with observational studies, this outcome clearly demonstrates at least a high portion of intervention needs (amounting to some 50% of all apically diseased teeth when focusing on periradicular pathoses only, and to 90.1% when additionally including teeth with poor technical quality of root canal fillings).

Postendodontic restorations using posts have not been deemed beneficial with regard to the onset of apical pathosis [34, 47, 61]. In the present investigation, 7.5% of the posterior teeth were provided with a post, and this again was comparable to recent reports [61, 62]. Interestingly, statistical analysis revealed an association between posts and periapical health, and root canal filled teeth without a post showed reduced odds compared to those with a post to develop periradicular radiolucency (see Table 5). In other words, placing a post increased the odds of periapical health for 85%. A comparable tendency has been reported elsewhere [12], and this might be explained by the tight seal provided by posts, and/or by bactericidal corrosive deposits sealing the post/root canal interface. However, the latter aspect would be suitable for metallic posts and screws only (and not for adhesively luted fibreglass posts, since these could not be observed with the current outcome), and future studies are warranted to elucidate these issues.

A recent meta-analysis demonstrated increasing odds for healing of apical pathosis with adequate quality of both root canal treatment and restorative treatment, along with poor clinical outcomes in case of combinations of inadequate aspects [2]. Moreover, insufficient coronal restorations obviously increase the prevalence of apical pathosis [10, 12, 16, 18, 19, 44, 62–66], and this was corroborated with the present findings (compare Fig 2). In the current investigation, 128 (6.2%) of the endodontically treated teeth revealed adequate homogeneity, adequate length of the root canal filling, and simultaneously adequate coronal restoration; apical pathosis was present in 29.7% of these cases. In contrast, the combination of inadequate root canal filling and inadequate restoration (960 teeth (46.7%)) was not associated with periapical inflammation in 50.0% of the cases (Table 5).

All in all, the null hypothesis of the present study, assuming that there would be no correlation between the quality of the root canal treatment (length and homogeneity of the root canal fillings as well as failures in preparation), the quality of postendodontic restorations (treatment with posts or dental screws; sufficiency of the restoration) and the radiographic finding of periapical osteolysis was rejected. The number of teeth with apical pathoses amounted to 6.4% of all teeth (while nearly every second endodontically treated tooth showed radiolucencies, and some 90% of the root canal fillings showed shortcomings necessitating re-treatment). This finding clearly exceeded previously reported endodontic treatment needs [67–69] (compare Table 1), and would seem to re-emphasize the challenges in responding to the diversity of urgent world-wide oral health needs [70].

## Conclusion

With the limitations of observational studies in mind, it is concluded that the technical quality of root canal fillings and the quality of the postendodontic coronal restorations in Lower Austria are poor. Apical disease in root canal filled teeth is in the upper range of similar data collected elsewhere in the world, and the present study documented a high portion of unmet treatment needs. The current findings call for improvements in the technical quality of endodontic treatment and coronal restorations. Increasing the understanding of the etiology of apical periodontitis [71, 72] is necessary to provide better endodontic services, to Austrian as well as to other populations.

## Supporting information

S1 Checklist STROBE statement. (PDF)

S1 File Dataset (raw data). (PDF)
