# Supplementary material for: Radiologic assessment of quality of root canal fillings and periapical status in an Austrian subpopulation – An observational study
Source: PLoS One. 2017 May 2;12(5):e0176724. doi: 10.1371/journal.pone.0176724 (PMC5413016; doi:10.1371/journal.pone.0176724)
Supplement: S1 File — (PDF) [file pone.0176724.s002.pdf]

patient-code	age	gender	number of teeth in total	numb. endod. treated teeth	Teet with AP	tooth	eT	aP	wpS	H	LF
1.	53	w	22	5	0	16	1	0	1	1	1
	53	w				11	1	0	1	1	0
	53	w				25	0	0	1	99	99
	53	w				35	1	0	0	0	1
	53	w				34	1	0	0	0	1
	53	w				41	1	0	0	0	0
	53	w				47	0	0	1	99	99
8.	45	w	22	8	4	16	1	0	0	1	1
	45	w				15	1	0	0	0	0
	45	w				14	1	1	0	1	1
	45	w				26	1	1	0	0	1
	45	w				34	1	1	0	1	1
	45	w				42	1	0	0	0	1
	45	w				44	1	0	0	1	1
	45	w				45	1	1	0	1	1
16.	62	m	22	14	7	17	1	0	99	1	99
	62	m				16	1	0	99	2	1
	62	m				15	1	0	0	1	1
	62	m				13	1	0	0	1	1
	62	m				12	1	0	0	1	1
	62	m				21	1	0	0	1	1
	62	m				22	1	0	0	1	0
	62	m				23	1	1	0	1	1
	62	m				24	0	1	0	99	99
	62	m				25	1	0	1	1	1
	62	m				37	0	1	0	99	99
	62	m				36	0	1	1	99	99
	62	m				41	1	1	1	0	1
	62	m				42	1	0	0	0	1
	62	m				44	1	1	0	1	1
	62	m				45	1	0	0	0	1
	62	m				47	1	1	0	1	1
33.	42	w	22	4	2	16	1	0	0	1	1
	42	w				12	1	1	0	0	1
	42	w				21	1	0	0	1	0
	42	w				26	1	1	0	1	1
37.	65	w	11	2	3	24	1	0	1	1	1
	65	w				44	0	1	1	99	99
	65	w				45	0	1	1	99	99
	65	w				47	1	1	0	1	1
41.	24	m	27	2	2	15	1	1	1	1	0
	24	m				46	1	1	1	0	0
43.	42	m	28	1	1	16	1	1	0	1	0
44.	61	w	20	3	1	36	1	0	1	1	1
	61	w				34	1	1	1	0	0
	61	w				32	1	0	0	0	0
47.	77	w	13	5	2	13	1	0	0	0	1
	77	w				12	1	1	0	2	1
	77	w				11	1	0	0	2	1
	77	w				21	1	0	0	0	0
	77	w				44	1	1	0	2	1
52.	46	m	28	1	0	35	1	0	0	0	2
53.	73	m	13	2	0	13	1	0	0	1	1
	73	m				44	1	0	0	0	0
55.	53	w	21	1	0	23	1	0	1	1	0
56.	46	m	18	2	2	23	1	1	0	1	0
	46	m				36	1	1	0	1	1
58.	55	m	19	11	5	16	1	0	1	1	1
	55	m				13	1	1	1	1	1
	55	m				12	1	1	0	0	0
	55	m				37	1	0	1	1	1
	55	m				36	1	1	1	1	1
	55	m				34	1	1	0	0	2
	55	m				43	1	1	0	1	0

	55	m				44	1	0	1	1	1
	55	m				45	1	0	0	1	1
	55	m				46	1	0	0	1	0
	55	m				47	1	0	0	1	0
69.	38	w	27	2	1	36	1	1	0	1	1
	38	w				45	1	0	0	1	1
71.	38	w	24	8	4	17	1	0	0	1	1
	38	w				13	1	0	0	0	1
	38	w				12	1	1	1	0	1
	38	w				21	1	0	1	0	1
	38	w				22	1	0	1	1	1
	38	w				24	1	1	0	1	1
	38	w				25	1	1	0	1	1
	38	w				36	1	1	0	1	1
79.	33	w	28	2	1	22	1	0	0	0	1
	33	w				47	1	1	0	1	1
81.	66	w	25	2	2	25	1	1	0	1	1
	66	w				27	1	1	1	1	1
83.	68	w	24	4	5	13	1	0	0	1	1
	68	w				14	0	1	1	99	99
	68	w				24	0	1	1	99	99
	68	w				12	0	1	1	99	99
	68	w				36	1	1	1	1	1
	68	w				35	1	0	0	1	1
	68	w				46	1	1	0	0	1
90.	75	w	12	5	2	22	1	0	1	1	1
	75	w				33	1	0	0	0	1
	75	w				32	1	1	1	1	0
	75	w				31	1	1	1	0	0
	75	w				42	1	0	0	0	0
95.	31	m	28	0	1	36	0	1	0	99	99
96.	34	m	27	2	1	15	1	0	1	1	1
	34	m				36	1	1	0	1	1
98.	26	w	26	3	0	14	1	0	0	0	1
	26	w				25	1	0	1	1	1
	26	w				34	1	0	1	0	2
101.	51	w	23	1	0	15	1	0	0	0	0
102.	30	w	28	2	1	16	1	0	0	1	0
	30	w				46	1	1	0	1	1
104.	44	m	28	1	0	17	1	0	0	1	1
105.	57	w	12	1	1	33	1	0	1	1	1
	57	w				46	0	1	1	99	99
107.	59	m	19	1	0	23	1	0	1	0	1
108.	56	w	24	3	0	11	1	99	0	0	0
	56	w				21	1	99	0	0	0
	56	w				25	1	0	0	1	1
111.	29	w	26	4	1	22	1	0	1	1	0
	29	w				37	1	0	0	0	2
	29	w				36	1	0	1	0	0
	29	w				46	1	1	1	1	1
115.	72	m	16	6	3	13	1	1	0	1	1
	72	m				22	1	0	0	0	1
	72	m				23	1	0	1	1	1
	72	m				24	1	0	0	0	0
	72	m				32	0	1	1	99	99
	72	m				44	1	1	0	1	1
	72	m				45	1	0	1	1	1
122.	26	m	27	3	1	21	1	0	0	1	0
	26	m				36	1	0	0	0	1
	26	m				45	1	1	1	1	1
125.	56	w	26	1	0	47	1	0	1	0	1
126.	47	m	27	2	1	35	1	0	0	0	1
	47	m				46	1	1	0	1	1
128.	70	w	28	2	2	21	1	0	0	0	1
	70	w				17	0	1	1	99	99
	70	w				36	0	1	1	99	99
	70	w				27	1	0	0	0	0
132.	67	m	25	5	1	14	1	0	0	1	1
	67	m				22	1	1	0	1	1
	67	m				24	1	99	0	1	1
	67	m				26	1	0	0	1	1
	67	m				27	1	99	0	1	1
137.	76	m	16	7	3	16	1	0	99	1	1
	76	m				14	1	1	0	1	1
	76	m				36	1	0	0	1	1
	76	m				35	1	1	1	0	1
	76	m				34	1	1	0	0	1

	76	m				32	1	0	1	1	1
	76	m				46	1	0	1	1	1
144.	63	m	17	9	2	13	1	0	0	0	0
	63	m				12	1	0	0	2	1
	63	m				21	0	1	1	99	99
	63	m				11	1	0	1	1	0
	63	m				22	1	0	0	1	1
	63	m				23	1	1	0	1	1
	63	m				37	1	0	0	0	1
	63	m				35	1	0	0	0	1
	63	m				42	1	0	0	1	2
	63	m				44	1	0	1	1	1
154.	35	w	12	2	1	33	1	0	1	1	1
	35	w				13	0	1	1	99	99
	35	w				43	1	0	0	1	0
157.	54	m	18	7	3	16	1	0	1	1	1
	54	m				14	1	0	1	1	1
	54	m				12	1	1	1	1	1
	54	m				11	1	0	0	1	1
	54	m				21	1	0	0	1	1
	54	m				36	1	1	0	1	2
	54	m				35	1	1	0	1	1
164.	63	w	25	5	2	14	1	0	1	1	1
	63	w				25	1	0	0	0	1
	63	w				45	1	0	0	0	1
	63	w				46	1	1	0	1	1
	63	w				47	1	1	0	1	0
169.	67	m	18	1	0	37	1	0	0	2	1
170.	55	w	24	1	0	45	1	0	1	0	1
171.	63	w	16	3	1	21	1	99	99	99	99
	63	w				36	1	1	1	1	1
	63	w				45	1	0	0	1	0
174.	24	m	27	4	9	14	1	0	1	1	1
	24	m				12	1	99	1	1	1
	24	m				11	1	1	0	1	1
	24	m				22	1	1	0	1	1
	24	m				23	0	1	1	99	99
	24	m				36	0	1	1	99	99
	24	m				34	0	1	1	99	99
	24	m				33	0	1	1	99	99
	24	m				32	0	1	1	99	99
	24	m				42	0	1	1	99	99
	24	m				43	0	0	1	99	99
	24	m				45	0	0	1	99	99
	24	m				46	0	1	1	99	99
187.	46	w	24	1	2	25	1	1	0	1	1
	46	w				46	0	1	0	99	99
189.	45	w	26	7	2	24	1	0	0	1	1
	45	w				25	1	0	0	1	1
	45	w				37	1	0	1	1	1
	45	w				36	1	1	1	1	0
	45	w				35	1	0	0	1	1
	45	w				45	1	0	0	1	1
	45	w				47	1	1	1	1	0
196.	23	m	28	1	0	45	1	0	0	0	0
197.	52	w	6	1	1	33	1	1	0	1	1
198.	60	m	5	3	0	14	1	0	0	1	1
	60	m				13	1	0	1	0	1
	60	m				26	1	0	0	1	0
201.	42	w	26	1	0	16	1	0	1	1	1
202.	22	m	28	0	1	46	0	1	1	99	99
203.	74	m	13	2	2	25	1	1	0	1	1
	74	m				36	0	1	1	99	99
	74	m				44	1	0	1	1	1
206.	37	m	26	6	2	12	1	1	0	1	1
	37	m				11	1	99	0	1	2
	37	m				26	1	0	0	1	1
	37	m				27	1	0	0	1	1
	37	m				35	1	0	0	0	0
	37	m				46	1	1	1	1	1
212.	50	m	18	4	1	17	1	0	0	1	1
	50	m				16	1	0	99	1	1
	50	m				23	1	1	0	0	1
	50	m				25	1	99	1	0	1
216.	60	w	18	9	0	15	1	0	0	1	1
	60	w				21	1	99	99	1	1
	60	w				22	1	99	99	1	1

	60	w				23	1	99	99	1	1
	60	w				24	1	99	99	1	1
	60	w				26	1	99	1	1	1
	60	w				35	1	0	1	1	1
	60	w				34	1	0	1	0	1
	60	w				47	1	0	0	1	1
225.	57	w	20	2	0	11	1	0	0	1	1
	57	w				31	1	0	0	0	0
227.	59	w	26	2	1	15	1	0	0	1	1
	59	w				14	1	1	99	2	1
229.	70	w	9	3	1	44	1	99	1	1	99
	70	w				45	1	1	1	1	99
	70	w				46	1	0	1	1	1
232.	68	m	22	2	1	15	1	1	1	1	2
	68	m				14	1	0	1	1	1
234.	62	w	26	4	2	11	1	99	99	0	0
	62	w				26	1	1	0	1	1
	62	w				35	1	1	0	0	1
	62	w				45	1	0	1	0	1
238.	61	m	12	3	0	16	1	0	1	1	2
	61	m				13	1	0	1	1	1
	61	m				21	1	0	1	0	0
241.	73	w	24	1	1	45	1	1	0	1	1
242.	48	w	24	9	3	16	1	0	1	1	1
	48	w				15	1	0	1	0	0
	48	w				14	1	1	99	1	99
	48	w				12	1	0	1	0	1
	48	w				11	1	0	0	1	0
	48	w				21	1	0	0	1	1
	48	w				22	1	1	0	1	0
	48	w				25	1	1	1	1	0
	48	w				26	1	0	0	1	1
251.	58	w	21	7	1	15	1	0	1	0	1
	58	w				14	1	0	1	1	1
	58	w				12	1	0	0	0	0
	58	w				11	1	0	1	0	1
	58	w				24	1	0	0	1	1
	58	w				25	1	0	1	0	1
	58	w				47	1	1	0	1	1
258.	50	w	24	7	1	17	1	0	1	1	1
	50	w				16	1	1	1	1	1
	50	w				14	1	0	1	1	1
	50	w				25	1	99	99	1	1
	50	w				34	1	0	1	1	1
	50	w				45	1	0	0	0	1
	50	w				47	1	0	1	1	1
265.	53	w	27	3	2	26	1	0	0	1	1
	53	w				27	1	0	0	1	0
	53	w				35	0	1	1	99	99
	53	w				44	1	1	1	1	1
269.	55	w	16	2	1	22	1	0	0	2	1
	55	w				26	1	1	0	1	1
271.	22	m	28	1	0	21	1	0	1	1	0
272.	63	w	15	5	3	13	1	99	99	1	99
	63	w				11	1	99	1	0	1
	63	w				21	1	1	1	1	1
	63	w				35	1	1	1	1	1
	63	w				45	1	1	1	2	1
277.	41	w	26	1	0	11	1	0	0	0	0
278.	50	m	26	7	7	16	1	1	1	1	1
	50	m				12	1	1	0	0	1
	50	m				11	1	1	0	1	1
	50	m				22	1	1	1	1	1
	50	m				26	1	1	1	1	1
	50	m				36	1	1	0	1	1
	50	m				46	1	1	0	1	1
285.	52	w	24	6	3	15	1	0	1	0	1
	52	w				16	1	0	1	1	1
	52	w				11	1	1	1	99	1
	52	w				21	1	1	1	1	99
	52	w				46	1	0	1	1	1
	52	w				47	1	1	0	1	1
291.	24	m	26	4	0	12	1	0	1	1	1
	24	m				11	1	0	0	0	0
	24	m				22	1	0	0	0	1
	24	m				26	1	0	1	1	2
295.	60	m	22	1	1	26	1	1	1	1	1

296.	61	w	26	1	0	36	1	0	0	1	1
297.	70	w	26	7	4	12	1	1	0	1	1
	70	w				11	1	0	1	1	0
	70	w				25	1	0	1	1	1
	70	w				36	0	1	1	99	99
	70	w				37	1	0	1	1	1
	70	w				31	1	1	1	0	0
	70	w				46	1	1	0	1	1
	70	w				47	1	0	0	1	1
305.	56	w	14	2	0	13	1	0	0	1	1
	56	w				23	1	0	0	0	2
307.	21	m	28	0	1	12	0	1	1	99	99
308.	42	w	27	1	0	35	1	0	0	0	1
309.	37	m	26	2	0	26	1	0	0	1	0
	37	m				35	1	0	0	1	0
311.	46	m	28	2	1	16	1	99	1	1	0
	46	m				36	1	1	0	0	1
313.	31	w	23	2	0	26	1	0	0	1	1
	31	w				45	1	0	0	0	1
315.	51	w	14	7	1	13	1	0	1	1	1
	51	w				12	1	0	1	0	0
	51	w				21	1	0	1	0	0
	51	w				22	1	0	1	0	0
	51	w				23	1	0	1	0	1
	51	w				36	1	0	1	1	1
	51	w				34	0	1	1	99	99
	51	w				45	1	0	1	1	1
323.	53	m	22	2	1	24	1	1	99	1	1
	53	m				26	1	0	0	1	1
325.	51	w	28	2	2	16	1	1	0	0	1
	51	w				25	1	1	0	1	1
327.	39	m	22	5	3	22	1	1	0	0	1
	39	m				25	1	1	0	0	1
	39	m				27	1	1	0	0	1
	39	m				36	1	0	0	1	1
	39	m				44	1	0	0	0	0
332.	46	w	23	2	0	17	1	0	0	1	1
	46	w				26	1	0	0	1	1
334.	50	m	26	1	2	24	1	1	1	1	1
	50	m				36	0	1	1	99	99
336.	53	w	27	2	2	22	1	1	0	0	0
	53	w				26	0	1	0	99	99
	53	w				46	1	0	0	1	1
339.	31	w	28	2	1	46	1	0	0	1	0
	31	w				47	1	1	0	1	1
341.	42	w	15	1	0	15	1	0	0	1	1
342.	62	w	22	11	5	16	1	99	1	1	1
	62	w				14	1	1	1	1	1
	62	w				13	1	1	0	0	1
	62	w				12	1	1	99	1	99
	62	w				22	1	99	99	1	1
	62	w				23	1	0	0	1	0
	62	w				35	1	0	1	1	1
	62	w				34	1	0	0	1	1
	62	w				42	1	1	1	1	1
	62	w				44	1	0	0	1	1
	62	w				45	1	1	0	1	2
353.	54	w	23	7	4	16	1	1	1	1	1
	54	w				15	1	0	0	0	1
	54	w				14	1	1	0	1	1
	54	w				23	1	0	1	1	1
	54	w				26	1	1	0	1	1
	54	w				32	1	0	0	0	1
	54	w				41	0	1	1	99	99
	54	w				42	1	99	1	1	99
361.	70	w	22	2	4	17	0	1	1	99	99
	70	w				25	0	1	1	99	99
	70	w				37	0	1	1	99	99
	70	w				46	1	0	0	1	1
	70	w				47	1	1	0	1	1
366.	42	w	28	1	0	25	1	0	0	0	1
367.	59	w	20	5	1	15	1	0	0	1	1
	59	w				13	1	0	0	1	1
	59	w				12	1	99	99	0	1
	59	w				25	1	1	0	99	99
	59	w				37	1	0	1	1	1
372.	58	w	28	2	1	21	1	99	0	0	1

	58	w				46	1	1	0	0	1
374.	56	m	23	8	8	15	1	1	0	0	0
	56	m				12	0	1	1	99	99
	56	m				24	1	99	1	1	99
	56	m				25	1	1	0	1	1
	56	m				36	1	1	1	1	1
	56	m				35	1	1	0	0	0
	56	m				33	1	1	0	1	1
	56	m				45	1	1	0	1	1
	56	m				47	1	1	0	1	1
383.	60	w	17	3	1	16	1	1	1	2	1
	60	w				14	1	0	1	1	1
	60	w				22	1	0	1	0	1
386.	24	m	28	2	1	16	1	99	1	99	99
	24	m				15	1	1	1	1	99
388.	25	w	28	5	5	16	1	1	99	1	1
	25	w				15	1	1	0	0	1
	25	w				27	1	1	99	1	1
	25	w				37	1	1	0	1	1
	25	w				36	1	1	0	0	1
393.	60	m	27	5	2	17	1	0	0	1	1
	60	m				15	1	1	0	0	1
	60	m				14	1	0	1	0	1
	60	m				24	1	1	1	2	1
	60	m				23	1	0	1	0	1
398.	25	w	28	1	0	21	1	0	0	1	2
399.	61	w	25	8	6	15	1	0	0	1	1
	61	w				13	1	1	0	1	0
	61	w				12	1	0	0	0	0
	61	w				24	1	1	0	1	1
	61	w				25	1	1	0	1	1
	61	w				34	1	1	0	1	2
	61	w				45	1	1	1	1	1
	61	w				47	1	1	0	1	1
407.	55	w	23	10	5	17	1	1	99	1	1
	55	w				15	1	0	1	1	1
	55	w				27	1	0	1	1	1
	55	w				36	1	1	1	1	1
	55	w				35	1	0	1	1	1
	55	w				34	1	0	1	1	1
	55	w				44	1	99	1	1	1
	55	w				45	1	1	0	1	1
	55	w				46	1	1	99	99	99
	55	w				47	1	1	0	1	99
417.	41	m	28	7	5	17	1	1	1	1	1
	41	m				25	1	1	0	1	2
	41	m				26	1	99	1	1	1
	41	m				36	1	1	0	1	1
	41	m				35	1	0	1	0	1
	41	m				46	1	1	0	1	1
	41	m				47	1	1	0	1	1
424.	66	w	8	1	0	34	1	0	0	2	1
425.	55	w	19	4	0	24	1	0	0	1	1
	55	w				36	1	0	0	1	1
	55	w				34	1	0	1	0	0
	55	w				47	1	0	0	1	1
429.	50	m	24	6	4	14	1	1	1	1	1
	50	m				23	0	1	1	99	99
	50	m				24	1	0	0	0	1
	50	m				25	1	0	1	0	1
	50	m				26	1	1	99	1	0
	50	m				35	1	0	1	1	1
	50	m				47	1	1	0	1	1
436.	57	m	20	1	0	47	1	0	0	0	0
437.	29	w	22	0	1	22	0	1	0	99	99
438.	52	m	22	2	1	15	1	1	99	1	0
	52	m				45	1	0	1	1	1
440.	59	w	27	6	3	16	1	1	0	1	1
	59	w				24	1	1	0	1	0
	59	w				25	1	0	0	1	1
	59	w				45	1	0	1	1	1
	59	w				46	1	0	1	1	1
	59	w				47	1	1	0	0	1
446.	60	w	15	5	3	17	1	1	0	1	1
	60	w				14	1	0	1	1	1
	60	w				23	1	1	0	0	1
	60	w				32	0	1	1	99	99

	60	w				26	1	0	0	1	1
	60	w				41	1	99	99	99	99
452.	53	w	15	3	3	21	1	99	1	1	99
	53	w				14	0	1	1	99	99
	53	w				22	1	99	99	99	99
	53	w				33	0	1	99	99	99
	53	w				47	1	1	0	0	0
443.	49	w	28	0	1	17	0	1	1	99	99
457.	55	w	18	1	2	37	0	1	1	99	99
	55	w				23	1	1	0	1	0
459.	60	m	18	4	1	12	1	0	0	1	1
	60	m				11	1	1	99	1	0
	60	m				24	1	0	1	1	1
	60	m				25	1	0	0	1	1
463.	53	w	28	4	1	17	1	0	1	1	1
	53	w				14	1	1	0	1	0
	53	w				37	1	0	0	1	1
	53	w				45	1	0	0	0	1
467.	51	w	26	6	3	16	1	99	1	1	1
	51	w				22	1	1	1	0	1
	51	w				35	1	0	0	0	1
	51	w				44	1	1	0	1	1
	51	w				45	1	0	0	0	1
	51	w				46	1	1	0	0	0
473.	77	m	12	2	1	36	0	1	0	99	99
	77	m				44	1	0	1	1	1
	77	m				45	1	0	0	1	1
476.	71	m	17	2	2	23	1	1	0	1	0
	71	m				44	1	1	1	1	1
478.	67	m	12	4	4	35	1	1	0	1	1
	67	m				34	1	1	0	0	1
	67	m				44	1	0	0	1	1
	67	m				45	1	1	1	1	1
	67	m				47	0	1	0	99	99
483.	19	w	28	1	99	25	1	99	0	1	1
484.	76	m	16	3	1	21	1	1	0	0	2
	76	m				24	1	0	0	1	1
	76	m				36	1	0	0	0	1
487.	45	w	14	1	0	27	1	0	1	1	0
488.	46	m	26	6	6	17	1	99	1	1	1
	46	m				16	1	1	1	1	1
	46	m				24	1	1	0	1	1
	46	m				27	0	1	0	99	99
	46	m				36	1	1	0	1	1
	46	m				45	1	0	1	0	1
	46	m				46	1	1	0	1	1
	46	m				47	0	1	0	99	99
496.	33	m	27	3	1	17	1	0	1	1	1
	33	m				15	1	1	0	1	1
	33	m				24	1	99	1	1	1
499.	67	w	19	4	3	22	1	0	0	1	0
	67	w				23	1	1	0	0	1
	67	w				37	0	1	1	99	99
	67	w				44	1	0	1	1	0
	67	w				45	1	1	0	0	1
504.	64	m	26	5	3	16	1	1	0	1	0
	64	m				15	1	0	1	1	1
	64	m				25	0	1	1	99	99
	64	m				36	1	1	0	1	1
	64	m				35	1	0	1	1	1
	64	m				47	1	0	1	1	2
510.	33	w	27	1	5	14	0	1	0	99	99
	33	w				24	0	1	1	99	99
	33	w				25	0	1	1	99	99
	33	w				26	0	1	1	99	99
	33	w				36	1	1	1	1	1
515.	34	m	25	10	4	16	1	99	1	1	1
	34	m				15	1	1	1	1	1
	34	m				14	1	0	0	1	1
	34	m				12	1	1	0	1	0
	34	m				21	1	0	0	1	0
	34	m				24	1	0	0	1	1
	34	m				26	1	0	0	1	1
	34	m				37	1	1	1	1	1
	34	m				45	1	0	0	0	1
	34	m				47	1	1	0	1	1
525.	71	w	15	2	3	33	1	0	0	1	1

	71	w				35	0	1	1	99	99
	71	w				16	0	1	1	99	99
	71	w				44	1	1	1	1	1
529.	45	m	27	7	2	17	1	0	1	1	1
	45	m				15	1	0	0	99	1
	45	m				24	1	0	0	99	1
	45	m				26	1	0	1	99	1
	45	m				27	1	0	0	1	1
	45	m				36	1	1	0	1	2
	45	m				47	1	1	0	1	1
536.	61	w	10	2	0	16	1	99	99	1	1
	61	w				45	1	0	1	1	1
538.	62	m	14	4	0	12	1	0	1	2	1
	62	m				11	1	0	0	0	0
	62	m				22	1	0	1	1	1
	62	m				33	1	0	1	1	1
542.	60	w	15	5	3	15	1	1	0	0	1
	60	w				14	1	1	0	1	1
	60	w				23	1	1	0	1	0
	60	w				33	1	0	0	1	0
	60	w				43	1	0	0	1	2
547.	66	m	9	1	3	34	1	1	0	1	1
	66	m				45	0	1	1	99	99
	66	m				44	0	1	1	99	99
550.	51	m	27	4	4	17	1	1	0	1	1
	51	m				26	0	1	1	99	99
	51	m				27	1	1	99	1	1
	51	m				36	1	1	1	1	1
	51	m				35	1	0	0	1	99
555.	40	m	24	5	2	14	1	0	1	1	1
	40	m				22	1	1	0	1	0
	40	m				25	1	0	1	1	1
	40	m				37	1	1	0	1	1
	40	m				35	1	0	0	0	1
560.	69	w	26	8	0	17	1	0	1	1	1
	69	w				12	1	99	99	99	1
	69	w				22	1	99	99	99	1
	69	w				23	1	99	99	99	1
	69	w				25	1	99	99	99	1
	69	w				26	1	0	99	1	1
	69	w				35	1	0	0	1	1
	69	w				45	1	0	0	0	1
568.	63	w	19	3	1	14	1	99	1	1	1
	63	w				35	1	0	0	1	1
	63	w				33	1	1	0	1	2
571.	60	m	16	1	0	14	1	0	0	1	1
572.	45	m	26	5	1	21	1	0	1	0	0
	45	m				22	1	0	1	0	0
	45	m				25	1	0	1	0	1
	45	m				26	1	1	0	1	1
	45	m				45	1	0	0	0	0
577.	67	w	23	6	3	14	1	1	1	1	1
	67	w				12	1	1	99	99	99
	67	w				21	1	99	99	2	1
	67	w				22	1	1	99	2	1
	67	w				24	1	99	99	99	1
	67	w				26	1	0	99	1	1
583.	68	m	18	12	3	15	1	0	0	1	1
	68	m				14	1	0	1	1	1
	68	m				24	1	99	99	1	1
	68	m				25	1	99	99	1	1
	68	m				26	1	1	99	1	99
	68	m				37	1	0	0	1	1
	68	m				36	1	0	0	1	1
	68	m				35	1	0	0	0	1
	68	m				34	1	0	0	1	1
	68	m				44	1	1	0	1	1
	68	m				45	1	0	0	1	1
	68	m				47	1	1	99	1	1
595.	55	w	18	7	3	16	1	1	99	1	1
	55	w				11	1	1	99	0	0
	55	w				22	1	1	1	0	1
	55	w				23	1	0	1	1	1
	55	w				24	1	0	0	0	2
	55	w				35	1	0	1	1	1
	55	w				45	1	0	0	0	1
602.	56	w	23	2	1	23	1	99	99	1	99

	56	w				35	1	1	1	0	1
604.	54	m	24	2	1	14	1	0	0	1	1
	54	m				24	0	1	1	99	99
	54	m				12	1	0	0	1	99
607.	70	w	27	3	0	16	1	99	99	1	1
	70	w				12	1	0	0	0	1
	70	w				24	1	0	0	1	1
610.	70	w	25	2	1	37	1	1	0	1	1
	70	w				47	1	0	0	1	0
612.	55	m	23	2	0	37	1	0	1	1	1
	55	m				31	1	0	0	0	1
614.	67	w	8	2	0	31	1	0	1	0	1
	67	w				44	1	0	0	1	1
616.	55	m	21	1	1	26	1	0	1	0	1
	55	m				45	0	1	1	99	99
618.	42	m	23	6	4	17	1	0	0	1	99
	42	m				14	1	1	99	1	1
	42	m				21	1	99	99	1	1
	42	m				26	1	1	99	1	1
	42	m				35	1	1	1	0	0
	42	m				31	1	1	0	1	0
624.	45	m	28	1	0	36	1	0	0	1	1
625.	67	m	5	3	2	37	1	1	0	1	1
	67	m				44	1	0	1	1	1
	67	m				46	1	1	0	1	0
628.	74	w	8	1	0	21	1	0	0	2	1
629.	46	m	28	3	2	17	1	99	1	1	99
	46	m				15	1	1	0	1	1
	46	m				34	1	1	99	1	0
632.	39	m	23	2	0	15	1	99	1	0	1
	39	m				14	1	0	99	1	99
634.	54	m	24	1	1	37	1	1	1	0	1
635.	57	w	23	1	1	26	1	1	1	1	1
636.	72	w	19	4	0	12	1	0	1	0	0
	72	w				22	1	0	1	0	0
	72	w				26	1	0	0	1	1
	72	w				45	1	0	1	1	1
640.	71	w	7	0	1	44	0	1	1	99	99
641.	41	w	25	11	4	16	1	0	0	0	2
	41	w				15	1	1	1	1	2
	41	w				14	1	0	1	1	0
	41	w				22	1	0	1	1	0
	41	w				23	1	0	0	1	0
	41	w				24	1	1	0	1	1
	41	w				26	1	0	0	0	99
	41	w				27	0	1	1	99	99
	41	w				37	1	0	0	1	1
	41	w				35	1	1	0	1	0
	41	w				45	1	0	0	1	0
	41	w				46	1	0	0	0	0
653.	44	w	25	2	1	46	1	1	0	1	0
	44	w				47	1	0	0	0	1
655.	61	w	16	2	0	14	1	0	1	1	1
	61	w				35	1	0	0	1	0
657.	37	m	28	1	2	36	0	1	0	99	99
	37	m				24	0	1	1	99	99
	37	m				46	1	0	0	1	1
660.	49	w	28	1	1	46	1	1	1	1	1
661.	55	m	25	1	1	16	1	1	0	1	1
662.	56	w	9	7	1	35	1	0	1	0	0
	56	w				34	1	0	1	0	1
	56	w				33	1	0	0	1	0
	56	w				32	1	0	1	1	1
	56	w				31	1	0	0	0	1
	56	w				41	1	0	0	0	1
	56	w				42	0	1	0	99	99
	56	w				44	1	0	1	1	1
670.	63	m	20	4	4	14	1	0	0	0	1
	63	m				37	1	0	0	1	1
	63	m				34	1	1	0	0	0
	63	m				42	0	1	0	99	99
	63	m				43	0	1	0	99	99
	63	m				45	1	1	0	0	0
676.	56	w	13	0	2	45	0	1	0	99	99
	56	w				24	0	1	0	99	99
678.	48	m	26	4	0	11	1	0	1	1	0
	48	m				21	1	0	1	1	0

	48	m				24	1	0	1	0	99
	48	m				25	1	0	1	1	99
682.	60	w	18	5	2	16	1	0	1	1	1
	60	w				15	1	0	1	0	1
	60	w				12	1	1	99	1	99
	60	w				22	1	0	99	1	1
	60	w				25	1	0	1	1	1
	60	w				35	0	1	0	99	99
688.	54	w	28	4	3	26	1	1	0	0	0
	54	w				16	0	1	0	99	99
	54	w				36	1	0	0	0	1
	54	w				35	1	1	0	1	1
	54	w				46	1	0	0	0	1
693.	40	m	28	2	2	12	1	1	0	1	0
	40	m				11	1	1	0	0	1
695.	64	w	5	1	1	23	1	1	0	1	0
696.	54	w	25	3	0	16	1	0	0	1	1
	54	w				15	1	0	1	0	1
	54	w				25	0	0	1	99	99
	54	w				27	1	0	0	1	1
700.	72	w	12	9	3	21	1	0	1	1	0
	72	w				22	1	0	0	1	1
	72	w				23	1	1	1	1	1
	72	w				35	1	0	1	0	1
	72	w				32	1	1	0	0	0
	72	w				31	1	0	0	0	0
	72	w				41	1	1	0	0	0
	72	w				42	1	0	0	0	0
	72	w				45	1	0	0	1	1
709.	57	w	17	6	1	16	1	1	0	1	1
	57	w				11	1	99	99	99	1
	57	w				21	1	99	99	99	1
	57	w				22	1	99	99	99	1
	57	w				34	1	0	0	1	1
	57	w				45	1	0	0	0	1
715.	67	m	28	1	1	21	1	1	0	0	2
716.	46	w	26	4	0	21	1	99	1	1	99
	46	w				26	1	0	0	1	1
	46	w				27	1	0	1	1	1
	46	w				37	1	0	1	0	1
720.	52	w	24	7	2	17	1	0	1	1	1
	52	w				21	1	0	0	1	1
	52	w				22	1	1	0	1	1
	52	w				24	1	0	0	1	1
	52	w				36	1	1	0	1	1
	52	w				35	1	0	0	0	0
	52	w				43	1	0	0	0	0
727.	64	w	22	9	4	15	1	0	0	0	1
	64	w				14	1	0	0	1	1
	64	w				13	1	1	0	1	1
	64	w				23	1	0	1	0	0
	64	w				24	1	0	0	1	1
	64	w				26	1	1	0	1	1
	64	w				27	1	99	1	1	0
	64	w				46	1	1	0	1	1
	64	w				47	1	1	0	1	1
736.	59	w	24	3	3	17	1	1	99	1	1
	59	w				21	1	0	1	1	1
	59	w				24	0	1	1	99	99
	59	w				27	0	0	1	99	99
	59	w				37	0	1	1	99	99
	59	w				26	1	0	1	1	1
742.	59	w	22	4	1	17	1	0	1	1	1
	59	w				16	1	1	0	1	1
	59	w				35	1	0	0	0	1
	59	w				45	1	0	0	1	1
746.	53	w	21	2	1	37	1	0	1	1	0
	53	w				36	1	1	1	1	1
748.	73	w	5	3	1	35	1	1	0	1	1
	73	w				34	1	0	0	1	1
	73	w				43	1	0	0	1	1
751.	54	m	21	8	2	17	1	0	1	1	1
	54	m				11	1	0	1	1	1
	54	m				21	1	0	1	1	1
	54	m				22	1	1	0	0	0
	54	m				23	1	0	0	0	0
	54	m				24	1	0	0	0	1

	54	m				25	0	1	1	99	99
	54	m				26	1	0	0	1	1
	54	m				45	1	0	0	1	1
760.	83	w	4	4	2	33	1	0	1	1	1
	83	w				32	1	1	0	1	1
	83	w				42	1	1	0	1	0
	83	w				43	1	0	0	1	0
764.	46	w	28	3	1	17	1	0	0	0	0
	46	w				16	1	99	0	1	1
	46	w				45	1	1	0	1	0
767.	42	w	26	1	0	47	1	0	0	1	1
768.	50	m	27	9	4	17	1	1	0	0	1
	50	m				16	1	0	1	1	1
	50	m				15	1	0	1	0	1
	50	m				25	1	0	0	2	1
	50	m				26	1	0	1	0	0
	50	m				37	1	0	1	0	1
	50	m				36	1	1	0	1	1
	50	m				46	1	1	1	0	1
	50	m				47	1	1	1	1	1
777.	60	m	21	5	1	17	1	0	0	1	1
	60	m				16	0	1	0	99	99
	60	m				15	1	0	1	0	1
	60	m				27	1	0	0	1	1
	60	m				45	1	0	0	0	2
	60	m				47	1	0	1	1	1
783.	68	m	19	4	1	14	1	0	0	1	0
	68	m				13	1	0	0	1	0
	68	m				12	1	0	0	1	0
	68	m				43	1	1	99	99	2
787.	70	m	20	3	3	13	1	0	1	0	1
	70	m				21	0	1	0	99	99
	70	m				23	1	99	1	0	2
	70	m				25	1	1	99	1	1
	70	m				26	0	1	1	99	99
792.	73	m	11	2	0	12	1	0	1	0	0
	73	m				23	1	0	0	0	0
794.	35	w	26	5	1	15	1	0	0	1	1
	35	w				13	1	99	99	1	99
	35	w				36	1	0	0	1	1
	35	w				46	1	0	1	1	1
	35	w				47	1	1	0	1	1
799.	77	w	20	1	2	14	0	1	1	99	99
	77	w				15	0	1	0	99	99
	77	w				35	1	0	0	0	1
802.	60	w	18	9	2	13	1	0	1	1	0
	60	w				16	0	1	1	99	99
	60	w				12	1	0	0	1	0
	60	w				11	1	0	0	1	1
	60	w				21	1	0	1	1	0
	60	w				22	1	0	0	1	0
	60	w				23	1	0	1	1	0
	60	w				24	1	1	0	1	0
	60	w				25	1	0	0	0	1
	60	w				43	1	0	1	0	1
812.	61	m	23	9	2	16	1	0	0	0	1
	61	m				11	1	0	0	1	1
	61	m				21	1	0	0	1	0
	61	m				22	1	0	1	1	0
	61	m				24	1	0	0	1	1
	61	m				25	1	0	1	1	0
	61	m				26	1	1	0	1	1
	61	m				34	1	1	0	0	1
	61	m				45	1	0	0	1	0
821.	58	w	7	0	3	33	0	1	1	99	99
	58	w				34	0	1	1	99	99
	58	w				47	0	1	1	99	99
824.	35	m	28	1	1	46	1	1	1	0	1
825.	27	m	28	1	1	35	1	1	0	1	1
826.	53	w	22	2	0	24	1	0	0	1	1
	53	w				27	1	0	1	0	1
828.	59	w	26	2	0	25	1	0	1	0	0
	59	w				34	1	0	0	0	0
830.	51	m	24	2	3	27	1	1	1	1	1
	51	m				14	0	1	0	99	99
	51	m				46	1	1	0	1	1
833.	70	w	19	2	2	12	1	1	0	1	2

	70	w				17	0	1	1	99	99
	70	w				34	1	0	0	0	0
836.	81	w	7	6	1	12	1	0	0	0	1
	81	w				11	1	0	0	0	0
	81	w				21	1	99	1	1	1
	81	w				22	1	99	99	99	99
	81	w				35	1	1	1	1	1
	81	w				34	1	99	1	0	1
842.	57	w	26	5	5	16	1	1	99	1	1
	57	w				15	1	1	0	1	1
	57	w				14	1	1	99	1	1
	57	w				13	1	1	1	1	1
	57	w				22	0	0	1	99	99
	57	w				26	1	1	99	1	1
848.	44	m	27	12	3	15	1	0	0	0	1
	44	m				14	1	0	1	1	1
	44	m				12	1	99	1	1	1
	44	m				11	1	1	0	1	1
	44	m				22	1	0	0	1	0
	44	m				24	1	0	1	1	1
	44	m				25	1	0	1	0	0
	44	m				26	1	1	0	1	1
	44	m				27	1	99	99	1	1
	44	m				36	1	0	0	1	1
	44	m				44	1	1	0	1	1
	44	m				46	1	0	1	1	1
860.	55	w	26	2	1	24	1	0	1	2	1
	55	w				12	0	1	0	99	99
	55	w				47	1	0	0	1	1
863.	51	w	27	2	1	12	1	0	0	0	1
	51	w				22	1	1	0	0	1
865.	50	m	26	8	3	17	1	0	0	0	1
	50	m				16	1	1	0	1	1
	50	m				15	1	0	0	1	1
	50	m				14	1	0	0	1	1
	50	m				24	1	0	0	1	1
	50	m				27	1	1	1	1	1
	50	m				37	1	1	1	1	1
	50	m				46	1	0	0	0	1
873.	63	m	24	1	0	45	1	0	0	0	0
874.	49	w	18	9	4	12	1	0	0	99	1
	49	w				11	1	1	1	1	0
	49	w				21	1	1	1	1	1
	49	w				23	1	0	1	1	0
	49	w				24	1	1	99	2	1
	49	w				33	1	0	1	1	0
	49	w				32	1	1	0	1	1
	49	w				44	1	0	0	0	1
	49	w				46	1	0	0	1	1
883.	59	w	10	1	6	15	1	0	1	0	0
	59	w				37	0	1	0	99	99
	59	w				35	0	1	0	99	99
	59	w				34	0	1	0	99	99
	59	w				33	0	1	0	99	99
	59	w				43	0	1	0	99	99
	59	w				44	0	1	0	99	99
890.	53	w	27	8	6	17	1	1	99	1	1
	53	w				16	1	1	99	1	1
	53	w				15	1	99	1	1	1
	53	w				14	1	1	0	0	1
	53	w				11	1	0	0	0	1
	53	w				21	1	1	0	2	1
	53	w				25	1	1	0	0	1
	53	w				36	1	1	0	0	0
898.	48	w	28	2	2	36	1	1	0	1	1
	48	w				26	1	1	0	1	1
900.	47	w	28	1	0	46	1	0	1	0	1
901.	59	m	26	1	0	26	1	0	0	1	1
902.	63	m	22	1	0	12	1	0	1	1	1
903.	67	m	16	3	2	25	1	0	0	1	0
	67	m				43	1	1	0	1	1
	67	m				33	1	1	0	1	1
906.	52	w	27	2	1	15	1	0	0	0	0
	52	w				37	1	1	0	1	1
908.	76	m	5	0	3	44	0	1	0	99	99
	76	m				13	0	1	1	99	99
	76	m				12	0	1	1	99	99

911.	43	m	27	4	3	15	1	1	1	0	0
	43	m				37	1	0	0	0	0
	43	m				36	1	1	0	1	0
	43	m				47	1	1	1	1	0
915.	43	w	28	4	1	16	1	0	1	1	1
	43	w				25	1	0	1	1	1
	43	w				36	1	0	1	1	1
	43	w				35	1	1	0	0	1
919.	47	m	24	5	3	15	1	1	0	1	99
	47	m				27	1	0	99	1	0
	47	m				34	1	1	1	0	0
	47	m				44	1	0	1	1	0
	47	m				46	1	1	99	1	1
924.	61	w	12	2	0	13	1	0	1	1	1
	61	w				12	1	0	1	0	0
926.	63	m	16	3	0	11	1	0	1	1	0
	63	m				21	1	0	1	0	0
	63	m				22	1	99	1	1	99
929.	52	w	19	2	1	13	1	1	0	0	0
	52	w				23	1	0	0	1	99
931.	55	w	28	5	2	16	1	0	0	1	99
	55	w				15	1	0	0	1	0
	55	w				21	1	1	0	1	2
	55	w				27	1	99	1	1	1
	55	w				46	1	1	0	1	1
936.	44	m	24	2	1	15	1	0	1	1	1
	44	m				25	1	1	0	1	1
938.	79	m	8	1	0	34	1	0	99	99	99
939.	75	w	5	1	0	45	1	0	0	1	0
940.	50	w	25	7	4	17	1	0	0	1	1
	50	w				13	1	0	99	1	1
	50	w				12	1	1	1	1	1
	50	w				22	1	1	1	1	1
	50	w				24	1	1	0	1	1
	50	w				26	1	1	0	1	1
	50	w				27	1	0	0	1	1
947.	53	m	28	3	1	14	1	0	0	1	1
	53	m				25	1	1	0	0	1
	53	m				26	1	0	0	1	0
950.	21	m	28	1	1	46	1	0	0	1	2
	21	m				26	0	1	0	99	99
952.	56	w	24	6	4	23	1	0	1	1	1
	56	w				27	1	1	0	1	99
	56	w				36	1	1	1	2	1
	56	w				31	1	99	99	1	99
	56	w				46	1	1	1	1	1
	56	w				47	1	1	1	1	1
958.	50	m	21	7	2	17	1	1	99	1	1
	50	m				16	1	1	99	1	1
	50	m				15	1	0	0	1	0
	50	m				12	1	0	1	0	1
	50	m				24	1	99	1	1	1
	50	m				25	1	0	0	1	1
	50	m				26	1	0	1	99	1
965.	47	w	21	0	3	17	0	1	1	99	99
	47	w				23	0	1	1	99	99
	47	w				46	0	1	0	99	99
968.	54	w	18	1	0	12	1	0	0	1	1
969.	43	w	24	7	2	15	1	0	0	1	1
	43	w				14	1	0	1	1	1
	43	w				24	1	1	99	1	99
	43	w				25	1	0	1	1	99
	43	w				26	1	1	0	1	1
	43	w				27	1	0	1	1	1
	43	w				37	1	0	1	0	0
976.	44	m	17	7	5	17	1	1	0	1	1
	44	m				16	1	1	0	1	1
	44	m				15	1	1	0	0	0
	44	m				14	1	99	1	1	1
	44	m				22	0	1	0	99	99
	44	m				26	1	0	0	1	1
	44	m				27	1	1	0	1	1
	44	m				36	1	0	1	1	1
984.	44	w	20	8	5	16	1	1	0	1	1
	44	w				17	0	1	0	99	99
	44	w				15	1	1	0	1	1
	44	w				13	1	1	0	0	99

	44	w				12	1	0	0	0	99
	44	w				22	1	0	0	0	0
	44	w				23	1	0	1	1	1
	44	w				26	1	1	99	1	1
	44	w				44	1	0	0	0	1
993.	57	w	16	1	0	14	1	0	1	1	1
994.	65	m	8	4	1	15	1	1	0	1	0
	65	m				13	1	0	0	1	0
	65	m				23	1	0	1	1	1
	65	m				24	1	0	0	1	1
998.	78	w	8	2	5	34	0	1	0	99	99
	78	w				33	0	1	1	99	99
	78	w				31	0	1	1	99	99
	78	w				41	1	1	99	1	0
	78	w				42	1	1	99	1	0
1003.	59	m	19	2	0	23	1	0	0	1	0
	59	m				35	1	0	1	1	1
1005.	59	w	17	4	1	21	1	0	0	1	1
	59	w				37	1	0	1	1	1
	59	w				31	1	0	1	1	1
	59	w				45	1	1	0	1	1
1009.	67	w	24	1	99	15	1	99	1	1	1
1010.	66	m	17	7	3	16	1	0	0	1	1
	66	m				15	1	0	1	1	1
	66	m				27	1	0	0	1	1
	66	m				33	1	1	1	0	0
	66	m				41	1	1	0	1	1
	66	m				42	1	1	99	1	1
	66	m				46	1	0	0	2	1
1017.	38	w	28	2	2	16	1	1	99	1	1
	38	w				36	1	1	0	1	1
1019.	89	w	4	2	1	43	1	1	0	0	0
	89	w				44	1	0	0	0	0
1021.	51	m	19	3	2	13	1	1	0	1	1
	51	m				24	1	99	99	1	1
	51	m				36	1	1	1	1	2
1024.	25	w	28	4	3	17	1	1	99	1	0
	25	w				15	1	99	99	1	99
	25	w				25	1	1	99	1	99
	25	w				26	1	1	1	1	2
1028.	77	w	28	3	1	26	1	0	1	1	1
	77	w				36	1	1	0	1	1
	77	w				45	1	0	0	0	1
1031.	64	w	24	1	1	21	1	1	0	0	0
1032.	57	w	22	0	1	36	0	1	0	99	99
1033.	47	w	26	4	1	22	1	0	0	0	0
	47	w				25	1	1	99	1	1
	47	w				45	1	0	1	0	1
	47	w				46	1	0	0	1	1
1037.	62	w	19	7	0	15	1	0	1	1	1
	62	w				14	1	0	1	1	1
	62	w				12	1	0	1	1	2
	62	w				36	1	0	1	1	1
	62	w				35	1	0	0	1	1
	62	w				34	1	0	0	1	1
	62	w				44	1	0	0	0	1
1044.	31	w	27	1	1	35	1	1	0	0	0
1045.	63	m	26	7	1	15	1	0	1	0	1
	63	m				21	1	0	1	1	0
	63	m				24	1	0	0	1	1
	63	m				26	1	0	0	2	1
	63	m				37	1	1	0	1	1
	63	m				44	1	0	0	2	1
	63	m				45	1	0	0	2	1
1052.	59	m	27	2	0	15	1	0	1	2	1
	59	m				12	1	99	99	99	0
1054.	68	w	15	3	0	14	1	0	1	1	1
	68	w				13	1	99	0	1	1
	68	w				26	1	0	1	1	1
1057.	36	w	27	1	0	15	1	0	1	0	1
1058.	69	w	16	5	2	15	1	1	0	1	0
	69	w				13	1	0	1	1	1
	69	w				22	1	0	0	0	1
	69	w				25	1	0	1	0	1
	69	w				33	1	0	0	1	1
	69	w				44	0	1	0	99	99
1064.	64	m	23	5	1	23	1	0	1	1	0

	64	m				24	1	0	0	1	0
	64	m				37	1	0	0	1	1
	64	m				36	1	1	0	1	0
	64	m				35	1	0	0	1	1
1069.	39	w	26	5	1	12	1	1	0	99	99
	39	w				21	1	0	1	0	1
	39	w				22	1	0	0	1	99
	39	w				26	1	0	1	1	1
	39	w				35	1	0	1	0	1
1074.	73	w	24	1	0	16	1	0	1	2	1
1075.	44	m	14	4	1	37	1	0	1	1	1
	44	m				35	1	0	1	0	1
	44	m				45	1	0	0	1	0
	44	m				46	1	1	1	1	1
1079.	52	w	25	4	1	24	1	0	1	1	1
	52	w				35	1	0	0	0	0
	52	w				32	1	0	0	0	1
	52	w				45	1	1	0	1	1
1083.	66	m	25	4	3	14	1	1	99	1	1
	66	m				26	1	1	0	0	1
	66	m				36	1	1	1	1	1
	66	m				34	1	0	0	0	1
1087.	49	m	22	12	1	15	1	0	0	1	1
	49	m				14	1	0	0	0	1
	49	m				12	1	99	99	2	1
	49	m				11	1	0	0	0	0
	49	m				23	1	1	1	1	0
	49	m				24	1	0	0	1	0
	49	m				26	1	0	0	1	1
	49	m				35	1	0	0	1	1
	49	m				34	1	0	0	1	1
	49	m				44	1	0	0	1	1
	49	m				45	1	0	1	1	1
	49	m				46	1	0	1	1	1
1099.	74	m	24	8	4	16	1	1	1	1	1
	74	m				14	1	99	1	99	1
	74	m				12	1	99	99	1	0
	74	m				21	1	1	0	1	2
	74	m				22	1	0	0	1	1
	74	m				23	1	1	0	1	99
	74	m				24	0	1	1	99	99
	74	m				46	1	0	0	0	1
	74	m				47	1	0	0	2	1
1108.	44	w	27	1	2	15	1	0	1	1	1
	44	w				14	0	1	1	99	99
	44	w				26	0	1	0	99	99
1111.	44	m	28	0	1	47	0	1	0	99	99
1112.	46	m	28	2	1	17	1	1	1	1	1
	46	m				45	1	0	1	0	1
1114.	75	w	10	3	1	13	1	0	1	1	1
	75	w				12	1	1	0	1	1
	75	w				31	1	0	1	1	1
1117.	69	w	23	1	0	45	1	0	0	0	1
1118.	55	w	23	3	1	26	1	1	1	1	1
	55	w				35	1	0	0	0	1
	55	w				46	1	0	1	1	1
1121.	73	w	27	6	2	16	1	99	99	99	99
	73	w				11	1	0	99	0	1
	73	w				21	1	1	99	0	0
	73	w				25	1	1	0	1	0
	73	w				27	1	0	0	1	1
	73	w				47	1	0	0	1	1
1127.	57	w	20	4	0	16	1	0	1	1	1
	57	w				24	1	0	0	1	1
	57	w				34	1	0	1	1	0
	57	w				44	1	0	1	1	1
1131.	52	m	24	3	2	15	1	1	0	1	0
	52	m				27	1	1	1	0	2
	52	m				35	1	0	0	0	1
1134.	47	m	17	4	2	14	1	0	0	1	1
	47	m				12	1	1	0	1	1
	47	m				26	1	1	1	1	1
	47	m				41	1	0	1	1	2
1138.	33	w	28	1	0	36	1	0	1	1	1
1139.	47	w	27	1	1	36	1	1	0	1	1
1140.	73	m	17	7	4	17	1	1	1	1	2
	73	m				14	1	1	1	0	1

	73	m				26	1	1	1	0	2
	73	m				35	1	0	0	0	0
	73	m				32	1	1	0	1	0
	73	m				41	1	0	0	1	1
	73	m				45	1	0	1	1	2
1147.	31	w	26	4	1	17	1	0	0	2	1
	31	w				16	1	1	0	1	1
	31	w				15	1	0	1	1	1
	31	w				47	1	0	0	0	0
1151.	61	m	17	7	2	16	1	1	0	1	1
	61	m				11	1	0	1	1	1
	61	m				21	1	0	0	2	1
	61	m				23	1	1	0	1	0
	61	m				24	1	0	1	1	1
	61	m				26	1	0	1	2	1
	61	m				35	1	0	1	1	1
1158.	41	m	27	3	3	22	1	1	0	0	0
	41	m				26	1	1	0	0	1
	41	m				36	1	1	0	1	1
1161.	70	m	25	3	2	16	1	1	0	2	1
	70	m				15	1	0	0	1	1
	70	m				45	1	1	0	2	1
1164.	22	w	28	1	1	26	1	1	0	1	0
1165.	63	m	28	3	1	15	1	0	0	1	2
	63	m				26	1	0	1	1	1
	63	m				46	1	1	1	0	0
1168.	71	m	23	3	1	14	1	0	1	1	1
	71	m				13	1	0	0	0	0
	71	m				36	0	1	1	99	99
	71	m				25	1	0	0	1	0
1172.	67	w	27	5	0	15	1	0	0	1	1
	67	w				14	1	0	0	1	2
	67	w				13	1	0	1	1	1
	67	w				25	1	0	1	1	1
	67	w				46	1	0	0	1	1
1177.	44	w	25	1	0	24	1	0	0	0	1
1178.	50	m	28	1	1	15	1	1	0	1	1
1179.	59	w	19	3	0	17	1	0	0	0	2
	59	w				35	1	0	1	1	1
	59	w				34	1	0	0	0	1
1182.	51	w	24	1	0	36	1	0	0	1	1
1183.	77	m	14	1	1	23	1	1	0	0	0
1184.	66	w	22	13	1	14	1	0	0	1	1
	66	w				13	1	0	0	0	1
	66	w				12	1	0	0	0	1
	66	w				11	1	0	1	0	0
	66	w				21	1	0	0	0	0
	66	w				22	1	0	1	0	1
	66	w				23	1	0	0	0	1
	66	w				26	0	1	1	99	99
	66	w				37	1	0	1	2	1
	66	w				35	1	0	1	0	1
	66	w				34	1	0	0	1	1
	66	w				45	1	0	0	1	1
	66	w				46	1	0	0	1	1
	66	w				47	1	0	0	1	1
1198.	59	w	25	8	3	17	1	1	1	1	1
	59	w				14	1	0	1	1	1
	59	w				13	1	0	1	1	0
	59	w				12	1	0	0	0	0
	59	w				21	1	0	0	0	99
	59	w				22	1	0	1	0	1
	59	w				25	1	1	1	1	0
	59	w				47	1	1	0	1	1
1206.	55	w	28	2	1	14	1	0	1	1	1
	55	w				27	0	1	1	99	99
	55	w				24	1	0	99	1	0
1209.	53	w	17	9	3	16	1	0	1	1	1
	53	w				14	1	0	1	1	1
	53	w				13	1	0	0	1	0
	53	w				11	1	0	0	1	0
	53	w				21	1	0	1	0	1
	53	w				22	1	0	0	1	1
	53	w				26	1	1	99	1	1
	53	w				27	1	1	1	1	1
	53	w				42	1	1	1	1	1
1218.	55	w	17	8	3	12	1	0	0	1	1

	55	w				11	1	1	0	0	1
	55	w				22	1	0	1	0	0
	55	w				21	0	1	0	99	99
	55	w				37	1	0	1	1	1
	55	w				35	1	0	0	0	1
	55	w				33	1	0	0	0	1
	55	w				45	1	0	0	1	2
	55	w				47	1	1	0	1	1
1227.	45	m	26	2	0	17	1	0	1	1	1
	45	m				46	1	0	0	0	1
1229.	61	w	18	1	1	35	1	1	1	0	1
1230.	62	w	22	4	1	16	1	99	1	99	99
	62	w				14	1	1	99	1	1
	62	w				13	1	0	1	1	0
	62	w				26	1	0	0	1	1
1234.	26	m	28	1	0	24	1	0	0	1	1
1235.	40	m	26	2	1	15	1	0	0	1	2
	40	m				27	1	1	0	1	1
1237.	63	w	25	9	2	17	1	1	1	1	1
	63	w				16	1	99	1	1	1
	63	w				15	1	99	1	1	1
	63	w				12	1	1	1	1	1
	63	w				25	1	0	0	1	0
	63	w				27	1	0	1	0	1
	63	w				37	1	0	0	1	1
	63	w				44	1	0	0	0	1
	63	w				46	1	0	1	0	1
1246.	62	m	23	6	3	17	0	1	1	99	99
	62	m				16	1	0	0	0	1
	62	m				14	1	0	1	2	1
	62	m				26	1	0	1	1	1
	62	m				27	1	1	0	2	1
	62	m				37	1	0	0	1	0
	62	m				34	0	1	1	99	99
	62	m				47	1	0	0	1	1
1254.	68	m	12	0	1	14	0	1	1	99	99
1255.	58	m	27	4	3	17	1	0	1	1	2
	58	m				15	1	1	0	1	1
	58	m				12	1	1	0	1	0
	58	m				35	1	1	0	0	1
1259.	36	m	26	7	5	17	1	1	1	1	1
	36	m				15	1	1	0	0	1
	36	m				14	1	0	0	1	1
	36	m				26	1	0	0	0	1
	36	m				27	1	1	1	1	1
	36	m				36	1	1	0	1	1
	36	m				35	1	1	0	0	0
1266.	47	m	27	2	1	16	1	0	1	1	1
	47	m				24	1	1	0	1	1
1268.	46	m	25	3	1	15	1	0	1	0	1
	46	m				14	1	0	0	1	1
	46	m				27	1	1	1	1	1
1271.	71	m	26	3	2	35	1	1	1	1	1
	71	m				45	1	0	1	1	1
	71	m				46	1	1	1	1	1
1274.	75	w	15	5	2	21	1	99	1	99	1
	75	w				26	1	1	1	1	1
	75	w				27	1	1	1	1	1
	75	w				35	1	0	0	1	1
	75	w				44	1	0	1	1	0
1279.	73	m	18	5	4	14	1	1	99	1	1
	73	m				37	1	1	0	1	1
	73	m				35	1	0	0	1	1
	73	m				34	1	1	0	0	0
	73	m				46	1	1	1	1	1
1284.	51	m	26	1	1	47	1	1	0	1	1
1285.	38	w	27	5	3	17	1	1	1	1	1
	38	w				27	1	1	1	1	1
	38	w				36	1	0	1	1	1
	38	w				35	1	0	0	0	1
	38	w				45	1	1	0	1	0
1290.	70	w	18	3	1	26	1	0	0	1	1
	70	w				45	1	0	1	1	1
	70	w				46	1	1	0	1	1
1293.	26	w	28	1	0	21	1	0	0	1	0
1294.	49	m	25	4	3	15	1	1	0	0	0
	49	m				25	1	0	1	0	1

	49	m				26	1	99	1	1	1
	49	m				27	1	1	0	1	1
	49	m				36	0	1	1	99	99
1299.	52	w	21	5	2	11	1	0	1	1	1
	52	w				21	1	0	1	1	2
	52	w				37	1	0	0	0	0
	52	w				41	1	1	0	1	2
	52	w				46	1	1	1	1	1
1304.	67	w	28	6	1	16	1	1	1	2	1
	67	w				15	1	0	0	2	1
	67	w				25	1	0	0	1	1
	67	w				26	1	0	1	1	1
	67	w				37	1	0	0	0	1
	67	w				35	1	0	0	1	1
1310.	50	m	19	1	1	46	1	1	0	1	1
1311.	44	w	23	1	0	26	1	0	0	1	1
1312.	52	w	21	4	3	15	1	1	0	0	1
	52	w				24	1	0	0	1	1
	52	w				35	1	0	0	1	1
	52	w				34	1	1	1	1	1
	52	w				44	0	1	1	99	99
1317.	53	w	17	3	1	17	1	1	1	1	1
	53	w				11	1	0	0	0	1
	53	w				35	1	0	0	0	0
1320.	66	w	20	3	4	13	1	1	0	1	1
	66	w				17	0	1	1	99	99
	66	w				45	1	1	0	1	0
	66	w				46	1	1	0	1	1
1324.	63	m	20	5	1	17	1	0	1	1	1
	63	m				13	1	99	0	0	1
	63	m				24	1	1	0	1	1
	63	m				25	1	0	0	0	1
	63	m				27	1	0	0	1	1
1329.	74	m	21	1	0	15	1	0	1	1	1
1330.	43	m	27	3	2	16	1	1	1	1	1
	43	m				12	1	0	1	1	1
	43	m				36	1	1	1	1	1
3154.	76	m	4	1	1	23	1	1	99	99	1
1333.	74	m	21	1	1	14	1	0	0	0	1
	74	m				26	0	1	1	99	99
1335.	73	w	17	3	3	15	1	1	0	1	1
	73	w				14	1	1	1	2	1
	73	w				34	1	1	0	2	1
1338.	69	w	24	5	4	14	1	0	1	1	1
	69	w				16	0	1	1	99	99
	69	w				21	1	0	0	1	1
	69	w				22	1	1	0	0	1
	69	w				24	1	1	1	2	1
	69	w				27	0	1	1	99	99
	69	w				35	1	0	0	0	1
1345.	55	w	17	3	4	13	1	1	0	1	1
	55	w				15	0	1	1	99	99
	55	w				34	1	1	0	0	0
	55	w				33	1	1	0	1	1
1349.	39	m	25	2	3	37	1	1	1	1	1
	39	m				36	0	1	1	99	99
	39	m				46	1	1	1	1	1
1352.	62	w	16	3	2	12	1	1	0	1	1
	62	w				11	1	1	0	1	0
	62	w				22	1	0	0	0	1
1355.	65	m	25	4	1	16	1	0	0	1	1
	65	m				27	1	0	0	1	1
	65	m				37	1	0	0	1	1
	65	m				35	1	1	0	0	0
1359.	50	w	8	1	4	35	1	1	1	1	1
	50	w				33	0	1	1	99	99
	50	w				41	0	1	1	99	99
	50	w				45	0	1	0	99	99
1363.	60	m	21	7	3	17	1	0	0	1	1
	60	m				15	1	0	1	1	0
	60	m				13	1	0	1	1	0
	60	m				37	0	1	0	99	99
	60	m				36	1	1	0	1	2
	60	m				35	1	0	0	1	0
	60	m				34	1	0	0	0	1
	60	m				46	1	1	0	0	0
1371.	38	w	27	3	1	17	1	0	0	1	1

	38	w				14	1	1	0	1	1
	38	w				35	1	0	1	0	1
1374.	41	m	28	1	0	16	1	0	0	0	1
1375.	73	w	6	1	2	43	1	0	0	0	0
	73	w				32	0	1	0	99	99
	73	w				44	0	1	0	99	99
1378.	24	m	25	2	2	36	1	1	1	1	1
	24	m				46	1	1	1	0	1
1380.	43	w	27	1	0	36	1	0	1	1	1
1381.	48	m	28	1	1	36	1	1	1	1	1
1382.	37	m	28	2	0	17	1	99	99	1	1
	37	m				47	1	0	0	1	1
1384.	62	m	7	2	1	13	1	0	1	0	0
	62	m				44	0	1	1	99	99
	62	m				23	1	99	1	1	1
1387.	49	w	28	2	0	14	1	0	0	1	1
	49	w				11	1	0	0	0	1
1389.	43	w	26	7	1	17	1	0	0	1	1
	43	w				16	1	0	0	1	1
	43	w				15	1	0	0	1	1
	43	w				26	1	0	1	1	1
	43	w				27	1	1	1	1	1
	43	w				45	1	0	1	0	1
	43	w				46	1	0	1	1	1
1396.	53	w	8	3	3	35	1	1	0	1	1
	53	w				34	1	0	0	0	1
	53	w				33	1	1	1	0	1
	53	w				44	0	1	0	99	99
1400.	71	m	22	1	0	24	1	0	0	2	1
1401.	23	m	28	1	1	36	1	1	0	0	0
1402.	77	w	14	2	1	25	1	1	1	1	1
	77	w				43	1	0	0	0	1
1404.	67	w	28	1	2	36	0	1	0	99	99
	67	w				46	1	1	0	1	1
1406.	39	w	26	4	3	25	1	0	0	0	1
	39	w				37	1	1	0	1	1
	39	w				36	1	1	0	1	1
	39	w				45	1	1	0	0	1
1410.	46	w	20	2	3	17	1	1	1	1	1
	46	w				26	0	1	1	99	99
	46	w				47	1	1	0	0	1
1413.	65	w	18	2	0	35	1	0	0	0	0
	65	w				45	1	0	0	0	0
1415.	41	w	23	3	1	14	1	0	1	0	1
	41	w				25	1	0	0	0	1
	41	w				26	1	1	1	1	2
1418.	59	w	12	2	0	13	1	0	0	1	0
	59	w				22	1	0	0	0	0
1420.	25	w	28	2	0	15	1	0	0	0	0
	25	w				36	1	0	0	0	0
1422.	60	w	19	6	0	16	1	0	0	1	1
	60	w				15	1	0	1	0	1
	60	w				14	1	0	0	0	0
	60	w				11	1	0	0	0	0
	60	w				24	1	0	1	0	0
	60	w				25	1	0	0	0	1
1428.	56	m	10	1	1	13	1	0	1	0	1
	56	m				23	0	1	1	99	99
1430.	53	w	24	9	6	16	1	99	1	1	1
	53	w				12	1	99	99	1	1
	53	w				22	1	1	0	1	0
	53	w				23	1	1	1	1	0
	53	w				24	1	1	0	1	1
	53	w				37	1	1	1	1	1
	53	w				35	1	0	0	0	0
	53	w				46	1	1	1	1	1
	53	w				47	1	1	0	1	1
1439.	75	m	21	5	1	15	1	0	1	0	1
	75	m				14	1	1	1	0	0
	75	m				26	1	0	1	1	0
	75	m				33	1	0	0	0	0
	75	m				45	1	0	1	0	1
1444.	67	m	25	5	2	12	1	1	1	1	1
	67	m				26	1	0	0	0	0
	67	m				36	1	1	0	1	1
	67	m				45	1	0	0	0	1
	67	m				46	1	0	0	0	1

1449.	65	w	17	3	1	13	1	0	1	1	1
	65	w				11	1	0	0	1	1
	65	w				26	1	0	0	0	1
	65	w				35	0	1	1	99	99
1453.	63	m	15	7	3	25	1	1	99	2	1
	63	m				26	1	0	99	2	1
	63	m				27	1	1	1	2	1
	63	m				35	1	1	99	1	1
	63	m				43	1	99	99	1	0
	63	m				44	1	99	99	99	99
	63	m				45	1	99	99	1	1
1460.	53	w	22	2	2	23	1	1	0	0	1
	53	w				26	1	1	0	1	1
1462.	59	m	18	3	4	15	1	0	0	1	1
	59	m				14	0	1	1	99	99
	59	m				37	0	1	1	99	99
	59	m				35	1	1	0	0	1
	59	m				45	1	0	0	1	1
	59	m				47	0	1	0	99	99
1468.	46	w	23	8	1	14	1	0	0	1	1
	46	w				13	1	0	1	1	1
	46	w				11	1	0	1	1	1
	46	w				24	1	0	1	1	0
	46	w				27	1	0	0	1	1
	46	w				37	1	1	0	1	1
	46	w				31	1	0	0	0	1
	46	w				44	1	0	0	0	1
1476.	50	w	28	5	1	11	1	0	1	1	2
	50	w				21	1	0	1	1	0
	50	w				24	1	0	1	1	1
	50	w				25	1	0	0	0	1
	50	w				26	1	1	0	1	1
1481.	70	w	21	4	1	12	1	0	0	0	1
	70	w				26	1	0	1	1	1
	70	w				35	1	1	0	0	0
	70	w				46	1	0	0	1	1
1485.	62	m	26	5	4	15	1	1	1	1	1
	62	m				23	1	1	0	1	1
	62	m				24	1	0	0	1	0
	62	m				25	1	1	0	0	0
	62	m				26	0	1	0	99	99
	62	m				45	1	0	0	2	1
1491.	49	w	28	3	2	16	1	1	99	1	1
	49	w				15	1	1	99	0	1
	49	w				25	1	0	0	0	1
1494.	74	m	20	4	1	15	1	1	0	1	1
	74	m				14	1	0	1	1	1
	74	m				45	1	0	0	1	1
	74	m				46	1	0	0	0	1
1498.	56	m	25	4	3	15	1	0	1	0	1
	56	m				24	1	0	0	1	1
	56	m				26	1	1	1	2	1
	56	m				36	1	1	1	1	1
	56	m				44	0	1	1	99	99
1503.	54	w	25	6	3	12	1	1	0	1	0
	54	w				11	1	1	0	0	0
	54	w				21	1	0	1	1	1
	54	w				22	1	0	0	1	0
	54	w				35	1	0	1	1	0
	54	w				47	1	1	0	1	1
1509.	47	m	21	2	3	16	0	1	0	99	99
	47	m				24	0	1	1	99	99
	47	m				25	1	1	0	1	0
	47	m				26	1	0	1	1	0
1513.	58	m	23	1	0	15	1	0	0	1	1
1514.	32	m	27	4	2	12	1	1	1	1	2
	32	m				21	1	99	0	1	99
	32	m				36	1	1	0	1	1
	32	m				45	1	0	1	1	0
1518.	55	w	17	6	4	16	1	0	0	1	1
	55	w				14	1	1	1	0	0
	55	w				11	1	0	0	1	0
	55	w				22	1	1	1	0	99
	55	w				23	1	1	1	0	0
	55	w				24	1	0	1	1	1
	55	w				26	0	1	1	99	99
1525.	40	w	24	6	2	15	1	0	1	1	0

	40	w				21	1	0	0	1	1
	40	w				25	1	0	1	0	0
	40	w				36	1	1	0	1	1
	40	w				35	1	1	0	1	1
	40	w				34	1	0	0	1	0
1531.	64	w	26	6	1	12	1	0	0	0	0
	64	w				22	1	99	0	2	1
	64	w				24	1	0	1	1	1
	64	w				25	1	1	1	1	1
	64	w				45	1	0	0	0	1
	64	w				47	1	0	0	0	1
1537.	53	w	25	2	0	12	1	0	0	1	0
	53	w				26	1	0	1	1	1
1539.	23	w	28	3	2	17	1	1	1	1	1
	23	w				16	1	1	0	1	1
	23	w				47	1	0	1	1	1
1542.	73	m	22	2	2	26	1	0	0	1	1
	73	m				35	0	1	0	99	99
	73	m				46	1	1	0	1	1
1545.	40	w	23	5	0	17	1	0	0	1	1
	40	w				16	1	0	0	1	1
	40	w				23	1	0	1	0	1
	40	w				37	1	0	1	1	1
	40	w				47	1	0	0	1	1
1550.	20	w	28	2	1	26	1	1	0	1	1
	20	w				46	1	0	0	0	0
1552.	70	w	24	4	2	16	1	0	1	2	1
	70	w				24	1	1	0	2	1
	70	w				25	1	1	1	0	1
	70	w				35	1	0	0	1	1
1556.	78	w	22	3	1	22	1	0	0	1	2
	78	w				24	1	1	0	1	1
	78	w				36	1	0	1	0	1
1559.	36	w	28	2	2	21	1	1	1	1	0
	36	w				26	1	1	1	1	1
1561.	87	w	16	2	2	33	1	1	0	1	1
	87	w				45	1	1	1	1	1
1563.	74	w	9	3	1	13	1	0	1	1	1
	74	w				12	1	0	1	0	0
	74	w				21	1	0	1	0	0
	74	w				26	0	1	0	99	99
1567.	73	m	5	1	1	14	1	0	1	1	1
	73	m				16	0	1	1	99	99
1569.	52	w	24	1	2	26	1	1	1	1	2
	52	w				33	0	1	1	99	99
1571.	75	m	20	1	0	25	1	0	1	2	1
1572.	91	m	4	2	2	13	1	0	99	1	1
	91	m				12	1	1	0	1	1
1574.	68	w	28	1	0	17	1	0	0	1	0
1575.	66	w	26	13	3	17	1	1	0	0	1
	66	w				16	1	0	0	1	0
	66	w				14	1	0	0	1	0
	66	w				11	1	0	0	1	0
	66	w				21	1	0	0	1	0
	66	w				22	1	0	0	1	1
	66	w				24	1	99	1	0	1
	66	w				26	1	0	0	0	1
	66	w				35	1	0	0	1	1
	66	w				34	1	1	0	0	0
	66	w				44	1	1	0	0	0
	66	w				45	1	0	0	0	1
	66	w				47	1	0	0	2	1
1588.	44	w	25	2	99	12	1	99	0	1	0
	44	w				11	1	99	0	0	0
1590.	59	m	28	5	1	14	1	0	0	1	1
	59	m				22	1	0	1	1	1
	59	m				26	1	1	1	1	1
	59	m				45	1	0	0	1	1
	59	m				46	1	99	0	0	1
1595.	67	w	16	3	0	14	1	0	0	1	1
	67	w				25	1	0	1	1	99
	67	w				34	1	0	0	0	0
1598.	64	m	18	6	3	16	1	0	1	1	1
	64	m				14	1	1	0	1	1
	64	m				13	1	0	0	1	1
	64	m				21	1	1	0	1	1
	64	m				32	1	99	99	99	99

	64	m				46	1	1	0	1	99
1604.	35	m	25	1	1	21	1	1	0	1	1
1605.	58	w	14	1	0	11	1	0	1	0	0
1606.	44	m	28	0	1	36	0	1	99	99	99
1607.	72	w	20	4	1	12	1	0	0	0	0
	72	w				21	1	0	1	1	1
	72	w				22	1	0	1	1	1
	72	w				23	1	0	0	0	1
	72	w				35	0	1	0	99	99
1612.	34	w	26	4	0	16	1	0	1	0	1
	34	w				21	1	0	0	0	1
	34	w				22	1	99	0	1	0
	34	w				26	1	0	0	1	1
1616.	42	m	28	1	1	36	1	1	0	0	0
1617.	72	w	10	1	0	23	1	0	1	0	0
1618.	73	m	20	5	1	21	1	1	0	1	1
	73	m				23	1	0	0	1	0
	73	m				24	1	0	0	1	1
	73	m				26	1	0	0	1	1
	73	m				35	1	0	0	1	0
1623.	38	w	26	1	1	46	1	0	1	0	1
	38	w				16	0	1	1	99	99
1625.	31	m	24	3	1	15	1	0	1	0	1
	31	m				11	0	1	1	99	99
	31	m				21	1	0	0	1	0
	31	m				47	1	0	0	0	1
1629.	53	w	24	5	2	17	1	1	0	0	1
	53	w				25	1	0	1	0	1
	53	w				26	1	0	0	2	1
	53	w				46	1	0	0	0	1
	53	w				47	1	1	0	1	1
1634.	67	m	5	4	2	33	1	0	0	1	1
	67	m				32	1	1	1	0	1
	67	m				43	1	0	0	0	1
	67	m				44	1	1	0	1	2
1638.	57	m	25	3	1	16	1	1	0	1	1
	57	m				14	1	0	1	1	1
	57	m				36	1	0	0	1	1
1641.	23	m	28	2	2	15	1	1	0	1	1
	23	m				26	1	1	1	1	1
1643.	43	w	26	3	1	25	1	1	0	0	1
	43	m				27	1	0	0	1	1
	43	m				36	1	1	1	1	1
1646.	52	m	26	3	2	12	1	0	0	1	1
	52	m				25	1	0	0	1	1
	52	m				36	1	1	1	1	1
	52	m				47	0	1	0	99	99
1650.	49	w	28	1	0	36	1	0	0	1	1
1651.	63	m	24	7	5	15	1	1	1	1	1
	63	m				14	1	0	1	1	1
	63	m				25	1	0	0	0	1
	63	m				26	1	1	1	2	1
	63	m				35	1	1	0	0	1
	63	m				43	1	1	0	1	1
	63	m				47	1	1	1	1	1
1658.	55	m	25	4	2	21	1	0	0	1	1
	55	m				22	1	1	0	2	1
	55	m				35	1	0	0	0	0
	55	m				46	1	1	0	0	1
1662.	53	m	25	8	5	16	1	0	1	1	1
	53	m				13	1	1	99	99	1
	53	m				21	1	1	99	99	99
	53	m				24	1	99	1	99	99
	53	m				25	1	99	1	99	99
	53	m				26	1	1	1	1	1
	53	m				37	0	1	1	99	99
	53	m				36	0	1	1	99	99
	53	m				34	1	0	0	1	1
	53	m				33	1	0	0	1	1
1672.	36	w	27	4	2	17	1	0	0	1	1
	36	w				14	1	0	0	1	1
	36	w				37	1	1	0	0	1
	36	w				46	1	1	1	1	1
1676.	54	w	24	6	2	16	1	1	0	1	1
	54	w				15	1	0	1	1	0
	54	w				24	1	1	0	1	1
	54	w				25	1	0	1	0	1

	54	w				26	1	0	1	1	1
	54	w				35	1	0	0	1	1
1682.	48	m	24	6	5	17	1	1	1	1	1
	48	m				16	1	1	0	1	0
	48	m				26	1	1	1	1	1
	48	m				27	1	0	0	0	1
	48	m				36	1	1	0	1	1
	48	m				31	1	0	0	0	2
	48	m				46	0	1	0	99	99
1689.	53	m	18	3	0	33	1	0	0	0	1
	53	m				41	1	0	1	0	1
	53	m				43	1	0	0	1	0
1692.	52	m	17	8	4	16	1	0	1	1	1
	52	m				15	1	0	0	1	1
	52	m				13	1	0	1	0	1
	52	m				12	1	0	0	1	1
	52	m				22	1	1	1	1	2
	52	m				23	1	1	1	0	2
	52	m				26	1	1	1	1	1
	52	m				33	0	1	1	99	99
	52	m				43	1	0	0	1	0
1701.	54	m	26	3	3	15	1	0	0	0	1
	54	m				44	1	1	0	0	1
	54	m				45	1	0	1	1	1
	54	m				46	0	1	1	99	99
	54	m				47	0	1	1	99	99
1706.	51	m	26	4	1	16	1	1	0	1	1
	51	m				25	1	0	1	1	0
	51	m				46	1	0	1	1	1
	51	m				47	1	0	1	1	0
1710.	69	w	13	5	0	14	1	0	1	1	1
	69	w				13	1	0	1	1	0
	69	w				12	1	0	0	1	0
	69	w				43	1	0	1	0	1
	69	w				47	1	0	1	1	1
1715.	49	w	19	8	3	11	1	0	1	1	0
	49	w				21	1	1	0	1	0
	49	w				22	1	0	0	1	1
	49	w				24	1	0	0	1	1
	49	w				25	1	1	0	1	1
	49	w				35	1	0	0	1	1
	49	w				34	1	0	1	1	1
	49	w				45	1	1	99	1	1
1723.	55	m	28	2	2	37	1	1	1	1	1
	55	m				36	1	1	0	1	1
1725.	40	w	28	1	0	35	1	0	0	0	0
1726.	21	m	27	2	2	16	1	1	0	1	0
	21	m				15	1	0	0	1	0
	21	m				26	0	1	1	99	99
1729.	62	w	24	1	1	15	1	1	1	0	1
1730.	77	m	14	6	0	21	1	0	0	1	0
	77	m				23	1	0	1	1	0
	77	m				25	1	0	0	1	1
	77	m				37	1	0	0	2	1
	77	m				35	1	0	0	1	1
	77	m				46	1	0	1	2	1
1736.	35	w	27	2	2	17	1	1	1	1	99
	35	w				36	1	1	1	1	0
1738.	58	w	24	3	0	26	1	0	0	1	1
	58	w				27	1	0	1	1	1
	58	w				46	1	0	1	2	1
1741.	63	m	16	2	1	23	1	0	1	1	1
	63	m				45	1	1	0	1	2
1743.	61	m	27	8	2	16	1	0	0	0	1
	61	m				21	1	0	1	0	0
	61	m				24	1	0	0	0	1
	61	m				25	1	1	0	1	1
	61	m				26	1	1	0	1	1
	61	m				27	1	0	1	1	1
	61	m				34	1	0	1	1	1
	61	m				44	1	0	1	1	0
1751.	60	w	22	2	0	15	1	0	0	0	1
	60	w				13	1	0	0	0	0
1753.	48	w	21	3	1	37	1	1	0	1	1
	48	w				36	1	0	0	1	1
	48	w				43	1	0	0	1	1
1756.	54	w	25	1	1	41	1	1	0	1	1

1757.	58	w	20	3	2	17	1	0	1	1	1
	58	w				22	1	0	0	1	0
	58	w				36	0	1	1	99	99
	58	w				24	1	1	0	1	1
1761.	28	w	28	3	1	11	1	1	0	0	0
	28	w				22	1	99	99	0	0
	28	w				26	1	0	0	1	1
1764.	23	w	27	3	3	12	1	1	0	1	1
	23	w				36	1	1	1	0	0
	23	w				46	1	1	0	0	0
1767.	39	w	27	10	6	17	1	1	1	1	1
	39	w				16	1	1	1	1	1
	39	w				15	0	1	1	99	99
	39	w				14	1	0	0	1	0
	39	w				12	1	1	0	1	1
	39	w				11	1	0	0	1	1
	39	w				25	1	0	0	1	1
	39	w				36	1	1	0	1	1
	39	w				35	1	1	99	1	1
	39	w				44	1	0	0	1	0
	39	w				45	1	0	0	0	1
1778.	62	w	26	2	2	12	1	1	99	1	1
	62	w				11	1	1	0	0	0
1780.	50	w	21	5	2	16	1	0	1	1	1
	50	w				14	1	1	1	1	1
	50	w				37	1	0	0	2	1
	50	w				36	1	1	1	1	1
	50	w				35	1	0	1	1	1
1785.	45	w	28	1	0	25	1	0	0	0	1
1786.	63	m	21	4	1	24	1	1	0	1	1
	63	m				26	1	99	1	0	1
	63	m				27	1	99	1	1	1
	63	m				45	1	0	0	1	2
1790.	46	w	23	9	2	22	1	0	0	1	1
	46	w				23	1	0	0	1	2
	46	w				25	1	1	0	0	1
	46	w				26	1	0	1	1	1
	46	w				37	1	0	0	0	2
	46	w				36	1	0	0	0	1
	46	w				45	1	0	0	0	1
	46	w				46	1	0	0	1	1
	46	w				47	1	1	0	1	1
1799.	82	w	4	2	1	21	1	1	1	1	1
	82	w				22	1	0	1	1	1
1801.	70	w	16	1	0	21	1	0	0	1	1
1802.	57	w	26	3	2	24	1	1	0	1	1
	57	w				43	1	0	0	1	1
	57	w				46	1	1	0	1	1
1805.	64	w	18	10	3	13	1	0	1	1	0
	64	w				12	1	99	99	99	99
	64	w				11	1	1	0	1	1
	64	w				24	1	99	99	99	99
	64	w				25	1	0	0	0	99
	64	w				37	1	1	0	0	0
	64	w				34	1	0	0	0	0
	64	w				33	1	0	0	0	1
	64	w				43	1	1	0	1	1
	64	w				44	1	0	1	0	1
1815.	39	m	25	4	1	15	1	0	0	1	1
	39	m				24	1	0	0	1	1
	39	m				37	1	0	1	1	1
	39	m				35	1	1	0	1	2
1819.	60	m	22	2	5	17	0	1	0	99	99
	60	m				16	1	0	1	1	1
	60	m				12	0	1	1	99	99
	60	m				22	0	1	0	99	99
	60	m				24	1	1	0	1	1
	60	m				45	0	1	1	99	99
1825.	65	m	26	1	3	23	1	1	0	0	1
	65	m				26	0	1	1	99	99
	65	m				37	0	1	1	99	99
1827.	42	w	25	0	1	37	0	1	1	99	99
1828.	70	w	18	7	3	17	0	1	1	99	99
	70	w				14	1	0	1	1	1
	70	w				12	1	0	0	0	1
	70	w				23	1	1	0	1	0
	70	w				24	1	0	0	1	1

	70	w				26	1	1	0	1	1
	70	w				34	1	0	0	0	1
	70	w				44	1	0	0	1	1
1836.	57	m	18	1	2	22	0	1	1	99	99
	57	m				23	0	1	1	99	99
	57	m				35	1	0	0	1	0
1839.	43	w	27	1	2	14	1	1	1	1	1
	43	w				25	0	1	1	99	99
1841.	55	w	23	6	4	12	1	1	99	1	99
	55	w				11	1	1	99	1	99
	55	w				24	1	1	1	0	0
	55	w				35	1	0	0	0	0
	55	w				46	1	1	0	1	1
	55	w				47	1	0	1	1	1
1847.	36	m	21	1	2	24	1	1	99	1	1
	36	m				26	0	1	1	99	99
1849.	46	m	24	2	2	26	1	1	0	1	1
	46	m				35	1	1	0	0	1
1851.	76	w	20	2	2	16	0	1	0	99	99
	76	w				44	1	1	1	0	0
	76	w				45	1	0	1	0	2
1854.	33	m	26	3	4	16	0	1	1	99	99
	33	m				25	1	0	1	0	1
	33	m				37	0	1	1	99	99
	33	m				36	1	1	0	1	1
	33	m				35	1	1	1	1	1
1859.	57	w	21	7	6	15	1	1	1	1	1
	57	w				14	1	1	1	1	1
	57	w				21	0	1	1	99	99
	57	w				24	1	0	0	1	1
	57	w				26	1	1	1	1	1
	57	w				33	1	1	1	2	1
	57	w				44	1	1	1	1	1
	57	w				45	1	0	1	0	1
1867.	60	w	18	9	2	17	1	0	1	1	1
	60	w				15	1	1	1	1	1
	60	w				14	1	0	0	0	0
	60	w				13	1	1	0	1	1
	60	w				12	1	0	1	0	1
	60	w				22	1	0	1	1	1
	60	w				23	1	0	0	0	1
	60	w				27	1	0	1	1	1
	60	w				46	1	0	0	0	1
1876.	47	m	26	2	1	14	1	99	0	1	0
	47	m				21	1	1	0	0	0
1878.	67	m	19	4	4	14	1	99	1	1	1
	67	m				12	1	1	0	1	1
	67	m				27	1	1	1	0	1
	67	m				43	0	1	1	99	99
	67	m				47	1	1	0	0	1
1883.	57	w	24	2	2	17	1	1	1	1	2
	57	w				46	1	1	0	1	1
1885.	73	w	18	9	3	15	1	0	1	2	1
	73	w				13	1	0	1	0	0
	73	w				23	1	0	0	1	1
	73	w				25	1	0	1	1	1
	73	w				26	1	0	0	1	1
	73	w				35	1	1	1	1	0
	73	w				33	1	0	0	1	1
	73	w				41	1	1	1	0	99
	73	w				43	1	1	0	1	1
1894.	58	w	21	9	4	14	1	0	1	1	1
	58	w				13	1	0	1	0	0
	58	w				12	1	0	1	0	1
	58	w				21	1	0	1	0	1
	58	w				25	1	1	1	0	1
	58	w				36	0	1	1	99	99
	58	w				35	1	0	0	1	1
	58	w				34	1	0	1	0	0
	58	w				44	1	1	0	0	2
	58	w				47	1	1	0	1	1
1904.	29	m	28	2	4	14	1	1	1	0	1
	29	m				26	0	1	0	99	99
	29	m				36	1	1	0	1	1
	29	m				46	0	1	0	99	99
1908.	82	m	19	1	1	35	1	0	1	1	1
	82	m				32	0	1	1	99	99

1910.	65	m	21	1	1	23	0	1	1	99	99
	65	m				35	1	0	0	1	1
1912.	60	w	25	4	6	14	0	1	0	99	99
	60	w				12	1	0	1	1	1
	60	w				11	1	1	99	0	1
	60	w				23	1	99	0	1	0
	60	w				26	0	1	1	99	99
	60	w				27	0	1	1	99	99
	60	w				35	1	1	1	0	1
	60	w				45	0	1	0	99	99
1920.	60	m	8	1	1	46	1	1	0	0	1
1921.	65	w	12	0	5	23	0	1	1	99	99
	65	w				25	0	1	1	99	99
	65	w				31	0	1	1	99	99
	65	w				43	0	1	0	99	99
	65	w				47	0	1	1	99	99
1926.	29	w	28	1	0	26	1	0	0	1	0
1927.	53	m	28	3	0	21	1	0	0	1	99
	53	m				31	1	0	0	0	0
	53	m				41	1	0	0	0	0
1930.	59	m	16	5	1	17	0	1	1	99	99
	59	m				21	1	99	1	1	99
	59	m				22	1	99	1	0	99
	59	m				35	1	0	0	0	0
	59	m				44	1	0	1	0	1
	59	m				45	1	0	0	0	1
1936.	69	m	25	7	10	16	0	1	1	99	99
	69	m				15	0	1	1	99	99
	69	m				14	1	1	99	2	1
	69	m				13	0	1	1	99	99
	69	m				12	0	1	1	99	99
	69	m				21	1	0	0	0	1
	69	m				25	1	1	1	1	1
	69	m				27	1	1	99	2	1
	69	m				37	1	0	0	1	1
	69	m				36	0	1	1	99	99
	69	m				41	1	1	0	0	1
	69	m				47	1	1	1	1	1
1948.	39	m	27	3	1	12	1	0	0	1	0
	39	m				22	1	0	0	0	0
	39	m				46	1	1	0	0	0
1951.	52	m	26	1	3	15	1	1	99	1	99
	52	m				37	0	1	1	99	99
	52	m				46	0	1	1	99	99
1954.	50	m	28	1	0	24	1	0	0	1	1
1955.	51	w	23	5	4	16	0	1	1	99	99
	51	w				15	1	0	1	1	1
	51	w				14	1	0	1	1	1
	51	w				37	1	1	1	1	1
	51	w				36	1	1	1	1	1
	51	w				45	1	1	0	1	1
1961.	51	m	17	7	0	16	1	0	0	0	1
	51	m				13	1	0	1	0	1
	51	m				23	1	0	1	1	0
	51	m				35	1	0	0	1	1
	51	m				34	1	0	0	0	1
	51	m				44	1	0	0	1	1
	51	m				45	1	0	0	0	1
1968.	76	m	18	1	0	14	1	0	0	1	1
1969.	74	w	10	1	0	15	1	0	1	1	1
1970.	32	w	28	4	4	21	1	1	0	1	1
	32	m				26	1	1	1	1	99
	32	m				31	1	1	1	1	1
	32	m				41	1	1	1	1	1
1974.	64	m	25	1	0	35	1	0	0	0	0
1975.	77	m	27	2	0	23	1	0	0	0	0
	77	m				27	1	0	0	1	1
1977.	47	w	12	3	2	25	1	0	99	1	1
	47	w				27	1	1	1	1	1
	47	w				33	0	1	1	99	99
	47	w				43	1	0	0	1	0
1981.	60	m	19	3	0	16	1	0	0	0	1
	60	m				24	1	0	0	1	1
	60	m				26	1	0	0	1	1
1984.	47	m	27	4	1	12	1	1	0	1	99
	47	m				22	1	0	0	0	0
	47	m				25	1	0	0	1	0

	47	m				27	1	0	0	1	1
1988.	45	w	27	1	99	13	1	99	99	1	99
1989.	44	w	17	3	0	25	1	0	0	0	1
	44	w				26	1	0	1	1	1
	44	w				35	1	0	0	1	0
1992.	73	w	21	3	1	15	1	0	0	1	1
	73	w				45	1	0	0	1	1
	73	w				46	1	1	0	1	1
1995.	31	m	27	1	1	16	1	1	0	0	1
1996.	50	w	13	5	2	16	1	0	0	0	1
	50	w				24	1	0	0	1	1
	50	w				33	1	0	1	1	1
	50	w				32	1	1	99	99	99
	50	w				43	1	1	1	0	1
2001.	55	w	23	3	1	24	1	0	0	0	1
	55	w				36	1	0	0	1	1
	55	w				37	0	1	1	99	99
	55	w				35	1	0	0	1	1
2005.	51	m	20	2	1	22	1	0	1	1	0
	51	m				36	1	1	0	1	1
2007.	51	m	27	5	0	16	1	0	0	1	0
	51	m				26	1	0	0	1	0
	51	m				36	1	0	0	0	0
	51	m				45	1	0	0	1	0
	51	m				46	1	0	0	1	1
2012.	52	w	23	1	2	17	1	0	0	1	1
	52	w				14	0	1	1	99	99
	52	w				36	0	1	1	99	99
2015.	38	w	18	2	1	14	1	0	1	1	0
	38	w				16	0	1	1	99	99
	38	w				25	1	0	1	1	1
2018.	73	m	26	4	2	15	1	0	0	1	1
	73	m				23	1	1	1	1	1
	73	m				24	0	1	1	99	99
	73	m				37	1	0	0	0	0
	73	m				44	1	99	99	99	99
2023.	65	w	25	3	1	24	1	1	1	0	1
	65	w				35	1	0	0	0	1
	65	w				45	1	0	1	0	0
2026.	51	w	24	2	0	14	1	0	1	1	1
	51	w				46	1	0	1	1	0
2028.	57	w	24	6	1	17	1	0	0	1	1
	57	w				13	1	0	1	0	1
	57	w				22	1	99	0	0	1
	57	w				24	1	0	1	0	1
	57	w				46	1	0	1	0	1
	57	w				47	1	1	1	1	1
2034.	41	w	28	1	0	35	1	0	0	0	0
2035.	31	w	28	2	0	17	1	0	0	0	0
	31	w				15	1	0	0	1	1
2037.	42	w	26	5	2	16	1	0	1	1	1
	42	w				15	1	1	1	0	1
	42	w				12	1	99	0	0	1
	42	w				11	1	1	0	0	0
	42	w				21	1	99	0	0	0
2042.	60	m	10	2	1	14	1	1	0	1	1
	60	m				24	1	0	1	0	1
2044.	78	m	17	1	1	23	1	99	99	1	1
	78	m				45	0	1	1	99	99
2046.	44	w	24	7	4	22	1	0	1	0	1
	44	w				25	1	0	0	0	2
	44	w				26	1	0	1	2	1
	44	w				27	1	1	1	0	1
	44	w				36	1	1	0	1	1
	44	w				35	1	1	0	1	1
	44	w				45	1	1	99	99	1
2053.	70	m	14	8	6	13	1	1	0	1	1
	70	m				14	1	1	1	99	99
	70	m				12	1	0	0	1	0
	70	m				21	1	1	0	1	1
	70	m				22	1	1	1	1	1
	70	m				24	1	1	1	1	1
	70	m				37	1	0	0	1	99
	70	m				33	1	1	1	0	1
2061.	50	m	28	1	0	36	1	0	1	0	1
2062.	56	w	10	2	1	45	1	0	0	1	0
	56	w				47	1	1	0	2	1

2064.	59	w	25	6	4	16	1	1	99	1	1
	59	w				17	0	1	1	99	99
	59	w				12	1	1	0	0	0
	59	w				11	1	0	1	0	0
	59	w				25	1	0	1	1	1
	59	w				26	1	1	0	0	99
	59	w				32	1	0	0	1	0
2071.	42	w	27	2	2	15	1	0	1	1	1
	42	w				24	0	1	1	99	99
	42	w				26	1	1	99	1	2
2074.	50	w	23	3	2	22	1	1	0	1	1
	50	w				26	1	1	1	1	1
	50	w				37	1	0	1	0	0
2077.	57	m	21	5	3	16	1	1	1	1	1
	57	m				15	1	0	0	1	1
	57	m				14	1	0	0	1	1
	57	m				37	1	1	1	1	1
	57	m				47	1	1	1	1	1
2082.	45	w	26	1	0	15	1	0	0	1	0
2083.	24	m	27	1	0	36	1	0	1	0	1
2084.	21	m	28	0	1	26	0	1	1	99	99
2085.	51	m	26	3	2	17	1	0	1	1	0
	51	m				16	1	1	1	1	1
	51	m				14	1	1	99	0	1
2088.	72	m	18	1	2	16	0	1	1	99	99
	72	m				13	0	1	1	99	99
	72	m				25	1	0	1	1	1
2091.	48	w	26	2	1	26	1	1	0	0	0
	48	w				35	1	0	0	1	0
2093.	55	w	24	7	2	17	1	99	99	1	1
	55	w				11	1	1	1	1	1
	55	w				21	1	0	0	99	99
	55	w				24	1	0	0	0	0
	55	w				45	1	0	0	2	1
	55	w				46	1	0	0	1	1
	55	w				47	1	1	0	1	1
2100.	63	w	19	5	0	14	1	0	1	1	1
	63	w				11	1	0	1	1	1
	63	w				21	1	0	0	1	1
	63	w				23	1	0	1	1	1
	63	w				46	1	0	1	1	1
2105.	72	w	18	3	0	33	1	0	99	99	99
	72	w				43	1	0	99	1	1
	72	w				45	1	0	99	1	99
2108.	63	w	27	6	2	15	1	0	0	0	1
	63	w				14	1	0	1	1	1
	63	w				36	1	1	1	2	1
	63	w				35	1	0	0	0	1
	63	w				34	1	0	0	1	1
	63	w				45	1	1	0	0	1
2114.	77	w	9	1	0	34	1	0	1	1	0
2115.	74	w	19	6	3	17	1	0	0	1	1
	74	w				16	1	0	0	1	1
	74	w				15	1	0	1	1	0
	74	w				14	1	1	0	1	1
	74	w				13	1	1	0	1	1
	74	w				37	1	1	0	0	1
2121.	48	w	21	2	2	16	1	1	1	1	1
	48	w				14	1	1	1	0	1
2123.	21	w	28	1	0	11	1	0	0	1	0
2124.	46	m	25	3	1	12	1	0	0	1	0
	46	m				25	1	99	0	99	99
	46	m				26	0	1	1	99	99
	46	m				27	1	0	1	1	1
2128.	74	m	25	3	1	15	1	0	0	1	1
	74	m				35	1	0	0	1	0
	74	m				44	1	0	1	1	1
	74	m				45	0	1	1	99	99
2132.	48	m	27	3	1	25	1	0	1	1	1
	48	m				26	1	1	1	1	1
	48	m				35	1	0	0	0	1
2135.	27	w	22	6	2	13	1	1	1	1	1
	27	w				12	1	0	1	0	1
	27	w				11	1	0	1	1	0
	27	w				22	1	0	1	1	1
	27	w				25	1	0	1	1	1
	27	w				26	1	1	1	1	1

2141.	78	m	22	6	3	15	1	0	0	1	1
	78	m				14	1	1	1	1	1
	78	m				24	1	1	1	99	1
	78	m				37	1	1	99	1	1
	78	m				44	1	0	1	0	1
	78	m				45	1	0	1	0	1
2147.	72	w	18	1	99	47	1	99	1	1	1
2148.	42	w	28	1	0	16	1	0	0	1	0
2149.	26	w	28	3	3	16	1	1	99	1	1
	26	w				15	1	1	1	1	1
	26	w				36	1	1	0	1	1
2152.	23	w	28	1	0	21	1	0	0	1	0
2153.	21	m	27	1	0	12	1	0	0	0	1
2154.	48	m	28	0	1	46	0	1	1	99	99
2155.	52	w	24	2	1	12	1	0	0	0	0
	52	w				46	1	1	0	1	1
2157.	78	m	24	1	1	15	0	1	99	99	99
	78	m				11	1	0	1	1	0
2159.	61	w	21	11	3	14	1	0	0	99	1
	61	w				11	1	0	0	0	0
	61	w				21	1	0	0	1	1
	61	w				22	1	0	0	0	1
	61	w				23	1	1	0	0	1
	61	w				24	1	0	1	1	1
	61	w				25	1	0	1	1	1
	61	w				35	1	0	0	0	0
	61	w				33	1	1	99	1	99
	61	w				42	1	0	0	0	0
	61	w				45	1	1	0	0	0
2170.	70	w	3	2	1	35	1	0	0	0	1
	70	w				32	1	1	1	1	1
2172.	38	m	28	3	2	24	1	1	0	1	1
	38	m				45	1	0	1	0	0
	38	m				46	1	1	1	1	1
2175.	39	w	26	3	2	15	1	1	0	1	2
	39	w				24	1	0	0	0	1
	39	w				46	1	1	0	0	1
2178.	37	w	28	1	0	46	1	0	1	1	1
2179.	61	w	17	5	3	16	1	0	0	1	1
	61	w				25	1	1	0	1	1
	61	w				34	1	0	0	1	1
	61	w				33	1	1	0	1	1
	61	w				44	1	1	0	1	0
2184.	52	m	27	5	1	15	1	0	1	1	1
	52	m				14	1	0	1	1	1
	52	m				11	1	0	1	1	0
	52	m				22	1	0	0	0	0
	52	m				27	1	1	99	1	1
2189.	37	w	28	3	2	16	1	1	1	1	1
	37	w				26	1	0	0	0	0
	37	w				46	1	1	1	0	1
2192.	40	w	24	8	2	15	1	1	0	1	0
	40	w				14	1	1	1	99	99
	40	w				11	1	99	1	1	2
	40	w				21	1	0	0	1	0
	40	w				22	1	99	1	1	99
	40	w				23	1	0	1	0	0
	40	w				26	1	0	0	1	99
	40	w				45	1	0	0	0	0
2200.	44	m	25	1	1	46	1	1	1	1	2
2201.	43	w	27	2	1	16	1	1	1	1	1
	43	w				15	1	0	0	1	0
2203.	47	m	25	2	1	12	1	1	0	1	1
	47	m				11	1	0	0	0	1
2205.	65	w	28	3	3	24	1	1	0	1	1
	65	w				36	1	1	0	1	1
	65	w				46	1	1	1	1	1
2208.	55	w	23	4	1	12	1	1	1	1	1
	55	w				21	1	0	0	1	1
	55	w				44	1	0	1	1	1
	55	w				47	1	0	0	1	1
2212.	46	w	18	1	2	25	1	1	1	0	0
	46	w				46	0	1	1	99	99
2214.	36	w	23	1	0	27	1	0	1	1	1
2215.	45	w	23	6	5	11	1	1	0	1	1
	45	w				21	0	1	1	99	99
	45	w				22	1	1	0	1	1

	45	w				26	1	1	0	1	99
	45	w				37	1	1	0	1	99
	45	w				42	1	0	1	0	1
	45	w				45	1	0	1	1	1
2222.	47	m	26	3	1	15	1	0	0	0	1
	47	m				37	1	0	1	1	1
	47	m				47	1	1	1	1	1
2225.	34	w	27	2	3	12	1	1	1	1	1
	34	w				27	0	1	1	99	99
	34	w				36	1	1	1	1	1
2228.	53	m	26	0	3	17	0	1	1	99	99
	53	m				16	0	1	0	99	99
	53	m				14	0	1	1	99	99
2231.	39	w	28	1	2	22	1	1	1	0	0
	39	w				24	0	1	1	99	99
2233.	79	w	19	3	0	25	1	0	0	2	1
	79	w				34	1	0	0	2	1
	79	w				45	1	99	99	99	1
2236.	58	w	26	11	5	16	1	0	1	1	1
	58	w				15	1	0	0	0	1
	58	w				12	0	0	1	99	99
	58	w				11	1	0	1	0	1
	58	w				22	1	0	1	1	0
	58	w				25	1	0	0	1	1
	58	w				27	1	1	0	2	1
	58	w				31	1	1	1	1	1
	58	w				41	1	1	1	1	1
	58	w				42	1	1	1	0	1
	58	w				46	1	1	1	1	1
	58	w				47	1	0	1	1	1
2248.	71	m	23	3	1	17	1	1	1	1	1
	71	m				15	1	0	1	1	1
	71	m				14	1	0	0	1	1
2251.	80	m	9	1	2	21	1	1	1	1	1
	80	m				22	0	1	0	99	99
2253.	54	w	23	10	2	15	1	1	1	1	1
	54	w				14	1	0	1	0	1
	54	w				13	1	99	99	0	0
	54	w				12	1	0	0	0	0
	54	w				11	1	0	1	1	1
	54	w				21	1	0	0	1	0
	54	w				22	1	0	0	0	0
	54	w				24	1	1	0	1	1
	54	w				25	1	99	99	1	0
	54	w				44	1	0	0	1	1
2263.	66	w	24	4	1	16	1	0	1	1	1
	66	w				21	1	0	1	0	1
	66	w				37	0	1	1	99	99
	66	w				44	1	0	1	0	1
	66	w				46	1	0	1	1	1
2268.	48	m	26	3	3	15	0	1	99	99	99
	48	m				24	1	1	0	0	0
	48	m				27	0	1	1	99	99
	48	m				45	1	0	0	0	1
	48	m				46	1	0	0	1	1
2273.	56	m	21	2	1	23	1	0	1	1	1
	56	m				24	0	1	1	99	99
	56	m				44	1	0	0	0	0
2276.	40	w	26	2	2	15	1	1	0	1	1
	40	w				47	1	1	1	1	1
2278.	62	w	24	0	2	36	0	1	1	99	99
	62	w				47	0	1	1	99	99
2280.	64	w	23	1	1	16	0	1	1	99	99
	64	w				47	1	0	1	0	1
2282.	50	w	11	2	3	17	0	1	1	99	99
	50	w				12	1	1	0	0	1
	50	w				22	1	1	0	0	0
2285.	47	w	20	1	6	14	0	1	1	99	99
	47	w				22	0	1	0	99	99
	47	w				23	0	1	1	99	99
	47	w				24	1	1	99	1	1
	47	w				25	0	1	1	99	99
	47	w				26	0	1	1	99	99
2291.	41	w	28	2	3	16	0	1	1	99	99
	41	w				15	1	1	1	1	1
	41	w				22	1	0	1	1	1
	41	w				36	0	1	1	99	99

2295.	61	w	27	4	4	17	0	1	1	99	99
	61	w				16	1	0	1	1	1
	61	w				15	1	1	1	0	1
	61	w				36	1	1	0	0	1
	61	w				46	1	1	1	0	1
2300.	68	w	23	1	3	26	0	1	1	99	99
	68	w				37	1	1	1	1	1
	68	w				36	0	1	1	99	99
2303.	68	w	26	4	1	26	1	1	0	1	1
	68	w				27	1	0	1	1	1
	68	w				37	1	0	1	1	1
	68	w				35	1	0	0	1	1
2307.	69	w	24	1	4	16	0	1	1	99	99
	69	w				14	1	1	1	0	1
	69	w				23	0	1	1	99	99
	69	w				46	0	1	1	99	99
2311.	65	w	17	5	4	13	1	1	1	0	1
	65	w				11	0	1	1	99	99
	65	w				22	1	1	1	1	1
	65	w				23	1	1	1	1	1
	65	w				34	1	0	1	1	2
	65	w				46	1	0	1	1	1
2317.	48	m	25	5	3	14	1	1	0	0	1
	48	m				13	1	0	0	0	1
	48	m				12	1	1	0	0	1
	48	m				23	1	1	0	1	1
	48	m				24	1	0	1	1	1
2322.	58	w	12	3	3	35	0	1	1	99	99
	58	w				44	1	0	1	1	1
	58	w				45	1	1	1	0	0
	58	w				46	1	1	99	1	1
2326.	41	w	21	1	2	36	0	1	0	99	99
	41	w				46	1	1	1	0	1
2328.	38	m	26	1	1	17	1	1	1	1	1
2330.	66	w	28	0	2	36	0	1	1	99	99
	66	w				47	0	1	1	99	99
2331.	31	w	27	2	3	12	1	1	1	0	1
	31	w				11	1	1	1	0	0
	31	w				26	0	1	1	99	99
2334.	72	w	25	2	3	14	0	1	0	99	99
	72	w				13	1	1	1	1	1
	72	w				46	1	1	0	1	1
2337.	52	m	26	3	3	17	0	1	99	99	99
	52	m				15	1	0	0	0	1
	52	m				37	1	1	0	1	1
	52	m				36	1	1	1	1	1
2341.	60	w	19	7	6	11	1	0	1	0	1
	60	w				21	1	1	1	1	1
	60	w				22	1	1	0	2	1
	60	w				24	1	1	1	1	1
	60	w				25	1	0	1	1	1
	60	w				27	0	1	1	99	99
	60	w				34	0	0	1	99	99
	60	w				35	1	1	0	1	1
	60	w				46	1	1	0	1	1
2350.	22	w	28	0	4	16	0	1	1	99	99
	22	w				27	0	1	1	99	99
	22	w				37	0	1	1	99	99
	22	w				36	0	1	1	99	99
2354.	66	w	27	2	2	16	1	1	0	1	1
	66	w				22	1	1	0	2	1
2356.	38	m	19	1	1	24	1	1	1	1	1
2357.	67	m	14	4	2	21	1	1	0	0	0
	67	m				23	1	1	0	1	2
	67	m				34	1	0	1	1	1
	67	m				46	1	0	0	1	1
2361.	64	w	16	4	2	15	1	0	1	1	1
	64	w				14	1	1	1	0	1
	64	w				13	1	0	0	1	1
	64	w				27	1	0	1	1	1
	64	w				32	0	1	1	99	99
2366.	41	w	24	2	3	27	0	1	1	99	99
	41	w				34	1	1	1	0	1
	41	w				45	1	1	1	1	1
2369.	54	w	26	5	3	15	1	0	1	1	1
	54	w				35	1	1	1	0	1
	54	w				44	1	1	0	0	2

	54	w				45	1	0	0	1	0
	54	w				46	1	1	0	1	1
2374.	79	m	8	3	3	13	1	1	99	1	1
	79	m				11	1	1	1	1	0
	79	m				23	1	1	0	1	2
2377.	72	w	16	1	1	17	0	1	1	99	99
	72	w				22	1	0	0	1	1
2379.	45	w	27	4	3	16	1	0	0	1	1
	45	w				26	1	1	1	1	1
	45	w				36	1	1	0	1	1
	45	w				41	1	1	1	1	2
2383.	49	w	27	4	2	25	1	1	0	1	1
	49	w				26	1	0	1	1	1
	49	w				27	0	1	1	99	99
	49	w				37	1	0	0	0	0
	49	w				46	1	0	0	0	1
2388.	73	w	9	1	1	45	1	1	0	1	1
2389.	48	w	23	2	6	17	0	1	1	99	99
	48	w				13	1	1	1	1	1
	48	w				11	0	1	1	99	99
	48	w				34	0	1	1	99	99
	48	w				45	0	1	1	99	99
	48	w				46	1	1	1	1	1
2395.	22	m	26	0	1	25	0	1	1	99	99
2396.	71	w	26	4	5	15	1	1	1	1	1
	71	w				24	1	1	0	1	1
	71	w				26	0	1	0	99	99
	71	w				37	1	1	0	1	1
	71	w				47	1	1	1	1	1
2401.	50	m	25	3	4	14	1	0	1	0	0
	50	m				16	0	1	1	99	99
	50	m				12	0	1	1	99	99
	50	m				27	1	1	1	1	1
	50	m				47	1	1	1	1	1
2406.	52	w	28	0	1	47	0	1	1	99	99
2407.	57	m	24	1	0	24	1	0	0	1	1
2408.	63	w	24	0	1	36	0	1	99	99	99
2409.	56	w	28	2	1	16	1	0	0	1	0
	56	w				36	1	1	1	1	1
2411.	29	w	28	1	2	37	0	1	1	99	99
	29	w				36	1	1	1	1	0
2413.	47	m	27	2	2	45	1	1	1	1	1
	47	m				46	1	1	0	0	1
2415.	76	m	18	1	1	23	1	1	0	0	1
2416.	60	w	6	0	2	32	0	1	1	99	99
	60	w				43	0	1	1	99	99
2418.	34	m	28	1	1	46	1	1	1	0	0
2419.	42	w	28	1	2	46	0	1	1	99	99
	42	w				16	1	1	0	1	1
2421.	53	m	25	3	2	16	1	1	0	1	1
	53	m				27	1	0	0	0	2
	53	m				37	1	1	1	1	1
2424.	84	w	17	4	7	12	0	1	1	99	99
	84	w				11	0	1	1	99	99
	84	w				21	1	0	0	1	0
	84	w				22	0	1	1	99	99
	84	w				23	0	0	1	99	99
	84	w				37	0	1	1	99	99
	84	w				36	1	1	0	2	1
	84	w				45	1	1	0	1	1
	84	w				47	0	1	1	99	99
	84	w				46	1	0	1	1	1
2434.	56	w	23	6	3	13	1	1	0	1	1
	56	w				11	1	0	0	0	1
	56	w				21	1	0	0	0	1
	56	w				23	1	0	1	1	0
	56	w				24	1	1	1	1	1
	56	w				27	1	1	1	1	1
2440.	81	w	20	2	1	15	1	1	0	0	1
	81	w				45	1	0	0	0	1
2442.	50	m	28	1	1	26	1	1	0	1	99
2443.	22	m	25	2	4	17	0	1	1	99	99
	22	m				16	0	1	1	99	99
	22	m				37	1	1	1	1	0
	22	m				46	1	1	1	1	1
2447.	63	w	10	2	2	32	1	1	1	0	1
	63	w				31	1	1	1	1	1

2449.	48	m	27	3	3	15	1	1	1	0	1
	48	m				26	1	1	1	1	0
	48	m				36	1	1	1	1	1
2452.	53	m	18	5	12	15	1	1	1	0	0
	53	m				13	0	1	1	99	99
	53	m				14	0	1	1	99	99
	53	m				24	0	1	1	99	99
	53	m				27	1	1	0	1	1
	53	m				37	0	1	1	99	99
	53	m				36	1	1	1	0	1
	53	m				35	1	1	1	1	1
	53	m				34	0	1	1	99	99
	53	m				33	0	1	1	99	99
	53	m				45	0	1	1	99	99
	53	m				47	1	1	1	1	1
2464.	69	w	14	1	3	13	1	1	1	0	1
	69	w				12	0	1	0	99	99
	69	w				23	0	1	0	99	99
2467.	54	w	21	6	3	16	1	1	1	1	1
	54	w				14	1	1	1	1	1
	54	w				12	1	99	99	99	99
	54	w				23	1	0	1	1	1
	54	w				37	1	1	0	0	1
	54	w				45	1	0	1	1	0
2473.	45	w	24	10	3	17	1	1	1	1	1
	45	w				16	0	1	0	99	99
	45	w				14	1	0	0	1	0
	45	w				13	1	0	1	1	1
	45	w				12	1	0	1	0	0
	45	w				11	1	0	0	0	0
	45	w				21	1	0	1	1	1
	45	w				24	1	1	0	1	1
	45	w				26	1	0	1	1	1
	45	w				35	1	0	1	0	1
	45	w				44	0	0	1	99	99
	45	w				34	1	0	0	0	0
2485.	59	w	11	1	1	34	1	1	1	0	1
2486.	36	w	27	1	2	26	0	1	1	99	99
	36	w				36	1	1	0	1	2
2488.	57	w	27	6	5	12	0	1	1	99	99
	57	w				23	1	0	0	0	0
	57	w				37	1	1	0	1	0
	57	w				36	1	1	1	1	1
	57	w				34	1	0	1	1	0
	57	w				45	1	1	1	1	1
	57	w				46	1	1	1	1	1
2495.	75	w	21	2	3	25	1	1	1	1	1
	75	w				27	0	1	0	99	99
	75	w				35	1	1	1	0	1
2498.	62	m	18	5	7	17	1	1	1	1	1
	62	m				16	1	1	0	1	1
	62	m				15	1	1	0	0	1
	62	m				27	1	1	1	0	1
	62	m				35	0	1	1	99	99
	62	m				34	1	1	1	0	1
	62	m				44	0	1	0	99	99
2505.	44	m	25	4	3	16	1	1	0	1	1
	44	m				24	1	0	0	0	1
	44	m				26	1	0	1	1	0
	44	m				37	0	1	0	99	99
	44	m				46	1	1	1	0	0
2510.	30	w	26	1	0	46	1	0	1	1	1
2511.	21	m	28	0	1	26	0	1	1	99	99
2512.	52	m	28	1	0	36	1	0	0	1	0
2513.	33	m	28	3	3	16	1	1	1	0	1
	33	m				26	0	1	1	99	99
	33	m				35	1	0	0	0	0
	33	m				46	1	1	1	0	1
2517.	61	w	14	6	6	15	1	1	1	0	1
	61	w				13	1	1	0	1	1
	61	w				12	1	1	0	1	1
	61	w				23	1	1	0	0	1
	61	w				24	1	1	1	0	1
	61	w				33	0	1	1	99	99
	61	w				44	1	0	1	0	1
2524.	42	w	28	1	2	17	0	1	1	99	99
	42	w				25	1	1	0	1	99

2526.	72	w	7	2	0	26	1	0	0	1	1
	72	w				43	1	0	0	1	2
2528.	65	m	22	4	3	12	1	1	0	1	0
	65	m				11	1	1	0	0	1
	65	m				25	1	0	0	1	99
	65	m				41	1	1	0	1	1
2532.	58	m	9	1	1	26	0	1	1	99	99
	58	m				43	1	0	0	0	2
2534.	51	m	27	1	2	27	1	1	0	1	1
	51	m				37	0	1	1	99	99
2536.	56	w	26	5	5	16	0	1	1	99	99
	56	w				15	1	1	1	1	0
	56	w				14	1	1	0	0	1
	56	w				22	1	1	1	1	1
	56	w				45	1	0	1	0	1
	56	w				46	1	1	1	1	0
2542.	77	w	11	2	1	12	1	1	1	1	0
	77	w				25	1	0	0	0	1
2544.	26	w	26	1	2	21	1	0	0	0	0
	26	w				27	0	1	1	99	99
	26	w				36	0	1	1	99	99
2547.	45	m	19	1	6	14	1	1	0	1	1
	45	m				17	0	1	1	99	99
	45	m				36	0	1	1	99	99
	45	m				42	0	1	1	99	99
	45	m				46	0	1	1	99	99
	45	m				47	0	1	1	99	99
2553.	66	m	24	4	6	17	1	1	1	1	1
	66	m				16	1	1	0	2	1
	66	m				15	0	1	1	99	99
	66	m				24	1	1	1	1	1
	66	m				26	0	1	1	99	99
	66	m				47	1	1	0	1	1
2559.	63	w	20	6	3	15	1	0	0	2	1
	63	w				14	1	0	0	2	1
	63	w				13	1	1	1	0	0
	63	w				22	1	1	1	0	1
	63	w				24	1	0	0	0	1
	63	w				35	1	0	1	0	1
	63	w				45	0	1	1	99	99
2566.	22	m	27	1	1	15	0	1	1	99	99
	22	m				25	1	0	0	0	0
2568.	58	m	25	1	2	26	0	1	1	99	99
	58	m				27	1	1	1	1	1
2570.	64	m	19	7	6	15	1	0	0	0	99
	64	m				11	1	1	1	0	1
	64	m				21	1	1	0	1	0
	64	m				24	1	1	0	1	1
	64	m				25	1	1	1	0	1
	64	m				36	0	1	1	99	99
	64	m				45	1	0	0	1	1
	64	m				46	1	1	1	1	0
2578.	34	w	22	3	4	22	0	1	0	99	99
	34	w				24	1	1	1	1	1
	34	w				25	1	0	1	1	1
	34	w				15	0	1	1	99	99
	34	w				26	1	0	0	1	1
	34	w				35	0	1	1	99	99
2584.	40	w	26	4	4	15	1	0	1	1	1
	40	w				13	1	1	1	1	1
	40	w				37	1	1	1	0	1
	40	w				36	1	0	0	1	0
	40	w				34	0	1	0	99	99
	40	w				32	0	1	0	99	99
2590.	57	w	24	5	2	17	1	1	0	1	1
	57	w				14	1	0	1	0	1
	57	w				24	1	0	0	0	1
	57	w				35	1	0	0	0	0
	57	w				47	1	1	0	2	1
2595.	53	m	20	5	6	16	1	1	1	1	1
	53	m				15	1	1	1	1	1
	53	m				23	1	1	1	0	2
	53	m				26	0	1	1	99	99
	53	m				27	1	1	1	1	1
	53	m				35	1	1	1	0	1
2601.	25	m	28	3	3	16	1	1	0	1	1
	25	m				15	0	1	1	99	99

	25	m				25	1	1	0	1	1
	25	m				46	1	0	0	1	1
2605.	67	m	27	3	2	14	1	1	0	1	1
	67	m				24	1	0	1	1	1
	67	m				36	1	1	1	0	1
2608.	42	w	23	3	3	17	1	1	1	1	1
	42	w				16	1	1	1	1	1
	42	w				15	0	0	1	99	99
	42	w				25	1	1	1	1	1
2612.	76	m	25	1	2	27	0	1	0	99	99
	76	m				35	1	1	1	0	1
2614.	72	w	27	5	3	16	1	1	1	0	1
	72	w				14	1	0	0	0	1
	72	w				13	1	0	1	0	1
	72	w				23	0	1	1	99	99
	72	w				37	1	0	1	1	1
	72	w				45	1	1	1	0	1
2620.	43	m	25	4	5	16	1	1	0	1	1
	43	m				15	1	1	1	1	0
	43	m				22	0	1	0	99	99
	43	m				24	1	1	0	1	1
	43	m				27	1	1	0	1	1
2625.	71	w	3	1	1	42	0	0	1	99	99
	71	w				43	1	1	0	1	1
2627.	54	m	19	1	2	35	0	1	0	99	99
	54	m				45	1	1	0	1	1
2629.	65	w	5	1	3	34	1	1	1	0	1
	65	w				33	0	1	1	99	99
	65	w				41	0	1	1	99	99
2632.	26	w	28	0	3	17	0	1	1	99	99
	26	w				27	0	1	1	99	99
	26	w				47	0	1	1	99	99
2635.	54	w	27	4	3	17	1	1	1	1	1
	54	w				16	1	1	1	1	1
	54	w				14	1	0	1	0	0
	54	w				21	1	1	1	1	0
2639.	62	w	20	4	3	21	1	1	0	0	0
	62	w				23	1	1	0	0	1
	62	w				36	1	1	1	1	1
	62	w				45	1	0	1	1	99
2643.	53	m	25	1	1	15	0	1	1	99	99
	53	m				24	1	0	1	1	1
2645.	63	m	21	2	5	14	1	1	1	1	1
	63	m				24	0	1	0	99	99
	63	m				25	1	1	0	0	0
	63	m				33	0	1	1	99	99
	63	m				47	0	1	1	99	99
2650.	48	m	25	5	4	16	1	1	1	1	1
	48	m				15	1	1	1	0	1
	48	m				14	1	1	1	1	1
	48	m				22	1	1	1	1	1
	48	m				35	1	0	1	1	1
2655.	26	w	28	2	0	21	1	0	0	1	1
	26	w				22	1	0	1	1	0
2657.	27	w	23	7	8	17	0	1	1	99	99
	27	w				16	1	0	1	1	1
	27	w				13	1	1	0	0	99
	27	w				12	1	1	0	0	1
	27	w				11	1	1	0	0	1
	27	w				22	1	1	0	1	1
	27	w				23	1	1	0	0	1
	27	w				25	1	0	1	0	1
	27	w				36	0	1	1	99	99
	27	w				35	0	0	1	99	99
	27	w				46	0	1	0	99	99
2668.	58	w	18	7	3	13	1	1	99	99	1
	58	w				11	1	0	1	99	0
	58	w				21	1	99	1	1	99
	58	w				23	1	1	99	1	1
	58	w				36	1	1	0	0	0
	58	w				33	1	0	1	1	2
	58	w				44	1	0	0	99	1
2675.	46	w	24	4	3	17	0	1	1	99	99
	46	w				16	1	1	0	1	1
	46	w				15	0	1	1	99	99
	46	w				24	1	0	1	1	1
	46	w				26	1	0	0	1	1

	46	w				27	1	0	0	1	1
2681.	62	m	6	2	2	33	1	1	1	1	1
	62	m				44	1	1	1	1	1
2683.	76	m	9	3	3	13	1	1	1	0	1
	76	m				22	1	1	1	1	1
	76	m				23	1	1	1	1	0
2686.	60	w	24	1	1	13	1	1	0	1	1
2687.	66	m	24	4	4	16	0	1	99	99	99
	66	m				15	1	1	0	1	1
	66	m				12	1	1	0	0	1
	66	m				24	1	1	0	1	1
	66	m				35	1	0	0	2	1
2692.	64	w	19	10	8	16	1	1	1	1	1
	64	w				15	1	0	1	0	0
	64	w				14	1	1	1	0	1
	64	w				12	1	1	1	1	1
	64	w				21	1	1	0	1	1
	64	w				22	1	0	1	2	1
	64	w				23	1	1	0	1	1
	64	w				24	1	1	99	1	1
	64	w				25	1	0	0	1	0
	64	w				26	0	1	0	99	99
	64	w				46	1	1	1	1	1
2703.	43	w	15	2	1	26	0	1	1	99	99
	43	w				33	1	0	1	0	1
	43	w				43	1	0	1	0	1
2706.	48	w	26	4	5	15	0	1	0	99	99
	48	w				14	1	1	1	0	1
	48	w				11	1	0	1	1	1
	48	w				26	1	1	0	1	1
	48	w				46	0	1	1	99	99
	48	w				47	1	1	0	0	1
2712.	56	w	12	1	3	11	1	1	0	0	1
	56	w				23	0	1	1	99	99
	56	w				27	0	1	1	99	99
2715.	74	w	23	9	7	15	1	1	1	1	1
	74	w				14	0	1	1	99	99
	74	w				23	1	1	0	1	0
	74	w				24	1	1	1	1	0
	74	w				36	1	0	1	1	0
	74	w				35	1	0	0	0	0
	74	w				44	1	1	0	1	1
	74	w				45	1	1	0	0	2
	74	w				46	1	1	1	2	1
	74	w				47	1	0	1	1	1
2725.	45	m	21	5	2	23	1	1	0	0	0
	45	m				24	1	0	0	1	1
	45	m				35	1	0	0	0	0
	45	m				34	1	0	1	0	1
	45	m				46	0	1	1	99	99
	45	m				47	1	0	0	0	1
2731.	45	m	27	4	4	15	1	0	1	0	0
	45	m				24	1	1	0	1	2
	45	m				25	1	1	0	1	0
	45	m				26	1	1	0	1	1
	45	m				36	0	1	1	99	99
2736.	59	m	26	3	3	12	1	1	0	1	1
	59	m				26	0	1	1	99	99
	59	m				36	1	1	1	1	1
	59	m				44	1	0	0	1	1
2740.	35	m	26	3	1	11	1	0	0	1	1
	35	m				35	1	1	0	0	0
	35	m				34	1	0	0	0	0
2743.	46	w	22	3	3	14	1	1	0	1	0
	46	w				13	0	0	1	99	99
	46	w				25	1	1	0	0	0
	46	w				35	1	1	0	0	1
2747.	57	w	20	0	2	17	0	1	99	99	99
	57	w				46	0	1	1	99	99
2749.	60	m	27	1	2	21	0	1	1	99	99
	60	m				26	1	1	0	1	1
2751.	26	m	28	5	7	17	0	1	1	99	99
	26	m				16	1	1	1	1	1
	26	m				13	0	1	0	99	99
	26	m				23	1	1	0	1	1
	26	m				25	1	1	1	1	1
	26	m				26	1	1	0	1	1

	26	m				36	1	1	1	1	1
2758.	49	w	22	2	3	36	1	1	1	0	2
	49	w				16	0	1	1	99	99
	49	w				46	1	1	1	1	1
2761.	26	w	28	0	1	26	0	1	1	99	99
2762.	50	w	27	3	2	15	1	1	0	0	1
	50	w				25	1	1	0	1	1
	50	w				45	1	0	1	1	1
2765.	47	w	27	1	1	26	1	0	0	1	1
	47	w				36	0	1	1	99	99
2767.	58	w	5	4	2	33	1	0	0	1	1
	58	w				41	1	1	1	1	1
	58	w				42	1	0	1	1	99
	58	w				43	1	1	1	1	1
2771.	62	m	13	1	1	27	1	1	1	1	1
2772.	85	w	24	2	2	24	1	1	1	1	1
	85	w				36	1	1	0	1	1
2774.	60	w	17	3	1	12	1	1	1	0	1
	60	w				35	1	0	0	0	1
	60	w				26	1	99	0	0	99
2777.	61	w	28	2	1	37	1	1	0	1	1
	61	w				35	1	0	0	1	1
2779.	54	m	26	4	3	26	1	1	1	1	1
	54	m				27	1	1	1	1	1
	54	m				46	1	0	0	1	1
	54	m				47	1	1	1	1	1
2783.	58	w	24	3	3	15	1	1	0	1	1
	58	w				45	1	1	0	0	1
	58	w				46	1	1	1	0	1
2786.	43	w	24	3	3	16	0	1	1	99	99
	43	w				15	1	0	0	1	1
	43	w				27	1	1	0	1	1
	43	w				47	1	1	1	1	0
2790.	54	w	22	2	2	22	1	1	0	1	1
	54	w				27	1	1	0	1	1
2792.	91	m	6	1	1	44	1	1	1	0	1
2793.	22	m	28	1	3	26	0	1	1	99	99
	22	m				36	0	1	1	99	99
	22	m				46	1	1	1	1	0
2796.	76	w	6	2	2	21	1	1	0	99	99
	76	w				22	1	1	0	1	1
2798.	73	m	14	0	2	23	0	1	1	99	99
	73	m				36	0	1	1	99	99
2800.	52	w	24	2	3	16	1	0	0	1	1
	52	w				15	1	0	1	0	1
	52	w				26	0	1	1	99	99
	52	w				27	0	1	1	99	99
	52	w				47	0	1	1	99	99
2805.	29	w	24	1	0	15	1	0	1	1	1
2806.	62	m	25	5	3	15	1	1	1	1	1
	62	m				24	1	0	0	0	0
	62	m				37	1	1	1	0	2
	62	m				36	1	0	0	0	1
	62	m				45	1	1	1	0	0
2811.	41	m	24	3	7	17	0	1	1	99	99
	41	m				16	1	1	1	1	1
	41	m				15	1	1	1	0	1
	41	m				26	1	1	1	1	1
	41	m				27	0	1	1	99	99
	41	m				36	0	1	1	99	99
	41	m				35	0	1	0	99	99
2818.	23	m	28	3	3	15	0	1	1	99	99
	23	m				26	1	1	1	1	1
	23	m				45	1	0	0	0	1
	23	m				46	1	1	1	1	1
2822.	28	m	28	3	1	37	1	1	1	0	2
	28	m				31	1	0	0	0	1
	28	m				41	1	0	1	0	1
2825.	27	m	28	1	2	25	1	1	1	0	1
	27	m				26	0	1	1	99	99
2827.	63	w	16	12	8	16	0	1	1	99	99
	63	w				14	1	1	1	1	0
	63	w				11	1	99	1	1	2
	63	w				21	1	1	1	1	1
	63	w				23	1	0	1	0	1
	63	w				24	1	1	0	1	1
	63	w				33	1	1	1	0	0

	63	w				32	1	0	1	1	1
	63	w				31	1	1	1	1	1
	63	w				41	1	0	0	1	1
	63	w				42	1	1	99	99	99
	63	w				43	1	0	99	99	99
	63	w				44	1	1	99	99	99
2840.	59	w	22	9	7	15	1	1	1	1	2
	59	w				14	1	1	1	1	1
	59	w				12	1	0	0	99	1
	59	w				22	1	1	0	1	0
	59	w				23	1	99	1	1	99
	59	w				26	1	1	1	1	1
	59	w				36	1	1	0	0	2
	59	w				35	1	1	1	1	1
	59	w				46	1	1	0	1	1
2849.	50	m	24	8	7	17	0	1	1	99	99
	50	m				16	0	1	1	99	99
	50	m				15	1	1	1	1	1
	50	m				14	1	1	1	0	1
	50	m				11	1	1	0	1	1
	50	m				23	1	1	0	1	0
	50	m				26	1	1	0	1	1
	50	m				37	1	0	0	0	1
	50	m				35	1	0	1	1	1
	50	m				45	1	0	0	0	0
2859.	52	w	18	2	2	16	1	1	0	1	1
	52	w				14	1	1	1	1	1
2861.	41	w	28	5	3	15	1	0	0	1	1
	41	w				14	1	0	0	1	1
	41	w				27	1	1	1	1	0
	41	w				37	1	1	1	1	1
	41	w				36	0	1	1	99	99
	41	w				35	1	0	0	1	1
2867.	49	m	28	1	0	45	1	0	0	0	1
2868.	69	w	17	1	0	25	1	0	0	1	1
2869.	62	m	7	2	3	13	1	1	0	0	1
	62	m				23	1	1	1	0	1
	62	m				45	0	1	1	99	99
2872.	66	m	17	1	3	16	0	1	1	99	99
	66	m				11	1	1	0	1	1
	66	m				46	0	1	1	99	99
2875.	29	w	28	2	1	14	1	1	99	1	99
	29	w				25	1	0	1	1	1
2877.	61	m	27	11	10	15	1	1	1	1	1
	61	m				12	1	1	1	1	1
	61	m				24	1	1	0	1	1
	61	m				25	1	1	0	2	1
	61	m				26	1	1	0	1	1
	61	m				27	1	1	1	1	1
	61	m				37	1	1	1	1	1
	61	m				36	1	1	1	1	1
	61	m				35	1	0	0	1	1
	61	m				42	1	1	1	1	1
	61	m				46	1	1	0	1	2
2888.	37	m	26	3	2	25	1	1	0	0	1
	37	m				26	1	1	1	1	1
	37	m				36	1	0	0	0	1
2891.	64	m	25	2	2	17	1	1	0	0	1
	64	m				16	1	1	0	1	1
2893.	70	m	17	11	11	15	1	1	0	1	1
	70	m				13	1	1	1	1	1
	70	m				12	1	1	0	1	1
	70	m				23	1	1	1	1	1
	70	m				25	1	1	1	1	0
	70	m				26	1	1	1	1	1
	70	m				34	1	1	1	0	1
	70	m				33	1	1	0	0	1
	70	m				32	1	0	0	0	1
	70	m				41	0	1	1	99	99
	70	m				42	1	1	1	1	1
	70	m				44	1	1	0	1	1
2905.	47	m	28	0	99	17	99	99	99	99	99
2906.	36	w	28	0	99	17	99	99	99	99	99
2907.	53	w	15	0	99	17	99	99	99	99	99
2908.	31	w	28	0	99	17	99	99	99	99	99
2909.	27	m	28	0	99	17	99	99	99	99	99
2910.	34	m	27	0	99	17	99	99	99	99	99

2911.	26	m	28	0	99	17	99	99	99	99	99
2912.	68	w	8	0	99	17	99	99	99	99	99
2913.	52	m	28	0	99	17	99	99	99	99	99
2914.	53	m	21	0	99	17	99	99	99	99	99
2915.	24	m	28	0	99	17	99	99	99	99	99
2916.	22	w	28	0	99	17	99	99	99	99	99
2917.	22	m	26	0	99	17	99	99	99	99	99
2918.	23	w	28	0	99	17	99	99	99	99	99
2919.	31	m	28	0	99	17	99	99	99	99	99
2920.	56	w	8	0	99	17	99	99	99	99	99
2921.	25	m	28	0	99	17	99	99	99	99	99
2922.	27	m	27	0	99	17	99	99	99	99	99
2923.	35	m	28	0	99	17	99	99	99	99	99
2924.	34	m	27	0	99	17	99	99	99	99	99
2925.	22	m	28	0	99	17	99	99	99	99	99
2926.	29	m	28	0	99	17	99	99	99	99	99
2927.	24	w	26	0	99	17	99	99	99	99	99
2928.	25	w	28	0	99	17	99	99	99	99	99
2929.	37	m	28	0	99	17	99	99	99	99	99
2930.	22	m	28	0	99	17	99	99	99	99	99
2931.	41	w	27	0	99	17	99	99	99	99	99
2932.	21	m	28	0	99	17	99	99	99	99	99
2933.	25	m	28	0	99	17	99	99	99	99	99
2934.	69	m	6	0	99	17	99	99	99	99	99
2935.	28	m	28	0	99	17	99	99	99	99	99
2936.	51	w	28	0	99	17	99	99	99	99	99
2937.	45	m	26	0	99	17	99	99	99	99	99
2938.	20	m	27	0	99	17	99	99	99	99	99
2939.	70	w	9	0	99	17	99	99	99	99	99
2940.	25	m	28	0	99	17	99	99	99	99	99
2941.	24	m	28	0	99	17	99	99	99	99	99
2942.	49	w	16	0	99	17	99	99	99	99	99
2943.	72	m	15	0	99	17	99	99	99	99	99
2944.	49	w	22	0	99	17	99	99	99	99	99
2945.	53	m	13	0	99	17	99	99	99	99	99
2946.	22	w	26	0	99	17	99	99	99	99	99
2947.	28	w	28	0	99	17	99	99	99	99	99
2949.	37	m	28	0	99	17	99	99	99	99	99
2950.	28	m	24	0	99	17	99	99	99	99	99
2951.	21	w	28	0	99	17	99	99	99	99	99
2952.	24	m	28	0	99	17	99	99	99	99	99
2953.	65	m	10	0	99	17	99	99	99	99	99
2954.	65	m	12	0	99	17	99	99	99	99	99
2955.	55	w	26	0	99	17	99	99	99	99	99
2956.	37	m	28	0	99	17	99	99	99	99	99
2957.	41	w	28	0	99	17	99	99	99	99	99
2958.	33	w	26	0	99	17	99	99	99	99	99
2959.	28	w	28	0	99	17	99	99	99	99	99
2960.	54	w	11	0	99	17	99	99	99	99	99
2961.	26	w	26	0	99	17	99	99	99	99	99
2962.	25	w	28	0	99	17	99	99	99	99	99
2963.	24	w	28	0	99	17	99	99	99	99	99
2964.	22	m	28	0	99	17	99	99	99	99	99
2965.	25	w	28	0	99	17	99	99	99	99	99
2966.	21	m	28	0	99	17	99	99	99	99	99
2967.	31	w	28	0	99	17	99	99	99	99	99
2968.	24	m	28	0	99	17	99	99	99	99	99
2969.	76	m	20	0	99	17	99	99	99	99	99
2970.	22	m	28	0	99	17	99	99	99	99	99
2971.	40	w	28	0	99	17	99	99	99	99	99
2972.	68	w	21	0	99	17	99	99	99	99	99
2973.	60	m	24	0	99	17	99	99	99	99	99
2974.	21	m	28	0	99	17	99	99	99	99	99
2975.	35	w	28	0	99	17	99	99	99	99	99
2976.	22	m	28	0	99	17	99	99	99	99	99
2977.	63	m	26	0	99	17	99	99	99	99	99
2978.	39	m	28	0	99	17	99	99	99	99	99
2979.	44	w	28	0	99	17	99	99	99	99	99
2980.	33	w	17	0	99	17	99	99	99	99	99
2981.	26	w	25	0	99	17	99	99	99	99	99
2982.	52	w	26	0	99	17	99	99	99	99	99
2983.	19	w	28	0	99	17	99	99	99	99	99
2984.	35	w	28	0	99	17	99	99	99	99	99
2985.	37	w	26	0	99	17	99	99	99	99	99
2986.	29	w	28	0	99	17	99	99	99	99	99
2987.	75	w	3	0	99	17	99	99	99	99	99
2988.	75	w	28	0	99	17	99	99	99	99	99

2989.	54	w	26	0	99	17	99	99	99	99	99
2990.	20	w	28	0	99	17	99	99	99	99	99
2991.	31	m	28	0	99	17	99	99	99	99	99
2992.	21	m	28	0	99	17	99	99	99	99	99
2993.	46	w	27	0	99	17	99	99	99	99	99
2994.	26	w	28	0	99	17	99	99	99	99	99
2995.	45	w	28	0	99	17	99	99	99	99	99
2996.	72	m	27	0	99	17	99	99	99	99	99
2997.	42	w	26	0	99	17	99	99	99	99	99
2998.	22	m	28	0	99	17	99	99	99	99	99
2999.	24	m	28	0	99	17	99	99	99	99	99
3000.	64	m	22	0	99	17	99	99	99	99	99
3001.	74	m	26	0	99	17	99	99	99	99	99
3002.	22	m	28	0	99	17	99	99	99	99	99
3003.	29	w	28	0	99	17	99	99	99	99	99
3004.	31	w	28	0	99	17	99	99	99	99	99
3005.	26	w	28	0	99	17	99	99	99	99	99
3006.	24	m	28	0	99	17	99	99	99	99	99
3007.	23	w	28	0	99	17	99	99	99	99	99
3008.	48	w	27	0	99	17	99	99	99	99	99
3009.	58	m	13	0	99	17	99	99	99	99	99
3010.	25	m	28	0	99	17	99	99	99	99	99
3011.	37	m	25	0	99	17	99	99	99	99	99
3012.	32	m	28	0	99	17	99	99	99	99	99
3013.	68	m	12	0	99	17	99	99	99	99	99
3014.	24	w	28	0	99	17	99	99	99	99	99
3015.	70	m	9	0	99	17	99	99	99	99	99
3016.	26	m	28	0	99	17	99	99	99	99	99
3017.	54	w	12	0	99	17	99	99	99	99	99
3018.	26	w	28	0	99	17	99	99	99	99	99
3019.	55	w	26	0	99	17	99	99	99	99	99
3020.	57	m	27	0	99	17	99	99	99	99	99
3021.	21	m	28	0	99	17	99	99	99	99	99
3022.	49	w	28	0	99	17	99	99	99	99	99
3023.	47	m	24	0	99	17	99	99	99	99	99
3024.	24	w	26	0	99	17	99	99	99	99	99
3025.	21	w	28	0	99	17	99	99	99	99	99
3026.	76	w	18	0	99	17	99	99	99	99	99
3027.	44	w	11	0	99	17	99	99	99	99	99
3028.	67	m	28	0	99	17	99	99	99	99	99
3029.	19	w	28	0	99	17	99	99	99	99	99
3030.	38	m	12	0	99	17	99	99	99	99	99
3031.	32	m	28	0	99	17	99	99	99	99	99
3032.	46	m	27	0	99	17	99	99	99	99	99
3033.	72	w	25	0	99	17	99	99	99	99	99
3034.	22	w	28	0	99	17	99	99	99	99	99
3035.	21	w	28	0	99	17	99	99	99	99	99
3036.	25	w	28	0	99	17	99	99	99	99	99
3037.	71	w	25	0	99	17	99	99	99	99	99
3038.	43	m	24	0	99	17	99	99	99	99	99
3039.	51	m	18	0	99	17	99	99	99	99	99
3040.	28	w	27	0	99	17	99	99	99	99	99
3041.	27	m	28	0	99	17	99	99	99	99	99
3042.	22	w	28	0	99	17	99	99	99	99	99
3043.	48	w	26	0	99	17	99	99	99	99	99
3044.	24	m	28	0	99	17	99	99	99	99	99
3045.	26	w	28	0	99	17	99	99	99	99	99
3046.	36	w	28	0	99	17	99	99	99	99	99
3047.	25	w	28	0	99	17	99	99	99	99	99
3048.	41	w	28	0	99	17	99	99	99	99	99
3049.	20	w	28	0	99	17	99	99	99	99	99
3050.	69	w	10	0	99	17	99	99	99	99	99
3051.	31	w	26	0	99	17	99	99	99	99	99
3052.	31	w	27	0	99	17	99	99	99	99	99
3053.	19	m	28	0	99	17	99	99	99	99	99
3054.	37	m	28	0	99	17	99	99	99	99	99
3055.	26	w	28	0	99	17	99	99	99	99	99
3056.	36	m	28	0	99	17	99	99	99	99	99
3057.	19	w	28	0	99	17	99	99	99	99	99
3058.	45	m	28	0	99	17	99	99	99	99	99
3059.	19	w	28	0	99	17	99	99	99	99	99
3060.	39	w	24	0	99	17	99	99	99	99	99
3061.	50	w	24	0	99	17	99	99	99	99	99
3062.	22	w	28	0	99	17	99	99	99	99	99
3063.	26	w	28	0	99	17	99	99	99	99	99
3064.	70	w	22	0	99	17	99	99	99	99	99
3065.	24	m	28	0	99	17	99	99	99	99	99

3066.	78	m	17	0	99	17	99	99	99	99	99
3067.	24	w	28	0	99	17	99	99	99	99	99
3068.	23	w	28	0	99	17	99	99	99	99	99
3069.	42	w	26	0	99	17	99	99	99	99	99
3070.	53	w	28	0	99	17	99	99	99	99	99
3071.	22	m	28	0	99	17	99	99	99	99	99
3072.	52	w	28	0	99	17	99	99	99	99	99
3073.	33	w	28	0	99	17	99	99	99	99	99
3074.	57	w	2	0	99	17	99	99	99	99	99
3075.	52	w	25	0	99	17	99	99	99	99	99
3076.	27	m	28	0	99	17	99	99	99	99	99
3077.	24	m	28	0	99	17	99	99	99	99	99
3078.	22	w	28	0	99	17	99	99	99	99	99
3079.	66	m	15	0	99	17	99	99	99	99	99
3080.	70	w	27	0	99	17	99	99	99	99	99
3081.	68	w	13	0	99	17	99	99	99	99	99
3082.	24	w	28	0	99	17	99	99	99	99	99
3083.	81	m	23	0	99	17	99	99	99	99	99
3084.	22	w	28	0	99	17	99	99	99	99	99
3085.	19	m	28	0	99	17	99	99	99	99	99
3086.	25	m	28	0	99	17	99	99	99	99	99
3087.	31	w	28	0	99	17	99	99	99	99	99
3088.	48	m	28	0	99	17	99	99	99	99	99
3089.	79	w	3	0	99	17	99	99	99	99	99
3090.	20	m	27	0	99	17	99	99	99	99	99
3091.	49	m	25	0	99	17	99	99	99	99	99
3092.	19	m	28	0	99	17	99	99	99	99	99
3093.	36	w	24	0	99	17	99	99	99	99	99
3094.	59	w	2	0	99	17	99	99	99	99	99
3095.	63	m	13	0	99	17	99	99	99	99	99
3096.	60	w	17	0	99	17	99	99	99	99	99
3097.	73	w	24	0	99	17	99	99	99	99	99
3098.	20	w	28	0	99	17	99	99	99	99	99
3099.	25	m	28	0	99	17	99	99	99	99	99
3100.	37	m	28	0	99	17	99	99	99	99	99
3101.	53	m	13	0	99	17	99	99	99	99	99
3102.	24	m	28	0	99	17	99	99	99	99	99
3103.	73	m	21	0	99	17	99	99	99	99	99
3104.	24	w	28	0	99	17	99	99	99	99	99
3105.	63	w	27	0	99	17	99	99	99	99	99
3106.	25	m	28	0	99	17	99	99	99	99	99
3107.	29	w	28	0	99	17	99	99	99	99	99
3108.	28	w	28	0	99	17	99	99	99	99	99
3109.	35	w	28	0	99	17	99	99	99	99	99
3110.	55	w	27	0	99	17	99	99	99	99	99
3111.	42	w	27	0	99	17	99	99	99	99	99
3112.	25	m	28	0	99	17	99	99	99	99	99
3114.	22	m	28	0	99	17	99	99	99	99	99
3115.	25	w	28	0	99	17	99	99	99	99	99
3116.	25	w	28	0	99	17	99	99	99	99	99
3117.	22	w	28	0	99	17	99	99	99	99	99
3118.	29	w	28	0	99	17	99	99	99	99	99
3119.	76	m	7	0	99	17	99	99	99	99	99
3120.	67	m	20	0	99	17	99	99	99	99	99
3121.	23	m	28	0	99	17	99	99	99	99	99
3122.	24	m	28	0	99	17	99	99	99	99	99
3123.	32	m	28	0	99	17	99	99	99	99	99
3124.	33	w	28	0	99	17	99	99	99	99	99
3125.	80	m	2	0	99	17	99	99	99	99	99
3126.	22	w	28	0	99	17	99	99	99	99	99
3127.	29	w	25	0	99	17	99	99	99	99	99
3128.	31	m	28	0	99	17	99	99	99	99	99
3129.	32	m	28	0	99	17	99	99	99	99	99
3130.	27	w	28	0	99	17	99	99	99	99	99
3131.	42	w	28	0	99	17	99	99	99	99	99
3132.	24	w	28	0	99	17	99	99	99	99	99
3133.	23	m	28	0	99	17	99	99	99	99	99
3134.	74	w	1	0	99	17	99	99	99	99	99
3135.	24	w	28	0	99	17	99	99	99	99	99
3136.	32	w	28	0	99	17	99	99	99	99	99
3137.	22	w	28	0	99	17	99	99	99	99	99
3138.	23	m	28	0	99	17	99	99	99	99	99
3139.	40	w	28	0	99	17	99	99	99	99	99
3140.	79	m	23	0	99	17	99	99	99	99	99
3141.	26	w	28	0	99	17	99	99	99	99	99
3142.	77	w	9	0	99	17	99	99	99	99	99
3143.	34	m	28	0	99	17	99	99	99	99	99

3144.	23	w	28	0	99	17	99	99	99	99	99
3145.	21	m	28	0	99	17	99	99	99	99	99
3146.	47	m	28	0	99	17	99	99	99	99	99
3147.	26	w	28	0	99	17	99	99	99	99	99
3148.	30	m	28	0	99	17	99	99	99	99	99
3149.	22	m	28	0	99	17	99	99	99	99	99
3150.	39	m	28	0	99	17	99	99	99	99	99
3151.	24	w	28	0	99	17	99	99	99	99	99
3152.	69	m	3	0	99	17	99	99	99	99	99
3153.	31	w	28	0	99	17	99	99	99	99	99
2608.	59	w	24	0	99	17	99	99	99	99	99

QR	PF	RS	posts/Screw	pR
1	0	0	1	2
1	0	0	99	99
1	99	99	0	0
0	0	0	4	2
0	0	0	4	2
0	0	0	99	99
0	99	99	0	0
0	1	0	0	0
1	0	0	1	0
1	1	0	99	99
1	0	0	0	0
0	0	0	4	0
0	0	0	99	99
1	0	0	0	0
1	0	0	4	2
99	99	0	0	2
99	0	0	0	2
0	0	0	4	2
0	1	0	99	99
0	0	0	99	99
0	0	0	99	99
0	0	0	99	99
1	0	0	99	99
1	0	0	0	0
0	0	0	4	2
1	0	0	0	0
1	0	0	0	0
99	0	0	99	99
1	0	0	99	99
1	0	0	0	0
1	0	0	0	0
0	0	0	0	0
0	0	0	0	0
0	0	0	99	99
1	0	0	99	99
1	0	0	1	0
1	0	0	0	0
1	0	0	99	99
1	0	0	99	99
0	0	0	0	0
99	0	0	0	0
99	0	0	0	0
1	0	0	0	0
1	0	0	4	2
0	0	0	4	2
1	0	0	99	99
0	0	0	99	99
1	0	0	99	99
1	1	0	99	99
99	0	0	99	99
0	0	0	0	2
1	0	0	0	0
1	0	0	99	99
1	0	0	0	0
0	0	0	99	99
0	0	0	99	99
1	0	0	0	2
0	0	0	0	2
0	0	0	99	99
0	0	0	99	99
1	0	0	0	0
1	0	0	4	2
1	0	0	99	99
0	0	0	99	99

0	0	0	1	2
0	1	0	4	2
0	0	0	0	2
1	0	0	0	2
0	0	0	1	0
0	0	0	0	2
0	0	0	4	0
0	0	0	99	99
1	0	0	99	99
0	0	0	99	99
0	0	0	99	99
99	0	0	4	0
1	0	0	1	0
0	0	0	4	0
0	0	0	99	99
0	0	0	0	0
0	0	0	0	0
0	0	0	0	2
0	0	1	99	99
1	0	0	0	0
0	0	0	0	0
0	0	0	99	99
1	0	0	0	0
0	0	0	0	0
99	1	0	0	0
99	0	0	99	99
1	0	0	99	99
1	0	0	99	99
0	0	1	99	99
1	0	1	99	99
1	0	0	0	0
1	0	0	2	0
1	0	0	0	0
99	0	0	0	0
0	0	0	0	0
0	0	0	0	0
0	0	0	0	0
0	0	0	1	2
1	0	1	0	2
0	0	0	0	0
0	0	0	99	99
1	0	0	0	0
0	0	0	99	99
0	0	1	99	99
0	0	1	99	99
1	0	0	4	2
0	0	0	99	99
0	0	0	0	0
1	0	0	0	0
0	0	0	0	0
0	0	0	99	99
1	0	0	99	99
0	0	0	99	99
99	0	1	2	0
1	0	0	99	99
99	0	0	0	2
0	0	0	0	2
0	0	0	99	99
1	0	0	0	0
1	0	0	0	0
1	0	0	4	2
0	0	0	1	2
1	1	0	0	0
0	0	0	99	99
1	0	0	0	0
99	0	0	0	0
0	1	0	0	0
1	0	0	0	2
0	0	1	99	99
1	0	0	0	2
1	0	0	0	0
1	1	0	0	0
1	1	0	4	2
99	0	0	4	2
1	0	0	0	0
1	0	0	0	0
1	0	0	0	0

1	0	0	99	99
1	0	0	0	0
0	0	0	99	99
99	0	0	99	99
1	0	0	99	99
0	0	0	99	99
1	0	0	99	99
99	0	0	99	99
1	0	0	0	0
0	0	0	0	2
1	0	0	99	99
1	0	0	0	2
1	0	0	99	99
1	0	0	99	99
0	0	0	99	99
1	0	0	0	2
0	0	0	0	2
0	0	0	99	99
0	0	1	99	99
0	0	0	99	99
1	0	0	2	2
0	0	0	1	2
0	0	0	4	2
0	0	0	4	2
1	0	0	4	2
1	0	0	0	0
0	0	0	0	0
1	0	0	4	2
1	0	0	4	0
99	99	99	99	99
1	0	0	4	2
1	0	0	0	2
1	0	0	99	99
1	0	0	99	99
1	0	0	99	99
1	0	0	99	99
1	99	0	99	99
1	99	0	99	99
1	99	0	0	0
1	99	0	99	99
1	99	0	99	99
1	99	0	99	99
1	99	0	99	99
1	99	0	0	0
1	99	0	0	99
1	0	0	0	99
1	99	0	0	0
1	0	0	0	0
1	0	0	4	0
1	0	0	0	0
1	0	0	0	0
0	0	0	0	0
1	0	0	0	0
0	0	0	0	0
0	0	0	0	0
0	0	0	99	99
0	0	0	0	2
1	1	0	99	99
1	1	0	0	2
0	0	0	0	0
1	0	0	0	0
1	0	0	0	0
1	0	0	0	0
0	0	0	2	2
0	0	0	99	99
0	0	0	99	99
0	0	0	0	2
0	0	0	0	0
0	0	0	0	0
0	1	0	0	0
0	0	0	1	2
0	0	0	4	2
0	0	0	99	99
0	0	0	1	2
0	0	0	4	2
0	99	99	99	99
0	0	99	99	99

0	0	99	99	99
0	0	99	0	0
0	0	0	0	0
1	0	0	0	0
0	0	0	0	0
0	0	0	4	1
1	1	0	99	99
1	0	1	99	99
0	0	0	4	2
99	0	0	4	2
1	0	0	0	2
1	0	0	4	2
1	0	0	0	0
0	0	0	1	2
1	0	0	4	2
0	0	0	99	99
0	0	0	0	0
0	0	0	4	0
0	0	0	4	2
1	0	0	0	0
1	0	0	99	99
1	0	0	99	99
1	0	0	99	99
1	0	0	0	0
1	0	0	1	2
1	1	0	1	2
0	0	0	99	99
0	0	0	99	99
0	0	0	99	99
0	0	0	99	99
99	0	0	1	2
99	99	0	1	2
0	0	0	1	2
0	0	0	4	2
0	0	0	99	99
1	0	0	99	99
1	0	0	0	2
0	0	0	0	2
0	0	0	4	2
1	1	0	0	0
0	1	0	0	0
0	0	0	0	0
1	0	0	1	0
0	0	0	0	0
0	0	0	4	2
0	0	0	2	2
0	0	0	0	0
0	0	0	0	0
99	0	0	0	0
1	0	0	0	0
0	0	0	99	99
0	0	0	0	0
0	0	0	99	99
0	0	0	99	99
0	0	0	99	99
0	0	1	99	99
0	0	0	4	2
0	0	0	4	2
0	0	1	99	99
0	0	0	0	0
1	0	0	99	99
0	0	0	99	99
1	0	0	99	99
0	0	0	0	0
1	0	0	0	2
1	0	0	0	0
1	0	0	4	2
0	1	0	0	2
99	99	0	99	99
99	99	0	99	99
1	1	0	0	2
1	0	0	4	2
1	0	0	99	99
1	0	0	99	99
1	0	0	99	99
0	0	0	4	0
1	0	0	0	0

1	0	0	0	0
1	0	0	99	99
0	0	0	99	99
0	0	0	4	2
1	0	0	0	0
1	0	0	0	0
1	0	0	99	99
1	1	0	0	2
1	0	0	4	2
0	0	0	99	99
1	0	0	99	99
1	0	0	99	99
0	0	0	0	0
0	0	0	0	2
0	0	0	0	2
0	0	0	0	0
1	0	0	0	0
0	0	0	0	0
0	0	0	0	0
0	0	0	99	99
0	0	0	99	99
0	0	0	99	99
0	0	0	99	99
1	0	0	99	99
1	0	0	0	0
0	0	0	0	2
1	0	0	4	2
1	0	0	0	0
0	1	0	0	0
1	0	0	0	0
1	0	0	4	0
0	0	0	99	99
1	0	0	4	0
0	0	0	0	0
1	0	0	0	0
0	0	0	0	0
1	0	0	0	0
1	0	0	4	2
0	0	0	0	2
1	0	0	0	0
1	0	1	99	99
1	0	0	0	2
1	0	0	0	0
0	0	0	0	0
1	0	0	0	0
1	0	0	1	2
1	0	0	4	2
1	0	0	0	2
1	0	0	99	99
0	0	1	99	99
0	0	0	99	99
0	0	0	99	99
1	0	0	4	2
1	0	0	1	2
1	0	0	99	99
99	0	0	0	2
1	0	0	0	2
1	0	0	0	0
1	0	0	0	0
1	0	0	4	2
1	1	0	99	99
1	1	0	0	0
0	0	0	99	99
1	0	0	99	99
0	0	1	99	99
1	0	0	0	1
0	0	0	0	0
1	0	0	0	2
0	1	0	0	0
1	1	0	0	2
99	0	0	0	0
1	1	0	1	0
1	0	0	99	99
1	0	0	99	99
1	0	1	1	0
1	0	0	4	0
0	0	0	99	99

0	0	0	0	0
1	0	0	0	0
1	0	0	99	99
1	0	0	0	0
1	0	0	4	0
1	0	0	0	0
1	0	0	4	0
0	0	1	99	99
1	0	0	4	99
1	1	0	4	0
0	0	0	4	2
1	0	0	4	0
1	0	0	99	99
1	0	0	0	0
99	0	0	4	0
1	0	0	4	2
0	0	0	4	2
1	1	0	0	0
1	1	0	4	99
1	1	0	2	99
0	0	0	0	0
1	0	0	4	2
99	0	0	4	2
1	1	0	4	2
1	0	0	99	99
0	0	0	99	99
0	0	0	4	2
0	0	0	99	99
1	0	0	99	99
1	0	0	0	0
0	0	0	4	2
99	0	0	0	0
0	0	0	4	2
0	0	0	0	2
1	0	0	99	99
99	0	0	0	0
1	0	0	1	2
1	0	0	4	2
1	0	0	4	2
1	0	0	0	2
1	0	0	0	0
99	0	0	0	0
99	99	0	4	2
1	0	0	0	2
1	0	0	1	2
1	0	0	0	0
1	0	0	0	0
1	0	0	4	0
1	0	0	0	0
1	0	0	0	0
1	0	0	0	99
1	0	0	1	2
0	0	0	0	2
99	0	0	0	2
0	0	0	0	0
0	1	0	1	2
1	0	0	4	2
0	0	0	99	99
1	0	0	4	2
0	0	0	0	0
0	0	0	0	2
0	0	0	4	0
0	0	0	0	0
0	0	0	0	0
1	0	0	99	99
1	0	0	2	2
0	0	0	0	0
0	0	0	4	2
1	0	0	4	2
0	0	0	4	2
0	0	0	1	2
1	0	0	4	2
1	0	0	0	0
1	0	0	4	2
0	0	0	0	2
0	1	1	99	99
1	0	0	99	99

1	1	0	0	2
0	0	0	99	99
0	0	0	99	99
1	0	0	0	2
0	99	99	99	99
99	0	0	99	99
0	0	0	0	0
1	0	0	0	0
1	0	0	0	0
1	0	0	99	99
0	0	0	99	99
0	0	1	99	99
1	0	0	4	2
1	0	0	0	2
0	0	0	0	0
99	0	0	1	2
0	0	0	0	2
99	0	0	4	2
99	99	0	0	2
0	0	0	99	99
0	0	0	0	0
99	0	0	0	0
0	0	0	0	0
0	0	0	0	0
1	0	0	0	0
99	0	0	0	0
99	1	0	0	0
0	0	0	99	99
1	0	0	4	2
0	0	0	0	2
1	0	0	1	2
0	0	0	0	2
0	0	0	0	2
1	0	0	0	2
1	0	0	0	0
0	0	0	99	99
0	0	0	4	2
0	0	0	0	0
1	0	0	0	0
0	0	0	0	2
0	0	0	0	2
99	0	0	0	2
1	0	0	0	0
1	0	0	4	0
0	0	0	0	2
0	0	0	0	2
0	0	0	0	0
1	0	0	0	0
0	0	0	0	0
99	0	0	0	0
0	0	1	99	99
0	0	0	99	99
1	0	0	0	2
1	0	0	0	0
0	0	0	4	2
1	0	0	0	2
0	0	0	0	2
1	0	0	0	0
0	1	0	0	2
1	0	0	0	2
0	0	0	0	0
0	0	0	0	0
1	0	0	0	0
1	0	0	0	0
0	0	0	0	0
1	0	0	0	0
1	0	0	0	0
1	0	0	0	99
0	0	0	0	0
1	0	0	99	99
1	0	0	99	99
1	0	0	0	0
0	0	0	0	0
1	0	0	0	99
1	0	0	0	0
0	0	0	0	0
1	0	0	99	99

1	0	0	0	0
1	0	0	0	2
1	1	0	0	0
1	0	0	0	0
1	0	0	0	0
99	0	0	0	0
0	0	0	0	2
1	1	0	0	0
0	0	0	0	0
1	0	0	0	0
0	0	0	0	0
0	0	0	0	0
0	0	0	99	99
0	0	0	99	99
1	0	0	99	99
1	0	0	99	99
0	0	0	0	2
0	0	0	0	2
0	0	0	99	99
0	0	0	99	99
0	0	0	99	99
1	0	0	4	2
1	0	0	99	99
1	0	0	99	99
1	0	0	0	0
1	0	0	0	0
1	0	0	0	0
1	0	1	0	0
1	0	0	2	0
1	0	0	0	0
0	0	0	99	99
0	0	0	0	0
1	0	0	0	0
1	0	0	4	2
0	0	0	0	0
99	99	99	99	99
99	99	99	99	99
99	99	99	99	99
99	99	99	4	2
1	99	0	0	0
1	0	0	0	99
1	0	0	0	0
0	0	0	4	2
0	0	0	0	2
0	0	0	99	99
1	0	0	4	2
0	0	0	99	99
0	0	0	99	99
0	0	0	4	2
0	0	0	0	2
0	0	0	0	2
99	99	0	1	2
99	99	99	99	99
99	99	99	99	99
99	99	99	99	99
99	99	99	4	0
99	0	0	0	0
0	0	0	4	2
99	0	0	4	2
99	0	0	1	2
99	0	0	1	2
99	1	0	1	2
1	0	0	4	0
1	0	0	0	0
1	0	0	1	2
1	0	0	1	2
1	0	0	4	2
99	0	0	4	2
1	0	0	4	2
1	0	0	0	1
0	0	0	99	99
99	0	0	99	99
99	0	0	99	99
99	0	0	0	2
0	0	0	4	2
0	0	0	0	2
1	99	0	99	99

1	0	0	0	0
1	0	0	4	0
1	0	0	0	0
99	0	0	99	99
0	0	0	0	0
0	0	1	99	99
0	0	1	0	0
0	0	0	4	2
0	0	0	0	0
0	0	0	2	0
99	0	0	99	99
1	0	0	99	99
99	0	0	99	99
0	0	0	0	2
0	0	0	0	0
0	1	0	0	0
1	0	0	0	99
1	0	1	99	99
0	1	0	0	0
0	0	0	0	0
0	0	1	99	99
1	0	0	0	0
1	0	0	4	0
0	0	0	4	2
1	0	0	0	2
99	0	1	99	99
1	0	0	0	0
0	0	0	1	2
0	0	0	0	0
1	0	0	4	2
1	0	1	4	2
1	0	0	0	0
1	0	0	0	0
1	0	0	99	99
0	0	0	99	99
1	0	0	0	0
1	0	0	99	99
1	0	0	99	99
0	0	0	0	0
1	0	0	0	0
1	0	0	0	0
0	0	1	99	99
99	0	0	99	99
99	0	0	0	2
0	1	0	0	0
1	0	0	99	99
0	0	0	0	2
0	0	0	0	2
0	0	0	4	2
99	0	0	0	2
1	0	0	0	0
1	0	0	0	0
1	0	0	0	0
1	0	0	4	0
1	0	0	0	0
1	0	0	0	99
1	0	0	0	0
1	1	0	4	2
1	0	0	0	0
1	0	0	0	99
0	0	0	1	2
1	0	0	99	99
1	0	0	99	99
0	0	1	99	99
0	0	1	99	99
1	0	0	99	99
1	0	0	1	2
0	0	0	0	2
0	0	0	0	2
1	0	0	4	2
1	0	0	99	99
1	0	0	99	99
0	0	0	0	0
0	0	0	0	2
99	0	0	0	2
0	0	0	99	99
0	0	0	99	99

99	0	0	0	2
0	0	0	0	2
1	0	0	0	2
0	0	0	0	2
1	0	0	99	99
1	0	0	99	99
1	0	0	1	2
1	0	0	0	2
1	0	0	0	0
0	0	0	0	0
1	0	0	0	0
0	0	0	4	2
0	0	0	0	2
1	0	1	99	99
0	0	1	99	99
1	0	0	99	99
1	0	0	4	2
0	0	0	0	2
0	0	0	0	2
1	0	0	4	2
0	0	0	99	99
0	0	0	99	99
0	0	0	99	99
0	0	0	4	2
0	0	1	99	99
0	0	1	99	99
0	0	1	99	99
0	0	1	99	99
1	0	0	4	2
1	0	0	99	99
99	99	99	99	99
99	99	99	99	99
99	99	99	99	99
1	0	0	0	0
99	0	0	99	99
1	0	0	99	99
0	0	0	99	99
0	0	0	0	0
1	0	0	0	2
0	0	0	0	0
1	0	0	0	0
0	0	0	99	99
0	0	0	99	99
0	0	0	0	2
1	0	0	0	0
0	0	0	0	0
1	0	0	99	99
0	0	0	4	2
0	0	0	4	2
0	0	1	99	99
0	0	0	99	99
1	0	0	99	99
1	0	0	99	99
0	0	0	0	0
1	0	0	99	99
1	0	0	0	2
1	0	0	0	0
0	0	0	99	99
1	0	0	0	1
1	0	0	0	0
1	0	0	0	0
1	0	0	0	0
1	0	0	4	2
0	0	0	0	2
1	0	0	0	0
0	0	0	4	2
0	0	0	1	0
1	0	0	99	99
1	0	0	0	2
0	0	0	0	2
1	0	0	99	99
1	0	0	0	0
0	0	0	99	99
0	0	0	99	99
0	0	1	99	99
0	0	0	99	99
99	0	0	0	0

1	0	0	0	0
1	0	0	0	0
1	0	0	0	0
1	0	0	99	99
0	0	1	99	99
0	0	1	99	99
1	0	0	99	99
1	0	0	0	0
0	0	1	2	2
0	0	1	0	2
0	0	0	0	0
1	0	0	0	0
1	0	0	2	2
1	0	0	4	2
0	0	0	2	2
0	0	0	4	2
0	0	0	0	0
1	0	0	4	0
1	0	0	0	2
1	0	0	99	99
1	0	0	0	0
99	0	0	0	2
0	0	0	4	2
0	0	0	0	0
0	1	0	0	0
1	0	0	0	0
99	0	0	4	2
1	0	0	99	99
1	0	0	99	99
99	0	0	99	99
99	0	0	99	99
99	0	0	99	99
0	0	0	99	99
99	0	0	0	0
99	0	0	0	0
0	0	0	99	99
0	0	0	99	99
0	0	0	0	2
0	0	0	99	99
0	0	0	0	2
1	0	0	0	0
1	0	0	0	0
1	0	0	0	0
1	0	0	0	0
99	0	0	4	2
99	0	0	99	99
1	0	0	0	2
0	0	1	99	99
0	0	0	99	99
1	0	0	99	99
1	0	1	99	99
0	0	0	99	99
0	0	0	0	2
1	1	0	1	2
99	0	0	99	99
0	0	0	0	2
0	0	0	99	99
0	0	0	99	99
0	0	0	99	99
99	0	0	0	2
0	0	0	0	2
1	0	0	0	2
0	0	0	0	2
0	0	0	0	2
99	0	0	99	99
99	0	0	0	2
0	0	0	0	2
1	0	0	4	2
1	0	0	4	2
99	0	0	4	2
1	0	0	0	0
1	0	0	4	2
1	0	0	0	0
1	1	0	99	99
0	0	0	0	0
1	0	0	0	0
0	0	0	99	99

0	0	0	0	0
0	0	0	1	1
1	0	0	99	99
0	0	0	99	99
0	0	0	99	99
99	99	0	99	99
1	0	0	0	2
0	0	1	0	2
1	0	0	0	2
99	0	0	0	2
99	0	0	0	2
1	0	0	99	99
1	0	0	99	99
1	0	0	1	2
99	0	0	0	0
1	0	0	0	2
0	0	0	99	99
0	1	0	99	99
1	0	1	99	99
1	0	0	4	2
99	0	0	0	0
1	0	0	0	0
1	0	0	0	0
1	0	0	4	2
0	0	0	4	2
0	0	0	1	2
1	0	0	4	2
99	0	0	99	99
0	0	0	4	2
0	0	0	99	99
0	0	0	99	99
1	0	0	0	0
1	0	0	0	0
0	0	0	0	0
0	1	0	0	1
1	0	0	0	2
1	0	0	99	99
1	0	0	4	0
0	0	0	0	0
1	0	0	0	0
99	0	0	99	99
99	0	0	99	99
1	99	1	99	99
1	0	0	99	99
1	0	0	4	99
0	0	0	99	99
1	0	0	99	99
0	0	0	0	0
0	0	0	0	0
1	0	0	1	0
0	0	0	0	2
0	0	0	0	2
0	0	0	0	2
0	0	0	99	99
0	0	0	99	99
0	0	0	0	2
0	1	0	0	0
1	0	0	1	2
1	0	1	0	2
99	1	0	0	1
0	0	0	99	99
0	0	0	99	99
0	0	0	0	2
0	0	0	0	2
0	0	0	0	0
1	1	0	0	0
0	0	0	0	0
1	1	0	0	0
99	0	0	99	99
99	0	0	4	2
1	0	0	99	99
1	0	0	99	99
0	0	0	4	2
1	0	0	4	2
1	0	0	0	0
99	0	0	99	99
99	0	0	99	99

99	0	0	0	2
1	1	0	4	0
0	0	0	0	0
1	0	0	2	0
1	0	0	0	0
1	0	0	0	0
1	0	0	0	0
0	0	0	0	0
1	0	0	0	99
0	0	0	0	0
1	0	0	0	2
99	0	0	4	2
1	0	0	0	2
0	0	0	99	99
1	0	0	99	99
0	0	0	99	99
1	0	0	99	99
0	0	1	99	99
0	0	0	99	99
1	0	0	99	99
0	0	0	0	0
0	0	0	0	0
0	0	0	99	99
0	0	0	0	1
0	0	0	4	2
0	0	0	2	2
1	0	0	4	2
99	99	0	4	2
0	0	0	0	2
1	0	0	0	2
99	0	0	99	99
1	0	0	99	99
0	0	0	99	99
0	0	0	0	0
1	0	0	0	0
1	0	0	0	0
1	0	0	0	0
99	0	0	0	0
1	0	0	0	0
0	0	0	0	0
0	0	0	0	0
1	0	0	99	99
1	0	0	4	2
1	0	0	4	2
99	0	1	99	99
1	0	0	0	0
1	0	0	4	2
1	0	0	0	2
1	0	0	0	0
1	0	0	0	0
99	0	0	99	99
1	0	0	0	0
1	0	0	0	0
1	0	0	0	0
1	0	0	0	0
1	0	0	99	99
1	0	0	0	0
0	0	0	99	99
0	0	0	0	0
99	0	0	0	0
99	0	0	4	0
0	0	0	4	0
0	0	0	0	0
1	1	0	4	0
0	0	0	4	0
1	0	0	4	0
1	0	0	0	0
0	1	0	0	0
99	0	0	0	0
1	0	0	99	99
1	0	0	0	0
0	0	0	0	0
0	0	0	0	0
99	0	0	0	0
1	0	0	0	2
0	0	0	0	2
0	0	0	99	99

0	0	1	99	99
0	0	0	99	99
0	0	0	99	99
1	0	0	2	2
0	0	0	0	2
0	0	0	4	0
1	0	0	4	0
1	0	0	99	99
1	0	0	99	99
0	0	0	2	0
1	0	0	0	2
1	0	0	99	99
0	0	0	99	99
1	0	0	99	99
0	0	0	99	99
0	0	0	99	99
0	0	0	4	2
0	0	0	99	99
1	0	0	0	2
1	0	0	99	99
1	0	0	0	0
0	0	1	0	2
1	0	0	4	0
0	0	0	0	0
0	0	0	0	0
1	0	0	99	99
1	0	0	99	99
1	0	0	99	99
1	1	0	0	0
0	0	0	0	0
0	0	0	4	0
99	0	0	99	99
0	0	0	0	2
1	1	0	99	99
1	0	0	2	0
1	1	0	2	0
0	0	0	0	0
1	0	0	0	0
99	99	0	0	0
1	0	0	1	0
1	0	0	4	2
1	0	0	0	0
1	0	0	0	0
0	0	0	99	99
0	0	0	0	0
0	0	0	99	99
99	0	0	0	0
1	0	0	0	0
1	0	0	0	0
1	0	0	0	2
1	0	0	4	2
0	0	0	99	99
1	0	0	4	2
1	0	0	4	2
1	0	0	4	2
0	0	0	0	0
1	0	0	4	0
0	0	0	1	2
0	0	0	99	99
99	0	0	4	2
1	0	0	1	2
0	0	0	2	2
0	0	0	4	2
99	0	0	2	2
0	0	0	1	2
99	99	1	99	99
1	0	0	1	2
0	0	1	99	99
1	0	0	1	2
1	0	0	4	2
0	0	0	0	2
0	0	0	99	99
0	0	0	99	99
1	0	0	0	2
0	0	0	99	99
0	0	0	0	2
1	0	0	99	99

1	0	0	4	2
0	0	0	0	0
1	0	0	0	0
0	0	0	4	0
1	0	0	99	99
0	0	0	99	99
99	0	0	99	99
1	0	0	1	2
1	0	0	1	2
1	99	0	0	0
1	0	0	0	0
1	0	0	0	0
99	0	0	0	0
1	0	0	0	0
99	0	0	4	2
99	0	0	0	0
99	0	0	99	99
1	0	0	0	0
99	0	1	1	2
0	1	0	0	0
0	0	0	0	2
0	0	0	0	0
1	0	0	1	2
99	0	0	4	2
1	0	0	99	99
0	0	0	99	99
0	0	0	99	99
99	0	0	0	2
1	0	0	0	2
1	0	0	4	2
1	0	0	1	2
1	0	0	0	0
1	0	0	1	2
0	0	0	1	2
1	0	0	0	2
99	99	99	0	2
1	0	1	99	99
99	0	0	99	99
1	0	0	99	99
99	0	0	99	99
1	0	0	0	2
99	1	0	0	2
1	0	0	4	2
1	0	0	4	0
1	0	0	0	0
1	0	0	0	0
1	0	0	0	0
0	0	0	0	2
1	0	0	4	0
1	0	0	99	99
99	0	0	99	99
0	0	0	99	99
1	0	0	4	0
0	99	0	0	2
0	0	0	0	0
1	0	0	0	0
1	99	99	0	2
99	99	0	99	99
99	0	1	99	99
99	0	0	0	2
0	1	0	0	2
1	0	0	4	2
0	0	0	4	2
0	0	0	4	2
0	0	0	4	0
0	0	0	4	2
99	0	1	2	0
1	0	0	0	0
1	0	0	2	0
0	0	0	0	2
0	0	0	99	99
1	0	0	0	0
1	0	0	99	99
1	1	0	0	2
0	0	0	0	0
0	0	0	0	2
99	0	0	0	2

0	0	0	0	2
0	0	0	0	2
0	0	0	99	99
0	0	0	99	99
1	0	0	1	2
0	0	0	0	0
99	0	0	0	0
99	0	0	0	2
0	0	0	0	0
1	0	0	4	2
0	0	0	99	99
0	0	0	99	99
99	0	0	99	99
99	0	0	4	2
0	0	0	1	2
0	0	0	1	2
1	0	1	99	99
1	0	0	0	0
0	0	0	0	0
1	0	0	1	2
1	0	0	4	2
0	0	0	4	2
0	0	0	0	0
0	1	0	2	2
1	0	0	2	2
0	0	0	0	2
1	0	0	4	2
0	0	0	99	99
1	0	0	0	2
0	0	0	1	2
1	0	0	1	2
1	0	0	4	2
99	0	0	99	99
99	0	0	4	2
1	1	0	4	2
0	0	0	0	0
0	0	0	0	0
0	0	0	0	0
1	0	0	2	2
1	0	0	0	0
1	99	0	0	2
0	0	1	99	99
1	0	0	4	2
1	0	0	99	99
0	0	0	99	99
0	0	0	99	99
0	0	1	99	99
0	0	0	99	99
0	0	0	99	99
1	0	0	0	2
1	0	0	4	2
0	0	0	4	2
0	0	0	4	2
99	1	0	1	2
1	0	0	4	2
1	0	0	4	2
1	0	0	4	2
99	0	0	1	2
0	0	0	99	99
0	0	1	99	99
0	0	1	99	99
99	0	0	99	99
1	0	0	1	2
0	1	0	4	2
99	0	0	0	0
1	0	0	0	0
0	0	0	0	0
1	0	0	0	0
1	0	0	0	99
1	0	0	99	99
0	0	0	99	99
0	0	1	99	99
0	0	0	99	99
1	0	0	0	2
1	0	0	0	0
1	0	0	99	99
0	0	0	99	99

0	0	0	99	99
0	0	0	99	99
99	0	0	99	99
0	0	0	1	2
0	0	0	1	2
0	0	0	99	99
0	0	0	4	2
1	0	0	0	2
0	0	0	0	2
0	0	0	0	0
1	0	0	0	2
1	99	0	0	0
99	99	0	4	2
99	99	0	99	99
0	0	0	0	0
0	0	0	0	0
99	0	0	4	2
1	0	0	0	0
0	0	0	2	0
99	1	0	4	0
1	99	0	2	0
99	0	0	99	99
99	0	0	0	0
1	0	0	0	0
1	0	0	0	0
0	0	0	0	0
1	0	0	0	0
1	0	0	0	0
0	0	0	0	0
1	0	0	4	0
1	0	0	0	2
1	0	0	4	2
1	0	0	4	0
1	0	0	0	0
1	0	0	0	0
99	0	0	0	99
0	0	0	0	2
0	0	0	4	2
0	0	1	99	99
1	0	0	0	0
99	0	0	0	0
0	0	0	0	0
1	0	0	4	2
0	0	0	0	0
1	1	0	0	0
0	0	0	0	0
0	0	0	0	0
1	0	0	4	2
1	0	0	4	1
1	0	0	0	0
0	0	0	1	0
1	0	0	0	0
1	0	0	4	0
1	0	0	0	0
1	0	0	4	0
99	99	99	99	99
1	0	0	0	2
1	0	0	0	2
1	0	0	4	2
0	0	0	4	2
1	0	0	0	0
1	0	0	0	0
1	0	0	0	0
1	0	0	4	0
1	1	0	0	99
1	0	0	2	0
1	99	0	0	0
1	99	0	0	0
0	0	0	1	2
0	0	0	1	2
1	0	0	1	2
1	0	0	0	2
99	0	0	4	2
99	99	0	0	2
0	0	0	99	99
1	0	0	0	0
1	0	0	0	99

0	0	0	0	2
0	1	0	0	2
1	0	0	0	0
0	0	0	99	99
0	0	0	99	99
0	0	0	0	2
99	0	0	99	99
1	0	0	4	2
0	0	1	1	2
1	0	0	4	2
0	0	0	0	0
0	0	0	0	0
0	0	0	0	2
0	0	0	4	2
0	0	0	0	0
1	99	0	0	0
0	0	0	4	2
1	0	0	0	0
0	0	0	1	2
0	0	0	1	2
1	0	0	0	2
0	0	0	0	2
0	99	0	99	99
0	0	0	4	2
1	0	1	99	99
1	0	0	0	2
1	0	0	4	0
1	0	0	0	0
1	0	0	0	0
0	0	0	99	99
99	0	1	4	2
0	0	0	0	2
0	0	0	0	2
99	0	0	0	0
99	0	0	0	0
1	0	0	99	99
0	0	0	0	0
0	0	0	99	99
0	0	0	4	2
1	0	0	0	2
1	1	0	99	99
1	1	0	4	2
0	0	0	4	2
1	0	0	4	2
1	0	0	0	0
0	0	1	99	99
0	0	0	99	99
99	0	0	4	2
1	0	0	4	2
0	0	0	0	2
0	0	0	99	99
99	0	0	0	2
1	0	0	99	99
1	0	0	99	99
1	0	0	0	0
0	0	0	0	0
1	0	0	2	2
1	0	0	99	99
0	0	0	99	99
1	0	0	99	99
0	1	0	0	0
1	0	0	0	0
0	0	0	0	2
0	0	0	0	2
0	0	0	4	2
1	0	0	99	99
1	0	0	99	99
1	0	0	99	99
1	0	0	1	2
1	0	0	4	2
1	0	0	99	99
0	0	0	0	2
0	0	0	1	2
99	0	0	4	2
99	0	0	0	2
1	0	0	0	0
1	0	0	2	2

99	0	0	4	2
0	0	0	4	2
0	0	0	0	0
1	0	0	99	99
1	0	0	99	99
1	0	0	0	0
0	0	0	0	0
0	0	0	0	0
0	0	0	0	0
1	0	0	4	2
0	0	0	0	0
1	0	0	0	0
1	0	0	99	99
1	0	0	0	99
1	0	0	99	99
99	0	0	1	2
1	0	0	99	99
0	0	0	0	2
1	0	0	4	2
99	0	0	4	2
1	0	0	0	2
1	1	0	4	2
99	0	0	0	2
1	1	0	4	2
1	0	0	0	2
0	0	0	4	2
99	0	0	99	99
1	0	0	4	2
0	0	0	4	2
0	0	0	0	0
99	0	0	0	2
0	0	0	99	99
1	0	0	0	2
99	0	0	0	2
99	0	0	0	2
1	1	0	0	2
1	0	0	0	2
0	0	0	0	0
1	1	0	0	0
99	0	0	0	2
0	0	0	0	2
1	0	0	0	99
1	0	0	2	0
0	0	0	0	0
0	0	0	0	0
0	0	0	0	0
1	0	0	99	99
1	0	0	99	99
0	0	0	0	0
0	0	0	0	0
1	1	0	0	2
0	0	1	4	2
0	0	1	0	2
0	0	1	99	99
0	0	0	0	2
0	0	1	0	2
0	0	0	99	99
1	0	0	99	99
0	0	0	0	2
0	0	0	99	99
0	0	0	99	99
0	0	0	99	99
99	0	0	4	2
1	0	0	0	2
0	0	0	0	2
0	0	0	4	2
1	0	0	1	2
1	0	0	4	2
1	0	0	0	99
1	0	0	0	2
0	0	0	99	99
1	0	0	0	2
0	0	0	99	99
1	0	0	0	0
1	0	0	0	0
1	0	0	0	99
0	0	0	0	0

1	0	0	99	99
0	0	0	99	99
1	0	0	0	0
1	0	0	0	0
1	0	0	4	0
1	0	0	4	0
1	0	0	0	0
1	0	0	2	0
1	0	0	99	99
99	99	99	1	0
99	99	99	2	0
0	0	0	99	99
1	0	0	0	0
1	0	0	1	2
1	0	0	99	99
1	0	0	0	0
0	0	0	0	0
0	0	0	0	0
1	0	0	0	0
1	0	0	4	0
1	0	0	99	99
0	0	0	99	99
1	0	0	4	0
0	0	0	0	0
0	0	0	0	0
1	0	0	99	99
1	0	0	0	0
0	0	0	99	99
0	0	0	99	99
0	0	0	0	0
0	0	0	0	0
0	0	0	0	2
0	0	1	99	99
99	0	0	4	2
99	0	0	4	2
0	0	0	0	2
0	0	0	1	2
1	0	0	99	99
0	0	0	0	0
1	0	0	2	0
1	0	0	0	0
1	0	0	4	2
1	0	0	0	2
1	0	0	0	2
1	0	0	0	2
99	0	0	4	2
99	0	0	4	2
99	0	0	0	2
1	1	0	0	2
1	0	0	0	99
1	0	0	4	0
1	0	0	0	0
1	0	0	0	99
1	0	0	0	0
0	0	1	99	99
0	0	1	99	99
0	0	0	99	99
1	0	0	99	99
1	0	0	0	2
0	0	0	0	0
1	0	0	0	0
99	0	0	0	1
99	0	0	0	0
1	0	0	0	0
1	0	0	0	2
0	0	0	99	99
0	0	1	99	99
0	0	0	0	0
99	0	0	0	2
0	0	0	0	0
99	0	0	0	0
0	0	0	99	99
1	99	0	99	99
1	99	0	99	99
99	99	0	4	2
1	0	0	0	2
0	0	0	0	0

0	0	0	99	99
99	0	0	0	2
1	0	0	1	2
0	1	0	1	2
0	0	0	1	2
0	0	0	99	99
99	0	1	99	99
1	0	0	0	2
0	0	0	0	2
99	0	0	4	2
1	0	0	0	0
0	0	0	99	99
1	0	0	0	0
0	0	0	0	0
0	0	0	0	0
0	0	0	0	0
1	0	0	4	2
0	0	0	0	2
1	0	0	0	2
0	0	0	4	0
1	0	0	0	0
0	0	0	99	99
1	0	0	0	0
0	0	0	0	2
0	0	0	0	0
1	0	0	0	0
1	1	0	1	2
0	0	0	4	2
0	0	0	2	2
1	0	0	4	2
0	0	0	99	99
1	0	0	4	2
0	0	0	0	2
0	0	0	99	99
0	0	0	0	0
1	0	0	99	99
1	0	0	0	0
1	0	0	99	99
1	0	0	99	99
99	0	0	99	99
1	0	0	0	2
99	99	0	4	2
1	0	0	0	2
0	1	0	0	2
1	0	0	99	99
1	1	0	0	0
0	0	0	99	99
0	0	0	99	99
0	0	0	0	0
0	0	0	0	2
0	0	0	0	2
1	0	0	0	2
0	0	0	99	99
0	0	0	99	99
99	0	0	99	99
1	0	0	0	2
0	0	0	0	2
1	0	0	4	2
0	0	0	0	0
1	0	0	0	0
0	0	0	0	2
0	0	0	0	2
0	0	1	99	99
0	0	1	99	99
99	0	0	4	2
99	0	0	99	99
1	0	0	0	2
1	0	0	0	2
0	0	1	0	2
1	0	0	0	0
1	0	0	4	2
1	0	0	0	0
1	1	0	0	0
1	0	0	0	0
0	0	1	99	99
1	0	0	99	99
1	0	0	99	99

1	1	0	0	0
0	0	0	99	99
1	0	0	99	99
0	0	0	0	0
99	0	0	99	99
99	0	0	99	99
99	0	0	99	99
99	0	0	99	99
1	0	0	0	2
1	0	0	0	2
0	0	0	99	99
0	0	1	99	99
0	0	0	0	2
0	0	0	0	2
0	0	0	99	99
1	0	0	99	99
1	0	0	99	99
1	0	0	4	0
1	0	0	0	0
0	0	0	1	2
0	0	0	0	0
1	0	0	0	0
1	0	0	0	99
0	0	0	99	99
0	0	0	99	99
1	0	0	0	0
1	0	0	0	0
1	0	0	0	99
1	0	0	4	99
1	0	0	0	0
1	0	0	0	0
0	0	0	99	99
0	0	0	99	99
0	0	0	99	99
0	0	0	1	2
1	0	0	0	0
1	1	0	0	2
1	0	0	0	0
0	0	0	0	0
1	0	0	0	0
1	1	0	0	2
0	0	0	0	0
0	0	0	0	2
1	0	0	99	99
1	0	0	1	2
1	0	0	1	2
1	0	0	0	2
0	0	1	0	2
1	1	0	1	2
99	0	0	2	2
0	0	0	4	2
1	0	0	4	2
0	0	0	0	2
99	0	0	99	99
1	0	0	0	0
0	0	0	99	99
99	0	0	99	99
99	0	0	0	0
0	0	0	0	0
0	1	0	99	2
99	99	99	99	99
99	99	99	99	99
99	99	99	1	2
99	99	99	0	2
99	99	99	0	99
0	0	0	0	0
1	0	0	0	0
99	0	0	1	2
99	0	0	99	99
1	0	0	0	0
1	0	0	0	0
1	0	0	4	2
0	0	0	4	2
0	0	0	0	2
0	0	0	4	2
99	0	0	0	2
0	0	0	0	2

0	0	0	0	2
0	0	0	4	2
0	0	0	0	0
1	0	0	0	0
1	0	0	1	0
1	0	0	0	0
0	0	0	1	2
0	0	0	99	99
1	0	0	0	0
0	0	0	99	99
0	0	0	99	99
0	0	0	99	99
1	0	0	0	0
99	0	0	0	0
0	0	0	99	99
1	0	0	99	99
1	1	0	99	99
1	1	0	99	99
0	0	0	0	0
99	0	0	99	99
99	0	0	99	99
0	0	0	0	1
0	0	0	0	0
0	0	0	0	2
1	0	0	0	2
1	0	0	0	0
0	0	0	0	0
0	0	0	4	2
0	0	0	0	0
1	0	0	0	0
99	0	0	0	2
99	0	0	99	99
0	0	0	99	99
0	0	0	99	99
1	1	0	4	2
0	0	0	99	99
0	0	0	99	99
0	0	0	99	99
99	0	0	0	0
1	0	0	0	0
1	0	0	4	0
0	0	0	0	0
1	1	0	0	0
1	0	0	0	0
0	0	0	4	2
0	0	0	0	0
1	0	0	0	0
1	0	0	0	99
1	0	0	99	99
99	0	0	0	2
0	0	1	99	99
0	0	0	99	99
1	0	0	4	2
1	0	0	0	0
0	0	0	1	2
1	0	0	1	2
0	1	0	0	0
0	0	0	0	0
0	0	0	2	0
0	0	0	2	0
0	1	0	4	0
0	0	0	99	99
0	0	0	0	2
0	0	0	0	0
0	0	0	99	99
1	0	0	0	0
0	0	0	4	2
1	0	0	4	2
0	0	0	0	0
1	0	0	0	0
99	0	0	0	0
1	0	0	0	0
0	0	0	99	99
1	0	0	1	2
1	0	0	0	2
99	0	0	99	99
99	0	0	99	99

1	0	0	0	0
0	0	0	99	99
0	0	0	0	0
1	1	0	2	0
0	0	0	99	99
0	0	1	99	99
1	0	0	0	0
99	0	0	99	99
1	0	0	0	0
1	0	0	0	0
1	0	0	0	0
1	0	0	0	0
99	0	0	0	0
99	0	0	0	0
1	0	0	99	99
0	0	0	99	99
99	0	0	4	0
1	0	0	2	0
99	0	0	4	0
1	0	0	0	0
1	0	0	2	0
0	0	0	99	99
0	1	0	99	99
1	0	0	4	0
1	99	0	4	0
0	0	0	4	0
0	0	0	4	0
0	0	0	1	1
0	0	0	0	0
99	99	1	0	0
0	0	0	0	0
1	0	0	0	0
1	0	0	0	0
0	0	0	99	99
0	0	0	99	99
0	0	0	4	2
0	0	0	4	2
1	0	0	0	2
0	0	0	0	2
0	0	0	0	2
0	0	0	0	2
0	0	0	0	2
1	0	0	99	99
1	0	0	99	99
0	0	0	99	99
99	0	0	0	1
0	0	0	99	99
1	0	0	0	2
0	0	0	99	99
99	99	99	99	99
1	0	0	99	99
99	99	0	4	2
99	0	0	0	2
1	0	0	2	2
0	0	0	0	2
0	0	0	99	99
1	0	0	99	99
99	0	0	1	2
0	0	0	1	2
0	0	0	4	2
0	0	0	0	2
0	0	0	4	2
1	0	0	0	2
99	0	0	4	2
1	0	0	99	99
1	0	0	99	99
0	0	0	4	2
99	0	0	0	2
1	0	0	99	99
0	0	0	0	0
1	0	0	0	0
1	0	0	0	2
1	0	0	0	2
99	0	0	0	2
0	0	0	99	99
1	0	0	99	99
1	0	0	4	2

1	1	0	1	2
0	0	0	4	2
1	0	0	4	2
1	0	0	99	99
1	0	0	99	99
0	0	0	2	0
1	1	0	4	0
1	0	0	0	0
1	0	0	99	99
0	99	99	99	99
1	0	0	1	2
0	0	0	4	2
0	0	0	0	0
0	0	0	0	0
0	0	0	0	0
1	0	0	0	0
1	0	0	0	0
0	1	0	4	2
1	0	0	0	2
0	0	0	0	0
1	0	0	0	0
1	0	0	0	0
1	0	0	0	0
1	0	0	0	0
1	1	0	0	0
0	0	0	0	0
1	1	0	4	2
1	0	0	2	0
0	0	0	99	99
1	0	0	0	2
1	0	0	4	2
0	0	0	99	99
1	0	0	4	0
1	1	0	4	0
1	1	0	0	2
99	1	0	0	2
99	0	0	0	2
1	0	0	99	99
99	0	0	99	99
0	0	0	99	99
0	0	0	99	99
1	1	0	0	2
1	0	0	0	2
0	0	1	0	0
0	0	0	99	99
99	0	0	0	0
1	0	1	99	99
1	0	0	0	0
1	0	0	99	99
0	0	0	0	0
0	0	0	4	0
99	0	0	0	0
1	0	0	4	2
0	0	0	99	99
1	0	0	99	99
1	0	0	4	0
0	0	0	0	2
0	0	0	4	2
1	0	0	99	99
0	0	0	99	99
99	0	0	99	99
99	0	0	0	2
99	0	0	99	99
0	0	0	99	99
0	0	0	99	99
0	0	0	0	2
0	0	0	0	2
1	0	0	0	2
0	0	0	0	2
1	0	0	0	2
0	0	0	0	2
99	0	0	0	0
1	0	0	0	0
1	0	0	0	99
1	0	0	99	99
0	0	0	1	2
1	0	0	99	99

0	0	0	99	99
1	0	0	0	2
99	0	0	0	2
99	0	0	99	99
0	0	0	99	99
1	0	1	99	99
1	0	0	0	0
1	0	0	0	0
1	0	0	0	99
1	0	0	0	0
0	0	0	0	0
99	0	0	99	99
0	0	0	0	2
0	0	0	99	99
99	0	0	99	99
0	0	0	0	2
0	0	0	0	0
0	0	0	99	99
0	0	1	99	99
0	0	1	99	99
99	0	0	0	2
0	0	1	99	99
0	0	1	99	99
0	0	1	0	2
0	0	0	0	2
0	0	0	0	2
1	0	0	0	0
1	0	0	0	0
1	0	0	0	0
99	0	0	99	99
1	0	0	99	99
0	0	0	99	99
1	0	0	1	2
0	1	0	1	2
1	0	0	99	99
1	0	0	99	99
99	0	0	99	99
1	0	0	99	99
0	0	0	99	99
0	0	0	99	99
0	0	0	0	0
99	0	0	0	0
1	0	0	0	0
1	0	0	0	0
1	0	0	0	0
1	0	0	0	0
99	0	0	1	0
99	0	0	0	0
0	0	0	2	0
1	0	0	4	0
1	1	0	2	0
1	0	0	0	0
1	0	0	99	99
1	0	0	99	99
0	0	0	0	0
0	0	0	0	0
0	0	0	0	0
0	0	0	0	0
99	0	0	0	0
0	0	0	4	2
0	0	0	99	99
0	0	0	0	0
0	0	1	99	99
0	0	1	99	99
0	0	0	4	0
0	0	0	99	99
1	0	0	0	0
0	0	0	0	2
0	1	0	0	2
99	0	0	99	99
0	0	0	99	99
0	0	0	0	0
1	0	0	4	0
0	0	0	0	0
99	0	1	99	99
1	0	0	99	99
99	0	0	0	0

99	0	0	0	0
99	99	99	99	99
1	0	0	0	0
1	0	0	0	0
0	0	0	0	0
0	0	0	4	2
0	0	0	4	2
1	0	0	0	0
0	0	0	0	0
1	0	0	0	0
99	1	0	2	1
1	99	0	99	99
1	99	0	99	99
1	0	0	99	99
1	0	0	0	0
1	0	0	4	0
1	0	0	0	0
0	0	0	4	0
0	0	0	99	99
1	0	0	0	0
0	0	0	0	0
0	0	0	0	2
0	0	0	0	2
0	0	0	0	0
1	0	0	0	2
1	0	0	99	99
1	0	0	0	0
1	0	0	0	0
99	0	0	0	2
1	0	0	0	0
0	0	0	1	0
0	0	0	4	2
99	0	0	99	99
1	0	0	4	2
0	0	0	0	0
0	0	0	0	2
99	0	0	4	0
0	0	0	0	0
1	0	0	1	0
99	0	0	4	2
0	0	0	4	2
0	0	0	0	2
1	0	0	99	99
1	0	1	99	99
99	0	0	0	0
1	0	0	0	2
1	0	0	0	0
0	0	0	0	0
1	0	0	0	0
1	0	0	0	0
0	0	0	0	0
0	0	0	4	0
1	0	1	99	99
0	0	1	99	99
0	0	1	99	99
0	0	0	4	0
1	99	0	4	0
0	0	0	99	99
1	0	0	0	1
0	0	0	99	99
1	0	0	0	2
1	0	0	4	2
99	1	0	4	2
0	0	0	0	2
1	0	0	4	2
99	99	0	4	2
1	0	0	99	99
1	0	0	0	2
0	0	0	99	99
0	0	0	99	99
0	0	0	99	99
1	1	0	0	2
1	0	0	99	99
1	0	0	99	99
0	0	0	1	2
0	0	0	4	2
1	0	0	0	2

1	0	0	4	2
0	0	0	0	0
0	0	0	99	99
0	0	0	99	99
1	0	0	4	2
0	0	0	0	2
0	0	0	99	99
1	0	0	99	99
99	0	0	0	2
0	0	0	0	2
1	0	0	99	99
1	0	0	0	0
1	0	0	0	0
0	0	0	4	0
1	0	0	4	0
1	0	0	99	99
0	0	0	0	0
0	0	0	0	0
0	0	0	0	0
0	0	0	0	0
1	0	0	0	0
0	0	0	0	0
0	0	0	0	0
99	0	0	0	0
1	0	0	0	0
1	0	0	99	99
0	0	0	1	2
0	0	0	0	2
0	0	0	0	0
1	0	0	0	2
0	0	0	99	99
0	0	0	99	99
0	0	1	0	2
0	0	0	0	2
1	0	0	0	2
1	0	0	0	2
1	0	0	99	99
0	0	0	99	99
0	0	0	99	99
1	0	0	99	99
0	0	0	0	0
0	99	0	99	99
99	0	0	99	99
99	99	0	2	2
0	0	0	4	2
99	0	0	4	2
1	1	1	1	2
99	0	0	4	2
0	0	0	1	2
0	0	0	0	0
1	1	0	4	2
1	0	0	0	2
1	0	0	0	0
0	0	0	1	2
1	0	0	4	2
1	1	0	99	99
0	0	0	0	0
1	0	0	0	0
99	0	0	0	0
0	0	1	99	99
99	0	0	99	99
1	0	0	1	2
1	0	0	0	0
1	1	0	1	0
1	0	0	99	99
0	0	0	4	2
1	0	0	0	0
99	0	0	0	0
1	0	0	4	0
1	0	0	4	2
0	0	0	0	0
99	0	0	99	99
1	0	0	99	99
0	0	0	99	99
1	0	0	99	99
1	0	0	0	0
1	0	0	0	0

1	0	0	1	2
1	1	0	4	2
0	0	0	4	2
0	0	0	4	2
99	0	0	0	2
1	0	0	4	0
0	0	0	0	2
99	0	0	0	0
1	0	0	0	0
1	1	0	0	0
1	0	0	0	0
0	0	1	99	99
99	0	0	99	99
1	0	0	0	0
0	0	0	99	99
1	0	0	0	2
1	0	0	0	0
0	0	0	99	99
99	99	0	0	0
0	0	0	99	99
0	0	0	99	99
0	0	0	99	99
0	0	0	99	99
1	0	0	1	0
99	0	0	1	0
99	0	0	0	2
99	99	0	99	99
0	0	0	99	99
0	0	0	0	0
1	1	0	1	0
0	0	0	99	99
1	1	0	0	0
1	0	0	0	0
1	0	0	99	99
0	0	0	0	0
0	0	0	0	0
1	0	0	0	2
0	0	0	0	0
1	0	0	4	2
99	0	0	1	2
1	0	0	4	2
0	0	0	99	99
0	0	0	4	2
99	0	0	4	2
99	0	0	0	0
99	0	0	99	99
1	0	0	99	99
1	0	0	4	2
1	0	0	0	0
0	0	0	0	0
0	0	0	0	0
1	0	0	4	99
1	1	0	4	99
1	0	0	99	99
1	0	0	99	99
0	0	1	99	99
0	0	0	99	99
1	0	0	4	0
0	0	0	4	0
1	1	0	0	0
1	1	0	0	2
99	0	0	0	0
1	0	0	99	99
0	0	0	99	99
99	0	0	4	2
1	0	0	4	2
1	1	0	0	2
99	0	0	99	99
1	0	0	99	99
99	0	0	0	0
1	0	0	4	2
1	0	0	0	0
0	0	0	99	0
1	1	0	0	0
1	0	0	99	99
0	0	0	99	99
1	0	0	99	99

1	0	0	0	2
0	0	0	0	2
1	0	1	99	99
0	0	0	1	1
0	0	0	4	0
1	0	0	4	2
1	1	0	1	2
0	0	0	99	99
1	0	0	0	0
1	0	0	0	0
1	0	0	0	0
1	0	0	0	0
1	0	0	0	0
1	0	0	99	99
1	0	0	0	0
99	0	0	4	2
1	0	0	4	2
99	99	99	0	2
1	1	0	0	2
0	0	0	1	2
99	0	0	99	99
0	0	1	99	99
1	0	0	99	99
0	0	0	0	0
1	1	0	0	2
0	0	1	99	99
0	1	1	99	99
0	0	1	99	99
1	0	0	0	2
0	0	0	0	2
1	0	0	0	0
1	0	0	99	99
1	0	0	99	99
1	0	0	99	99
1	0	0	99	99
0	0	0	0	2
99	0	0	1	2
1	0	1	99	99
0	0	0	99	99
1	0	0	99	99
1	0	0	99	99
1	0	0	99	99
1	0	0	4	0
0	0	1	4	0
0	0	0	0	0
0	0	0	0	1
0	0	0	99	99
1	0	0	0	2
1	0	0	0	0
1	1	0	4	2
1	0	0	0	1
99	0	0	0	0
1	0	0	0	0
0	0	0	1	2
0	0	0	4	2
0	0	0	99	99
1	0	0	0	0
0	0	0	0	0
1	0	0	0	0
1	0	0	4	2
1	0	0	0	0
0	0	0	0	2
1	0	0	0	0
1	1	0	0	2
0	0	0	0	2
0	0	0	99	99
0	0	0	99	99
1	0	0	0	0
1	0	0	99	99
1	0	0	99	99
99	0	1	4	2
1	0	0	0	2
1	0	0	0	2
0	0	0	0	2
0	0	0	4	2
0	0	1	99	99
0	0	0	0	2

1	0	0	0	0
0	0	0	1	2
0	0	0	4	2
1	0	0	0	0
1	1	0	0	0
1	0	0	0	2
1	0	0	0	0
1	0	0	0	2
1	1	0	0	2
0	0	0	0	0
1	1	0	0	2
1	0	0	4	2
1	0	0	0	2
1	0	0	0	2
1	0	0	99	99
1	0	0	0	2
0	0	0	99	99
0	0	0	99	99
1	0	0	99	99
1	1	0	99	99
0	0	0	4	2
1	0	0	2	0
1	1	0	4	0
1	0	0	99	99
1	0	0	99	99
1	1	0	99	99
1	1	0	0	0
1	0	0	0	0
1	0	0	0	0
1	0	0	4	2
1	0	0	99	99
0	0	0	0	2
0	1	0	0	0
1	1	0	0	0
1	0	0	0	0
1	0	0	0	0
1	0	1	99	99
1	0	0	99	99
1	0	0	0	0
99	0	0	99	99
1	0	0	99	99
0	0	0	4	2
1	0	0	0	0
99	0	0	2	2
1	0	0	4	2
0	0	0	1	2
0	0	0	99	99
1	1	0	99	99
0	0	0	99	99
99	0	0	0	2
1	0	0	4	2
1	0	0	0	2
1	0	0	0	0
1	0	0	0	2
0	0	0	0	2
1	0	0	0	0
1	0	0	0	0
1	0	0	0	0
1	0	0	0	0
1	1	0	0	0
1	0	0	99	99
0	0	0	0	0
1	0	0	99	99
1	99	99	99	99
99	0	0	0	0
0	0	0	0	0
1	0	0	1	2
99	0	0	4	2
0	0	0	99	99
0	1	0	1	2
1	0	0	99	99
1	0	0	0	0
0	0	0	0	0
0	0	0	0	2
1	0	0	4	2
0	0	0	0	0
0	0	0	0	0

1	1	1	4	2
0	0	0	0	1
99	0	0	99	99
0	0	0	99	99
99	1	0	99	99
0	0	0	0	2
0	1	0	99	99
0	1	0	0	2
0	0	0	0	2
0	0	0	0	1
0	0	0	99	99
0	0	0	4	2
0	0	0	4	2
0	0	0	0	2
0	0	0	0	1
1	0	0	4	2
1	0	0	4	1
1	0	0	0	0
0	0	0	99	99
0	0	0	99	99
99	0	0	0	2
1	0	0	0	0
0	0	0	0	0
99	0	0	0	0
1	0	0	1	2
1	1	0	4	0
1	0	0	0	0
0	0	0	0	0
1	0	0	0	0
0	0	0	0	0
1	0	0	0	0
0	0	0	99	99
1	0	0	0	0
1	0	0	0	0
0	0	0	0	0
1	0	0	0	0
1	0	0	0	0
0	0	0	0	2
1	0	0	0	0
0	0	0	0	0
0	0	0	0	0
99	0	0	4	0
1	1	0	0	0
1	1	0	99	99
0	0	0	99	99
0	0	0	99	99
1	0	0	0	0
1	0	0	0	1
0	0	0	0	0
1	0	0	0	0
1	1	0	0	0
0	0	0	0	0
1	0	0	99	99
0	0	0	99	99
0	0	0	99	99
0	0	0	99	99
0	0	0	99	99
99	0	0	0	0
1	1	0	4	2
1	0	0	0	99
1	0	0	0	0
0	1	0	0	2
0	0	0	99	99
0	0	0	99	99
0	0	99	99	99
1	0	0	99	99
99	1	0	0	0
1	0	0	0	0
1	0	0	0	99
0	0	0	0	2
1	1	0	4	0
1	0	0	0	0
1	0	0	0	0
1	0	0	0	0
0	1	0	0	0
0	0	0	99	99
0	0	0	99	99

0	1	0	0	2
1	0	0	0	2
0	0	0	0	0
1	0	0	0	2
0	0	0	99	99
0	0	0	0	2
0	0	0	0	2
0	1	0	0	2
1	0	0	0	2
1	1	0	4	2
1	0	0	0	2
0	0	0	0	2
0	0	0	99	99
0	0	0	0	2
1	0	0	0	2
0	0	0	99	99
0	0	0	99	99
0	0	0	99	99
0	1	0	4	2
1	0	0	4	2
1	0	0	99	99
1	0	0	99	99
0	0	0	0	0
1	0	0	0	0
1	0	0	0	0
0	0	0	0	0
99	99	0	2	0
1	0	0	99	99
0	0	0	99	99
0	0	0	99	99
1	0	0	99	99
99	0	0	4	0
1	0	0	0	0
1	0	0	0	0
1	0	0	0	0
1	0	0	4	0
1	0	0	0	0
1	0	0	0	0
1	1	0	2	0
1	0	0	99	99
0	0	0	99	99
0	0	0	0	2
1	1	0	4	2
0	0	0	1	2
1	0	0	4	0
1	1	0	0	2
0	0	0	0	2
1	0	0	0	2
0	0	0	0	2
0	0	0	0	0
1	1	0	0	0
0	0	0	0	2
1	0	0	0	99
0	0	0	0	0
0	0	0	0	0
1	0	0	0	0
1	0	0	0	0
0	0	0	0	0
0	0	0	0	0
0	0	0	0	0
0	0	0	0	0
1	1	0	0	0
1	0	0	0	0
1	1	0	4	2
0	0	0	0	0
0	0	0	0	0
0	0	0	4	0
1	0	0	0	0
1	0	0	0	0
1	0	0	99	99
1	0	0	99	99
0	0	0	99	99
1	0	0	0	0
0	0	0	99	99
1	0	0	0	0
0	0	0	0	0
99	1	1	1	2

1	0	0	0	2
1	0	0	99	99
1	0	0	99	99
0	0	0	99	99
99	0	0	4	2
0	0	0	99	99
1	0	0	0	0
99	99	0	99	99
0	0	0	0	0
1	0	0	0	0
1	0	0	0	2
0	0	0	1	2
0	0	0	4	2
99	0	0	99	99
0	0	0	4	2
0	0	0	1	2
0	1	0	99	99
1	0	0	0	0
0	0	0	99	99
0	0	0	0	0
1	0	0	0	0
0	0	0	0	0
1	0	0	0	0
0	0	0	0	0
0	0	0	99	99
1	0	0	0	1
1	0	0	0	0
1	0	0	99	99
1	0	0	4	2
1	0	0	0	99
1	1	0	0	99
1	0	0	0	0
1	0	0	0	99
99	0	0	1	2
99	0	0	1	2
1	0	0	99	99
0	0	0	99	99
99	0	0	1	2
1	0	0	0	0
1	0	0	0	0
1	0	0	0	0
1	0	0	0	0
1	0	0	0	0
1	0	0	0	0
1	0	0	4	2
0	0	0	99	99
0	0	0	99	99
99	0	0	4	2
0	0	0	4	2
1	0	0	0	0
0	0	0	0	2
0	0	0	0	2
1	0	0	99	99
1	0	0	99	99
1	0	0	0	0
1	0	0	0	0
1	0	0	0	0
0	0	0	0	0
0	0	0	4	0
0	0	0	99	99
0	0	0	0	0
1	0	0	0	0
1	0	0	0	0
0	0	0	99	99
1	0	0	0	0
99	0	0	0	0
99	0	0	0	0
0	0	0	1	2
0	0	0	4	2
0	0	0	0	2
0	0	0	0	2
0	0	0	99	99
1	0	0	0	2
1	0	0	0	2
0	0	0	4	2
1	0	0	0	0
1	0	0	0	0

1	0	0	0	0
0	0	0	0	0
99	0	0	0	2
99	0	0	4	2
1	0	0	0	2
0	0	0	0	2
99	0	1	4	2
99	0	0	0	2
99	0	0	4	2
1	0	0	0	0
1	0	0	0	0
0	0	0	0	0
1	0	0	0	0
0	0	0	99	99
1	0	0	99	99
1	0	0	4	2
1	0	0	0	0
0	0	0	0	0
1	0	0	0	0
1	0	0	99	99
1	0	0	1	2
1	1	0	0	0
1	0	0	99	99
1	0	0	99	99
99	0	0	0	99
99	0	0	1	2
0	0	0	0	0
1	0	0	99	99
1	0	0	99	99
0	0	0	0	0
1	0	0	0	0
1	0	0	0	0
1	0	0	0	2
1	1	0	1	2
0	0	0	0	0
0	0	0	99	99
1	0	0	99	99
0	0	0	99	99
1	1	0	0	0
1	0	0	0	0
1	0	0	0	0
1	0	0	0	0
99	0	0	0	0
1	0	0	99	99
1	0	0	99	99
1	0	0	99	99
1	0	0	99	99
0	1	0	0	2
1	0	0	0	2
99	0	0	4	2
0	0	0	99	99
0	0	0	0	2
0	0	1	99	99
0	0	1	99	99
0	0	0	0	0
0	0	0	0	2
0	0	0	99	99
0	0	0	99	99
0	0	0	99	99
99	0	0	99	99
0	0	0	99	99
0	1	0	0	2
1	0	0	0	0
1	0	0	0	0
1	0	0	0	0
1	99	99	99	99
99	99	99	99	99
1	99	99	99	99
99	99	99	99	99
0	0	0	0	2
1	99	99	99	99
0	0	0	4	2
1	0	0	0	0
1	0	0	0	0
1	0	0	0	0
1	0	0	0	0
1	1	0	0	0

1	1	0	0	0
1	0	0	99	99
0	0	0	4	2
1	0	0	99	99
1	0	0	99	99
0	0	0	99	99
1	0	1	99	99
99	0	0	0	2
1	0	0	0	2
0	0	0	99	99
1	1	0	4	2
0	0	0	2	2
0	1	0	1	2
1	0	0	0	2
0	0	0	0	2
0	0	0	99	99
99	0	0	99	99
0	0	0	99	99
0	0	0	99	99
1	1	0	4	2
99	0	0	4	2
1	0	0	0	2
1	0	0	0	2
0	0	0	0	2
99	0	0	99	99
99	0	0	99	99
1	0	0	0	2
0	0	0	0	0
0	0	0	99	99
1	0	0	1	2
0	0	0	0	2
0	0	0	0	0
0	0	0	99	99
1	0	0	99	99
0	0	0	0	0
1	0	0	0	2
0	0	0	0	2
1	0	0	99	99
0	0	0	0	2
0	0	0	0	2
0	0	0	0	0
0	0	0	1	2
0	0	0	0	0
1	1	1	0	0
1	0	0	4	2
0	0	0	99	99
1	0	0	0	0
1	0	0	99	99
0	0	0	0	0
1	0	0	0	0
1	0	0	0	0
1	0	0	0	0
0	1	0	0	0
1	0	0	4	0
1	0	0	0	0
1	0	0	0	0
0	0	1	99	99
1	0	0	0	0
0	0	0	4	2
99	0	0	0	0
0	0	0	99	99
1	0	0	4	2
0	0	0	0	2
99	0	0	0	0
0	0	0	99	99
0	1	0	4	2
0	0	0	4	2
0	0	0	0	0
0	0	0	0	2
0	0	0	99	99
0	0	0	4	2
0	0	0	0	0
0	0	0	0	2
0	0	0	99	99
0	0	0	99	99
1	0	0	4	2
0	0	0	0	0

1	0	0	4	0
1	0	0	0	0
1	0	0	0	0
0	0	0	0	0
1	0	0	0	0
0	0	0	1	2
0	0	0	0	0
1	0	0	99	99
0	0	0	1	2
1	0	0	0	0
1	0	0	99	99
0	0	0	99	99
0	0	0	99	99
1	0	0	99	99
1	0	0	0	2
1	0	0	4	2
1	0	0	0	0
1	0	0	99	99
1	0	0	0	0
0	0	0	0	0
1	1	0	1	2
0	0	0	4	2
0	0	0	0	0
1	0	0	0	0
1	0	0	1	2
1	0	0	4	2
1	0	0	0	99
0	0	0	0	0
1	0	0	0	0
1	0	0	0	0
1	0	0	0	0
0	0	0	0	0
1	0	0	0	2
1	1	0	99	99
0	1	0	0	0
1	0	0	0	0
1	0	0	0	0
1	0	0	0	0
1	0	0	0	0
1	0	0	99	99
99	0	0	99	99
99	0	0	99	99
1	0	0	0	0
99	0	0	4	0
99	1	0	0	0
1	0	0	0	0
0	0	0	0	0
1	0	0	0	0
1	0	0	0	0
99	0	0	0	2
99	0	0	0	0
1	0	0	0	0
1	0	0	4	0
1	0	0	0	0
0	0	0	0	0
1	0	0	0	0
1	0	0	0	0
1	0	0	0	0
1	0	0	0	99
1	0	0	0	99
1	0	0	0	99
1	0	0	0	0
1	1	0	1	0
0	0	0	0	0
99	1	0	0	0
0	0	0	0	0
0	0	0	99	99
0	0	0	99	99
0	0	0	0	0
1	0	0	0	0
0	0	0	0	0
99	0	0	0	2
99	0	0	99	99
0	0	0	99	99
0	0	0	99	99
1	1	0	99	2
0	0	0	99	99

0	0	0	99	99
1	0	0	99	99
0	0	0	99	99
99	99	99	99	99
99	99	99	99	99
99	99	99	4	2
0	1	0	0	2
1	0	0	0	2
99	0	0	99	99
0	0	0	99	99
1	0	1	99	99
1	0	0	2	99
1	1	0	2	0
0	0	0	0	0
1	0	0	0	0
1	0	0	0	0
1	0	0	0	0
99	0	0	0	2
99	0	0	0	2
0	0	0	99	99
0	0	0	99	99
1	0	0	0	0
0	0	0	0	0
1	0	0	0	0
99	0	0	0	0
0	0	0	0	0
1	0	0	4	0
99	0	0	4	2
99	0	0	4	2
0	0	0	0	0
1	1	0	4	0
1	0	0	0	0
0	0	0	0	0
99	0	0	4	0
0	0	0	0	0
1	0	0	99	99
1	0	0	99	99
1	0	0	0	0
1	0	0	0	2
0	0	0	99	99
1	0	0	0	2
99	99	0	0	0
99	0	0	0	0
99	0	0	0	2
99	0	0	99	99
1	1	0	0	2
99	0	0	4	2
1	0	0	0	2
1	0	0	1	2
1	0	0	0	99
1	0	0	4	2
1	0	0	4	2
0	1	0	99	99
1	0	0	0	2
0	0	0	0	0
1	0	0	0	0
0	0	0	0	0
1	0	0	0	0
1	0	0	4	2
0	0	0	4	2
1	0	0	99	99
1	0	0	99	99
1	0	0	99	99
1	0	0	4	2
0	0	0	0	2
0	0	0	0	2
0	0	0	99	99
1	0	0	99	99
1	0	0	99	99
1	0	0	99	99
99	0	0	4	2
99	99	99	99	99
99	99	99	99	99
99	99	99	99	99
99	99	99	99	99
99	99	99	99	99
99	99	99	99	99

99	99	99	99	99
99	99	99	99	99
99	99	99	99	99
99	99	99	99	99
99	99	99	99	99
99	99	99	99	99
99	99	99	99	99
99	99	99	99	99
99	99	99	99	99
99	99	99	99	99
99	99	99	99	99
99	99	99	99	99
99	99	99	99	99
99	99	99	99	99
99	99	99	99	99
99	99	99	99	99
99	99	99	99	99
99	99	99	99	99
99	99	99	99	99
99	99	99	99	99
99	99	99	99	99
99	99	99	99	99
99	99	99	99	99
99	99	99	99	99
99	99	99	99	99
99	99	99	99	99
99	99	99	99	99
99	99	99	99	99
99	99	99	99	99
99	99	99	99	99
99	99	99	99	99
99	99	99	99	99
99	99	99	99	99
99	99	99	99	99
99	99	99	99	99
99	99	99	99	99
99	99	99	99	99
99	99	99	99	99
99	99	99	99	99
99	99	99	99	99
99	99	99	99	99
99	99	99	99	99
99	99	99	99	99
99	99	99	99	99
99	99	99	99	99
99	99	99	99	99
99	99	99	99	99
99	99	99	99	99
99	99	99	99	99
99	99	99	99	99
99	99	99	99	99
99	99	99	99	99
99	99	99	99	99
99	99	99	99	99
99	99	99	99	99
99	99	99	99	99
99	99	99	99	99
99	99	99	99	99
99	99	99	99	99
99	99	99	99	99
99	99	99	99	99
99	99	99	99	99
99	99	99	99	99
99	99	99	99	99
99	99	99	99	99
99	99	99	99	99
99	99	99	99	99
99	99	99	99	99
99	99	99	99	99
99	99	99	99	99
99	99	99	99	99
99	99	99	99	99
99	99	99	99	99
99	99	99	99	99
99	99	99	99	99
99	99	99	99	99
99	99	99	99	99

99	99	99	99	99
99	99	99	99	99
99	99	99	99	99
99	99	99	99	99
99	99	99	99	99
99	99	99	99	99
99	99	99	99	99
99	99	99	99	99
99	99	99	99	99
99	99	99	99	99
99	99	99	99	99
99	99	99	99	99
99	99	99	99	99
99	99	99	99	99
99	99	99	99	99
99	99	99	99	99
99	99	99	99	99
99	99	99	99	99
99	99	99	99	99
99	99	99	99	99
99	99	99	99	99
99	99	99	99	99
99	99	99	99	99
99	99	99	99	99
99	99	99	99	99
99	99	99	99	99
99	99	99	99	99
99	99	99	99	99
99	99	99	99	99
99	99	99	99	99
99	99	99	99	99
99	99	99	99	99
99	99	99	99	99
99	99	99	99	99
99	99	99	99	99
99	99	99	99	99
99	99	99	99	99
99	99	99	99	99
99	99	99	99	99
99	99	99	99	99
99	99	99	99	99
99	99	99	99	99
99	99	99	99	99
99	99	99	99	99
99	99	99	99	99
99	99	99	99	99
99	99	99	99	99
99	99	99	99	99
99	99	99	99	99
99	99	99	99	99
99	99	99	99	99
99	99	99	99	99
99	99	99	99	99
99	99	99	99	99
99	99	99	99	99
99	99	99	99	99
99	99	99	99	99
99	99	99	99	99
99	99	99	99	99
99	99	99	99	99
99	99	99	99	99
99	99	99	99	99
99	99	99	99	99
99	99	99	99	99
99	99	99	99	99
99	99	99	99	99
99	99	99	99	99
99	99	99	99	99
99	99	99	99	99
99	99	99	99	99
99	99	99	99	99
99	99	99	99	99
99	99	99	99	99
99	99	99	99	99
99	99	99	99	99
99	99	99	99	99
99	99	99	99	99

99	99	99	99	99
99	99	99	99	99
99	99	99	99	99
99	99	99	99	99
99	99	99	99	99
99	99	99	99	99
99	99	99	99	99
99	99	99	99	99
99	99	99	99	99
99	99	99	99	99
99	99	99	99	99
99	99	99	99	99
99	99	99	99	99
99	99	99	99	99
99	99	99	99	99
99	99	99	99	99
99	99	99	99	99
99	99	99	99	99
99	99	99	99	99
99	99	99	99	99
99	99	99	99	99
99	99	99	99	99
99	99	99	99	99
99	99	99	99	99
99	99	99	99	99
99	99	99	99	99
99	99	99	99	99
99	99	99	99	99
99	99	99	99	99
99	99	99	99	99
99	99	99	99	99
99	99	99	99	99
99	99	99	99	99
99	99	99	99	99
99	99	99	99	99
99	99	99	99	99
99	99	99	99	99
99	99	99	99	99
99	99	99	99	99
99	99	99	99	99
99	99	99	99	99
99	99	99	99	99
99	99	99	99	99
99	99	99	99	99
99	99	99	99	99
99	99	99	99	99
99	99	99	99	99
99	99	99	99	99
99	99	99	99	99
99	99	99	99	99
99	99	99	99	99
99	99	99	99	99
99	99	99	99	99
99	99	99	99	99
99	99	99	99	99
99	99	99	99	99
99	99	99	99	99
99	99	99	99	99
99	99	99	99	99
99	99	99	99	99
99	99	99	99	99
99	99	99	99	99
99	99	99	99	99
99	99	99	99	99
99	99	99	99	99
99	99	99	99	99
99	99	99	99	99
99	99	99	99	99
99	99	99	99	99
99	99	99	99	99
99	99	99	99	99
99	99	99	99	99
99	99	99	99	99
99	99	99	99	99
99	99	99	99	99
99	99	99	99	99
99	99	99	99	99

99	99	99	99	99
99	99	99	99	99
99	99	99	99	99
99	99	99	99	99
99	99	99	99	99
99	99	99	99	99
99	99	99	99	99
99	99	99	99	99
99	99	99	99	99
99	99	99	99	99
99	99	99	99	99
